# A Pyrazole-Based Small Molecule, KB3409, Restores Mitochondrial Function via VCP Activation and RCN2-Dependent Ca^2+^ Regulation

**DOI:** 10.3390/antiox15081006

**Published:** 2026-08-13

**Authors:** Yoo Jin Lee, Jee Hee Yoon, Hyunwoong Lim, Eun Seon Song, Ji Ho Park, Dongwoo Kim, Ji-eun Yang, Moon Kyoung So, Minseon Kim, Jihyun Song, Sehui Lim, Hyung Wook Kwon, Youngjoo Byun, Joon Tae Park

**Affiliations:** 1 Division of Life Sciences, College of Life Sciences and Bioengineering, Incheon National University, Incheon 22012, Republic of Korea; juli9709@inu.ac.kr (Y.J.L.); yoojn0905@inu.ac.kr (J.H.Y.); sos0026425@inu.ac.kr (E.S.S.); 202428002@inu.ac.kr (J.H.P.); mk.so@thermofisher.com (M.K.S.); alstjs0323@inu.ac.kr (M.K.); jhsong@inu.ac.kr (J.S.); limsehui1028@inu.ac.kr (S.L.); hwkwon@inu.ac.kr (H.W.K.); 2 College of Pharmacy, Korea University, Sejong 30019, Republic of Korea; hyunwoong@korea.ac.kr (H.L.); 2019290022@korea.ac.kr (D.K.); yangji2011@korea.ac.kr (J.-e.Y.); 3 Interdisciplinary Major Program in Innovative Pharmaceutical Sciences, Korea University, Sejong 30019, Republic of Korea; 4 Convergence Research Center for Insect Vectors, Incheon National University, Incheon 22012, Republic of Korea

**Keywords:** ROS, senescence rejuvenation, KB3409, VCP, RCN2

## Abstract

Cellular senescence is characterized by the accumulation of reactive oxygen species (ROS), and the selective elimination of excessive ROS remains a key therapeutic challenge. Through antioxidant screening, we identified the pyrazole-based small molecule KB3409 as a potent regulator that reduces ROS levels in senescent fibroblasts. To elucidate its mechanism of action, we performed target identification and found that KB3409 directly binds to valosin-containing protein (VCP) and reticulocalbin-2 (RCN2). Functionally, KB3409 acts as an allosteric activator of VCP, enhancing its ATPase activity and promoting autophagic flux. This activation facilitates the selective clearance of dysfunctional mitochondria, thereby improving mitochondrial quality control and limiting ROS generation at its source. Concurrently, KB3409 modulates RCN2-dependent Ca^2+^ homeostasis, alleviating mitochondrial Ca^2+^ overload and suppressing the opening of the mitochondrial permeability transition pore (mPTP). This coordinated regulation preserves mitochondrial structural integrity and sustains oxidative phosphorylation efficiency. Collectively, these findings identified the novel mechanism in which KB3409 restores mitochondrial function, reduces ROS levels, and functionally reverses cellular senescence phenotypes.

## 1. Introduction

Various stressors, such as oxidative stress, DNA damage, and tumorigenic signals, induce cellular senescence, leading to a gradual decline in the function of cellular organelles [1]. Among these organelles, mitochondria are known to be particularly vulnerable to structural and functional changes throughout the senescence process [2]. Mitochondrial dysfunction increases electron leakage in the electron transport chain (ETC), which promotes the generation of ROS [3]. Excessive ROS causes damage to various cellular components, including mitochondria, resulting in a vicious cycle that exacerbates functional decline [4]. The accumulation of such oxidative damage is a major cause of the progression of senescence. Therefore, restoring mitochondrial function has emerged as a key strategy in anti-aging research, yet effective approaches to control mitochondrial function and prevent aging remain limited.

Heterocyclic aromatic motifs, including oxazoles and pyrazoles, have emerged as important scaffolds in drug development due to their broad spectrum of biological activities and structural adaptability [5,6]. Recently, oxazole and pyrazole analogs have been found to demonstrate anti-aging efficacy. For example, the oxazole-quinoline derivative (KB1541) has been shown to promote ATP synthase 5α/β dimerization by binding to the 14-3-3ζ protein and inducing phosphorylation at serine 58 [7]. This increase enhances oxidative phosphorylation (OXPHOS) efficiency and ATP production, ultimately restoring mitochondrial function and alleviating aging-related phenotypes [7]. Another oxazole-based compound, HUP-55, has demonstrated therapeutic effects in an aging-related Parkinson’s disease model by crossing the blood–brain barrier and inhibiting α-synuclein dimerization [8]. At the same time, pyrazole-containing compounds have been included in several clinically approved drugs with anticancer, anti-obesity, and analgesic effects [9]. In addition to these applications, some pyrazole derivatives have been shown to regulate oxidative stress by reducing ROS production in a structure-dependent manner [10].

Valosin-containing protein (VCP), a member of the ATPase family, is essential for maintaining cellular proteostasis by regulating ATPase-dependent protein remodeling processes [11]. VCP is known to harbor an allosteric regulatory site in addition to its canonical nucleotide-binding (ATP/ADP) pocket. This site functions as a critical regulatory hub that modulates conformational dynamics and ATPase activity. Structurally, the allosteric pocket is defined by a hydrophobic core formed by VAL617, PHE618, and PRO510, complemented by an extensive hydrogen-bonding network involving LYS614, ASN616, and THR613 [12]. VCP has emerged as a key regulator of autophagic flux, facilitating the maturation of autophagosomes and the clearance of ubiquitinated substrates, including damaged mitochondria [13]. Dysregulation of VCP activity impairs autophagic flux, leading to the accumulation of defective organelles and exacerbation of oxidative stress, thereby contributing to the progression of cellular senescence [14].

Calcium ions (Ca^2+^) play a central role in diverse cellular processes, including enzyme regulation and apoptosis, making precise control of intracellular Ca^2+^ levels essential for maintaining metabolic homeostasis [15]. The endoplasmic reticulum (ER) serves as the primary intracellular Ca^2+^ reservoir, regulating its distribution to other organelles, including mitochondria [16]. Disruption of ER Ca^2+^ homeostasis can therefore lead to widespread cellular dysfunction. Calcium transfer from the ER to mitochondria occurs at mitochondria-associated ER membranes, which establish functional contact sites between the two organelles [17]. Reticulocalbin-2 (RCN2), an ER-resident Ca^2+^-binding protein containing multiple EF-hand domains, contributes to the regulation of intracellular Ca^2+^ homeostasis and may influence Ca^2+^ dynamics at ER–mitochondria contact sites [18]. Excessive Ca^2+^ accumulation within mitochondria impairs mitochondrial function and promotes the overproduction of ROS [18]. An increase in calcium concentration within mitochondria opens mitochondrial permeable transition pores (mPTP), resulting in loss of mitochondrial integrity and induction of apoptosis [19]. Accordingly, tight regulation of Ca^2+^ homeostasis across intracellular compartments is critical for preserving mitochondrial function and delaying the onset of cellular senescence.

In this study, we aimed to identify compounds capable of suppressing ROS generation using a library of oxazole and pyrazole analogs. Through this screening, we identified candidate molecules that enhance mitochondrial quality control by modulating VCP activation while concurrently regulating RCN2-dependent pathways involved in endoplasmic reticulum–mitochondria Ca^2+^ transfer. Based on these findings, we propose a novel mechanism by which coordinated regulation of mitochondrial proteostasis and Ca^2+^ homeostasis limits ROS production and alleviates cellular senescence.

## 2. Materials and Methods

### 2.1. Procedure for the Synthesis of KB3409 and Biotinylated KB3409

Detailed synthetic procedures and conditions for KB3409 and biotinylated KB3409 are provided in the Supplementary Information (Figures S1–S9).

### 2.2. Cell Culture

Human dermal fibroblasts (PCS-201-010; ATCC, Manassas, VA, USA) were cultured under the conditions described in Table 1. To distinguish senescent fibroblasts from young fibroblasts, criteria including population doubling time (PDT), cell morphology, and senescence-associated β-galactosidase (SA-β-Gal) positivity were used Senescent fibroblasts were defined as cells with PDT of 14 days or longer, whereas young fibroblasts were defined as cells with PDT of less than 2 days [20,21,22,23]. Young fibroblasts exhibited a thin, spindle-shaped morphology (red arrows), whereas senescent fibroblasts were broad and flattened (dotted lines) (Figure S10A). In addition, fibroblasts were classified as senescent when their SA-β-Gal positivity was 59% or higher and as young fibroblasts when it was less than 3% [23] (Figure S10B).

### 2.3. Compound Screening

Briefly, 2000 senescent fibroblasts per well were seeded into 96-well plates. Library components were added every 4 days, diluted to a final concentration of 8 µM. As in the previous study [24], ROS levels were evaluated using high-throughput ROS detection technology after drug administration for 12 days. The mean and standard deviation of 6 replicate experiments were calculated for each experimental group.

### 2.4. Measurement of the Solubility of Compounds, Including KB3409

To determine the aqueous solubility of KB3409, an excess amount of the compound was added to 1 mL of phosphate-buffered saline (PBS; LB004-02; Welgene, Gyeongsan, Republic of Korea). The suspension was sonicated for 2 h and subsequently equilibrated to room temperature. After equilibration, the suspension was filtered, and the concentration of dissolved KB3409 in the filtrate was quantified by high-performance liquid chromatography (HPLC; 1260 Infinity; Agilent Technologies, Santa Clara, CA, USA).

For quantification, a five-point calibration curve was prepared using KB3409 standard solutions at concentrations of 0.5, 1, 5, 10, and 50 μM. The standards were prepared by diluting a 10 mM KB3409 stock solution in DMSO with a 1:1 (*v*/*v*) mixture of acetonitrile and distilled water. The calibration curve was generated by plotting the HPLC peak area against the corresponding KB3409 concentration (Figure S11). The resulting linear regression equation was y = 16.94x − 6.6078, with a coefficient of determination of R^2^ = 0.999.

The HPLC peak area of KB3409 in the filtered PBS sample was 15.60 mAU·min, corresponding to an estimated dissolved concentration of 1.311 μM. Because the measured solubility of KB3409 was higher than the concentration used in the biological experiments (1 μM), KB3409 was considered sufficiently soluble under the assay conditions.

The experimentally determined solubility of KB3409 was also expressed as LogS, defined as the base-10 logarithm of the molar solubility. The measured solubility of 1.311 μM corresponds to 1.311 × 10^−6^ mol/L and a LogS value of approximately −5.88: LogS = log_10_(1.311 × 10^−6^ mol/L) ≈ −5.88.

This experimentally determined value was comparable to the LogS value predicted using ChemDraw (version 22.2.0; CambridgeSoft, Cambridge, MA, USA; predicted LogS = −6.174). Because the initial screening included a large number of compounds, the aqueous solubility of each compound was not experimentally determined. Instead, the predicted LogS values for all screened compounds were calculated using ChemDraw. The predicted LogS values, together with the molecular weights and estimated aqueous solubilities of the compounds, are summarized in Table S1.

### 2.5. Measurement of the Excitation and Emission Wavelengths of KB3409

The excitation and emission spectra of KB3409 were measured using a fluorescence spectrophotometer (F-7000; Hitachi, Tokyo, Japan). The wavelengths corresponding to the maximum fluorescence intensities were identified as the maximum excitation and emission wavelengths of KB3409.

### 2.6. Cell Proliferation Assay

Senescent fibroblasts were treated with KB3409 (0–4 µM) or dimethyl sulfoxide (DMSO; 0.01%; 12767-500ML-SR; Duksan, Ansan, Republic of Korea) at 4-day intervals for 12 days. Cell proliferation was assessed on day 12 using the EZ-Cytox reagent (EZ-500; DoGenBio, Seoul, Republic of Korea), a water-soluble tetrazolium-based assay, according to the manufacturer’s instructions.

### 2.7. Viability Assay

For 12 days, senescent fibroblasts were treated with KB3409 (1 μM) or DMSO (0.01%) at 4-day intervals. After treatment, 0.5 mg/mL of 3-(4,5-dimethylthiazol-2-yl)-2,5-diphenyltetrazolium bromide (V-13154, Thermo Fisher Scientific, Waltham, MA, USA) was added to fresh medium and incubated at 37 °C for 4 h. The resulting formazan crystals were dissolved in 1 mL of DMSO, and the absorbance was measured at 570 nm using a Varioskan LUX microplate reader (Thermo Fisher Scientific).

### 2.8. Flow Cytometric Analysis of ROS, Mitochondrial Mass, Mitochondrial Membrane Potential (MMP), and Autophagy Flux

Senescent fibroblasts were treated with DMSO (0.01%) or KB3409 (1 μM) at 4-day intervals for 12 days. Then, cells were treated in media containing each dye for 30 min at 37 °C in order to assess ROS, mitochondrial mass, MMP, and autophagy flux (Table 2). Specifically, autophagy flux was assessed by treatment with 20 μM chloroquine (CQ; C6628; Sigma, St. Louis, MO, USA) for 24 h prior to analysis, with untreated cells serving as controls. A CytoFLEX flow cytometer (Beckman Coulter Life Sciences, Indianapolis, IN, USA) was used to examine the cells.

### 2.9. Neutral Comet Assay

For 12 days, senescent fibroblasts were treated with either DMSO (0.01%) or KB3409 (1 μM) every 4 days. The CometAssay Single Cell Gel Electrophoresis Assay Kit (4250–050–K; R&D systems, Minneapolis, MN, USA) was used to measure DNA tail lengths. All experimental procedures followed the manufacturer’s instructions.

### 2.10. IM–MS/MS TOF Analysis

Senescent fibroblasts were exposed to either 4 µM biotin or 4 µM biotinylated KB3409 for 14 days. AccuNanoBeadTM streptavidin magnetic nanobeads (TA–1015–1; Bioneer, Daejeon, Republic of Korea) were used for immunoprecipitation. The eluted proteins were analyzed using an ion mobility tandem mass spectrometer/time-of-flight mass spectrometer (IM-MS/MS TOF) (LCQ Deca XP–Plus, Thermo Finnigan, San Jose, CA, USA). Data analysis was carried out as previously described [7,25].

### 2.11. Plasmid Construction

pCMV–Myc–VCP and pCMV–Myc–RCN2 constructs were generated by inserting cDNA encoding human VCP (Accession number: NM_007126.5) and RCN2 (Accession number: NM_002902.3) into the pCMV–Myc vector (631604; Clontech, Mountain View, CA, USA). pLKO.1–sh*RCN2* was constructed by inserting target gene into the pLKO.1 plasmid (8453; Addgene, Watertown, MA, USA). Mock (control shRNA) was constructed using the pLKO.1 backbone vector. Table 3 summarizes the primer sequences used for plasmid construction.

### 2.12. Lentivirus Production

Using human embryonic kidney 293T cells (CRL-3216; ATCC), lentiviruses encoding shRNA were created using the same methodology as a prior work [26].

### 2.13. In Vivo Pull-Down Assay

pCMV–Myc–VCP or pCMV–Myc–RCN2 plasmids were used to transfect HEK293T cells (CRL–11268; ATCC). Puromycin (BML–GR312–0050; Enzo Lifescience, Farmingdale, NY, USA) at a concentration of 1 μM was used to select transformed cells for 14 days. Every two days, the medium was replaced during the screening procedure. Following screening, cells were exposed to 4 μM biotinylated KB3409 for 4 days. A protease inhibitor cocktail (04693116001; Roche, Basel, Switzerland), 50 mM Tris–HCl (pH 7.4), 150 mM KCl, 1 mM PMSF, 2 mM benzamidine, and 0.05% NP–40 were used to lyse the cells. AccuNanoBeadTM streptavidin magnetic nanobeads (TA–1015–1; Bioneer, Daejeon, Republic of Korea) were then used for precipitation.

### 2.14. Western Blot Analysis

Western blot analysis was performed under the condition presented in Table 4. Proteins were detected using SuperSignal™ West Pico chemiluminescent substrate (34577; Thermo Fisher Scientific) and visualized with a ChemiDoc XRS+ system (1708265; Bio-Rad, Hercules, CA, USA).

### 2.15. In Silico Docking Studies

Ligand Generation and Optimization: The 2D and 3D structures of KB3409 and biotinylated KB3409 were generated using ChemDraw and Schrödinger Maestro program (version 14.6.126, MMshare Version 7.2.126, Release 2025-4, New York, NY, USA), respectively. The 3D structure of the ligands was generated by ionizing at pH (7.4 ± 2.0) using Epik and using OPLS4 force field, adding hydrogen atoms, and removing salts using the LigPrep module in the Maestro Builder panel.

Protein Preparation: The VCP structure (PDB ID: 3CF1) was obtained from the Protein Data Bank (https://www.rcsb.org) [27]. The protein structure was prepared using the protein preparation workflow in the Maestro panel. Hydrogen atoms were added and binding arrays were established throughout the protein generation process. Missing loops and side chains were filled using the Prime module. The water molecules were removed. The receptor grid box was generated using the “Generate Receptor Grid” module in Glide. The grid box was set to have its center at coordinates X: 94.87, Y: 49.55, Z: 289.99, and the length of the box was set to 20 Å each of X, Y, and Z. Docking study: In silico docking studies of KB3409 and biotinylated KB3409 with VCP protein were conducted using the Standard Precision and Extra Precision mode ligand docking protocol on the Maestro panel. The best docked poses of KB3409 and the biotinylated KB3409 were selected and conducted MM-GBSA and induced fit docking. The best poses of the ligand–protein complex were selected for the Molecular Dynamics simulation. Molecular Dynamics were conducted with TIP3P as solvent model, OPLS4 as a force field, sodium/chloride ions were added to neutralize. Molecular Dynamics simulation was performed for 100 ns at 300 K temperature and 1.01325 bar pressure.

### 2.16. ATPase Assay

The 6×His-tagged VCP protein was purified using the MagListo™ His-tagged Protein Purification Kit (K-7200; Bioneer). ATPase activity of VCP was determined spectrophotometrically by free inorganic phosphate in solution with Malachite Green Phosphate Detection Kit (12776; Cell Signaling Technology). Reaction buffer was composed of 50 mM Tris pH 7.4, 20 mM MgCl_2_, 1 mM EDTA, 0.5 mM DTT, and 0.01% Triton X-100. 50 nM purified VCP in a reaction buffer was incubated with DMSO, KB3409 and UP-163 (591242-60-3; MedChemExpress, Monmouth Junction, NJ, USA) for 30 min at 25 °C. 200 μM ATP (A6419-1G; Sigma) was added in a total of 25 μL VCP in reaction buffer for 10 min at 25 °C. Following the manufacturer’s instructions, 25 μL of the reaction mixture was combined with 100 μL of Malachite green reagent, and absorbance was measured at 630 nm in 5 min. At least three independent repetitions of each reaction were conducted.

### 2.17. Immunofluorescence

The immunofluorescence sample was made in accordance with earlier procedures [22]. The immunofluorescence was conducted under the conditions presented in Table 5.

### 2.18. Mitochondrial Calcium Intensity and Mitochondrial Permeability Transition Pore (mPTP) Assay

Calcium concentration and mitochondrial permeable transition pore (mPTP) opening in senescent fibroblasts were evaluated using the MitoProbe Transition Pore Assay Kit (M34153; Thermo Fisher Scientific) according to the manufacturer’s instructions. Cells were incubated at 37 °C for 15 min with or without 2 µM calcein-acetoxymethyl ester (AM), 80 µM CoCl_2_, and 0.1 µM ionomycin. Then, cells were washed with Hank’s buffered salt solution containing calcium (HBSS, 14025-092; Thermo Fisher Scientific). Subsequently, samples were analyzed using a flow cytometer with 495 nm excitation and 519 nm emission filters. The change in fluorescence intensity between samples cultured with only calcein AM/CoCl_2_ MFI and samples cultured with calcein AM/CoCl2/ionomycin MFI was considered to be due to mPTP opening. The (calcein-AM/CoCl_2_ MFI-pre-staining MFI) value represents mitochondrial calcium intensity. To inhibit mPTP opening, 1 µM cyclosporin A (C-6000; LC Laboratories, Woburn, MA, USA) was used.

### 2.19. Measurement of Oxygen Consumption Rate (OCR) and Extracellular Acidification Rate (ECAR)

Briefly, 1 × 10^4^ cells were placed in each well of an XFe96 cell culture plate from XFe96 FluxPak (103793-100; Seahorse Bioscience, Billerica, MA, USA) and cultured for 16 h in a 37 °C, 5% CO_2_ incubator. Afterward, the medium was replaced with glucose-free XF Assay medium (102365-100; Seahorse Bioscience) and cultured for 1 h in the same incubator. Then, as directed by the manufacturer, the Seahorse XF Cell Mito Stress Test Kit (101706-100; Seahorse Bioscience) was used to detect OCR, and the Seahorse XF Glycolytic Rate Assay Kit (103344–100; Seahorse Bioscience) was used to test ECAR. A Seahorse XFe96 Analyzer (Seahorse Bioscience) was used for all measurements.

### 2.20. Quantitative Polymerase Chain Reaction (qPCR)

qPCR was performed using a SolgTM 2× real-time PCR smart mix (SRH83–M40h; Solgent, Seoul, Republic of Korea) on a CFX ConnectTM Real-Time PCR Detection System (Bio-Rad). The following primer was used for qPCR (Table 6).

### 2.21. Measurement of the Effects of KB3409 on Young Fibroblasts

ROS levels and mitochondrial mass were measured in young fibroblasts treated with DMSO (0.01%) or KB3409 (1 μM) for 12 days at 4-day intervals. Then, cells were treated in media containing each dye for 30 min at 37 °C in order to assess ROS and mitochondrial mass (Table 2). A CytoFLEX flow cytometer (Beckman Coulter Life Sciences) was used to examine the cells.

Cell proliferation was evaluated in young fibroblasts treated with DMSO (0.01%) or KB3409 (1 μM) for 12 days at 4-day intervals. Cell proliferation was assessed using the EZ-Cytox reagent (EZ-500; DoGenBio), a water-soluble tetrazolium-based assay, according to the manufacturer’s instructions.

### 2.22. Statistical Analysis

Statistical analyses were performed using a standard statistical software package (SigmaPlot 12.5; Systat Software, San Jose, CA, USA). Student’s *t*-test, one-way ANOVA, and two-way ANOVA followed by Bonferroni’s post-test were used to determine whether differences were significant.

## 3. Results

### 3.1. High Throughput Screening for Compounds That Lower ROS Levels in Senescent Fibroblasts

**Figure 1:**
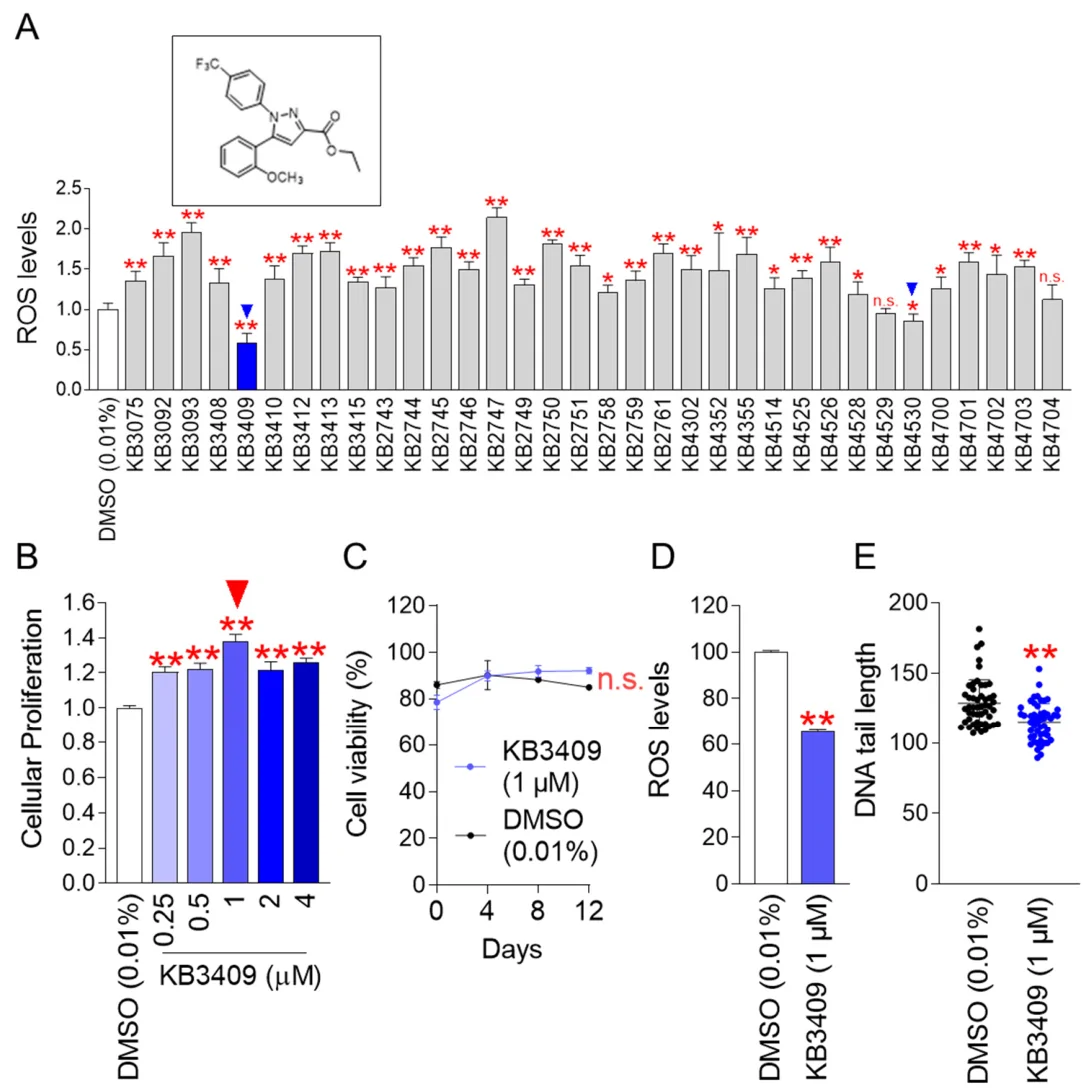
High throughput screening for compounds that lower ROS levels in senescent fibroblasts. (**A**) A quantitative assessment of ROS levels was conducted utilizing a DHR123-based method. Statistical analysis was performed using Bonferroni post hoc tests following one-way ANOVA. n.s. indicates not significant. * *p* < 0.05 and ** *p* < 0.01 were considered significant. Mean ± standard deviation, *n* = 10. Arrowhead indicates the compound that significantly reduced ROS levels compared to the DMSO control. Chemical structure of a pyrazole analog (KB3409) was shown in rectangle. Detailed data are provided in Table S2. (**B**) Cell proliferation was evaluated in senescent fibroblasts treated with DMSO (0.01%) or KB3409 (1 μM) for 12 days at 4-day intervals. Arrowhead indicates the selected KB3409 concentration. Statistical analysis was performed using Bonferroni post hoc tests following one-way ANOVA. ** *p* < 0.01 was considered significant. Mean ± standard deviation, *n* = 6. (**C**) Cell viability was evaluated after 0, 4, 8, and 12 days of DMSO (0.01%) or KB3409 (1 μM) treatment. Statistical analysis was performed using Bonferroni post hoc tests following two-way ANOVA. n.s. indicates not significant. Mean ± standard deviation, *n* = 3. (**D**) ROS levels were measured in senescent fibroblasts treated with DMSO (0.01%) or KB3409 (1 μM) for 12 days at 4-day intervals. DHR123 was used. For statistical analysis, Student’s *t*-test was used. ** *p* < 0.01 was considered significant. Mean ± standard deviation, *n* = 3. (**E**) DNA tail lengths were measured in senescent fibroblasts treated with DMSO (0.01%) or KB3409 (1 μM) for 12 days at 4-day intervals. For DNA tail length analysis, comet assay was used. For statistical analysis, Student’s *t*-test was used. ** *p* < 0.01 was considered significant. Mean ± standard deviation, *n* = 50.

This study aimed to discover candidate compounds exhibiting antioxidant activity through ROS-based screening. Senescent fibroblasts were treated with a library of oxazole and pyrazole analogs at a final concentration of 4 μM, and ROS levels were measured on day 12. Among the 35 candidate substances, KB3409 and KB4530 were found to significantly reduce ROS levels compared to the DMSO control group (Figure 1A; blue arrow heads, Table S2). Since KB3409 reduced ROS levels more than KB4530, KB3409 was selected for further analysis (Figure 1A). The chemical structure of KB3409, which includes the pyrazole moiety, is presented in Figure 1A.

As KB3409 was found to be effective in reducing ROS, the optimal concentration of KB3409 to reverse cellular senescence was investigated. Since cell cycle arrest is a characteristic of senescence [28], the concentration of KB3409 that promotes cell proliferation was investigated. Senescent fibroblasts were treated with KB3409 at concentrations of 0.25, 0.5, 1, 2, and 4 μM for 12 days. KB3409 at concentrations of 0.25, 0.5, 1, 2, and 4 μM significantly increased cell proliferation compared to the DMSO control group (Figure 1B). Although all tested concentrations of KB3409 significantly increased cell proliferation compared to the DMSO control, a clear dose-dependent effect was not observed. Notably, 1 μM induced the greatest proliferative response, while higher concentrations did not confer additional benefit. Therefore, 1 μM was selected as the minimal concentration achieving near-maximal efficacy and used for following experiments.

To evaluate the suitability of the selected concentrations, cell viability was analyzed to confirm the potential cytotoxicity of KB3409. The viability of senescent fibroblasts treated with 1 μM KB3409 was similar to that of the DMSO-treated control group, indicating that no distinct cytotoxicity was observed at that concentration (Figure 1C).

The concentration used for ROS-based screening (4 μM) differed from the finally selected concentration (1 μM). Thus, we confirmed whether the ROS-reducing effect was maintained even at a concentration of 1 μM KB3409. Compared to the DMSO control group, 1 μM KB3409 significantly reduced the ROS levels in senescent fibroblasts, demonstrating that KB3409 has the ability to continuously reduce ROS even at a concentration of 1 μM (Figure 1D).

Considering that ROS can directly cause DNA damage or inhibit the function of proteins involved in DNA maintenance [29], we further evaluated the DNA damage-related effects of KB3409. DNA damage was analyzed using DNA tail length, an indicator of DNA double-strand breaks (DSB). When senescent fibroblasts were treated with 1 μM KB3409, DNA tail length was reduced compared to the DMSO control group (Figure 1E). Since DNA is directly damaged by ROS, the reduction in damage confirms that KB3409 reduced ROS.

Pyrazole derivatives are known to exhibit intrinsic fluorescence owing to their favorable photophysical properties, including strong luminescence and membrane permeability [30]. Consistent with these properties, the pyrazole-based compound KB3409 exhibited intrinsic fluorescence, with maximum excitation and emission wavelengths of 370 and 410 nm, respectively (Figure S12). These wavelengths are sufficiently separated from those used for fluorescein isothiocyanate (FITC; excitation, 495 nm; emission, 519 nm) and phycoerythrin (PE; excitation, 565 nm; emission, 578 nm). Therefore, KB3409-derived fluorescence is not expected to substantially interfere with subsequent flow cytometric analyses performed using the FITC channel for ROS, MMP, and autophagic flux, or the PE channel for mitochondrial mass and MMP.

### 3.2. KB3409 Interacts with VCP and RCN2

**Figure 2:**
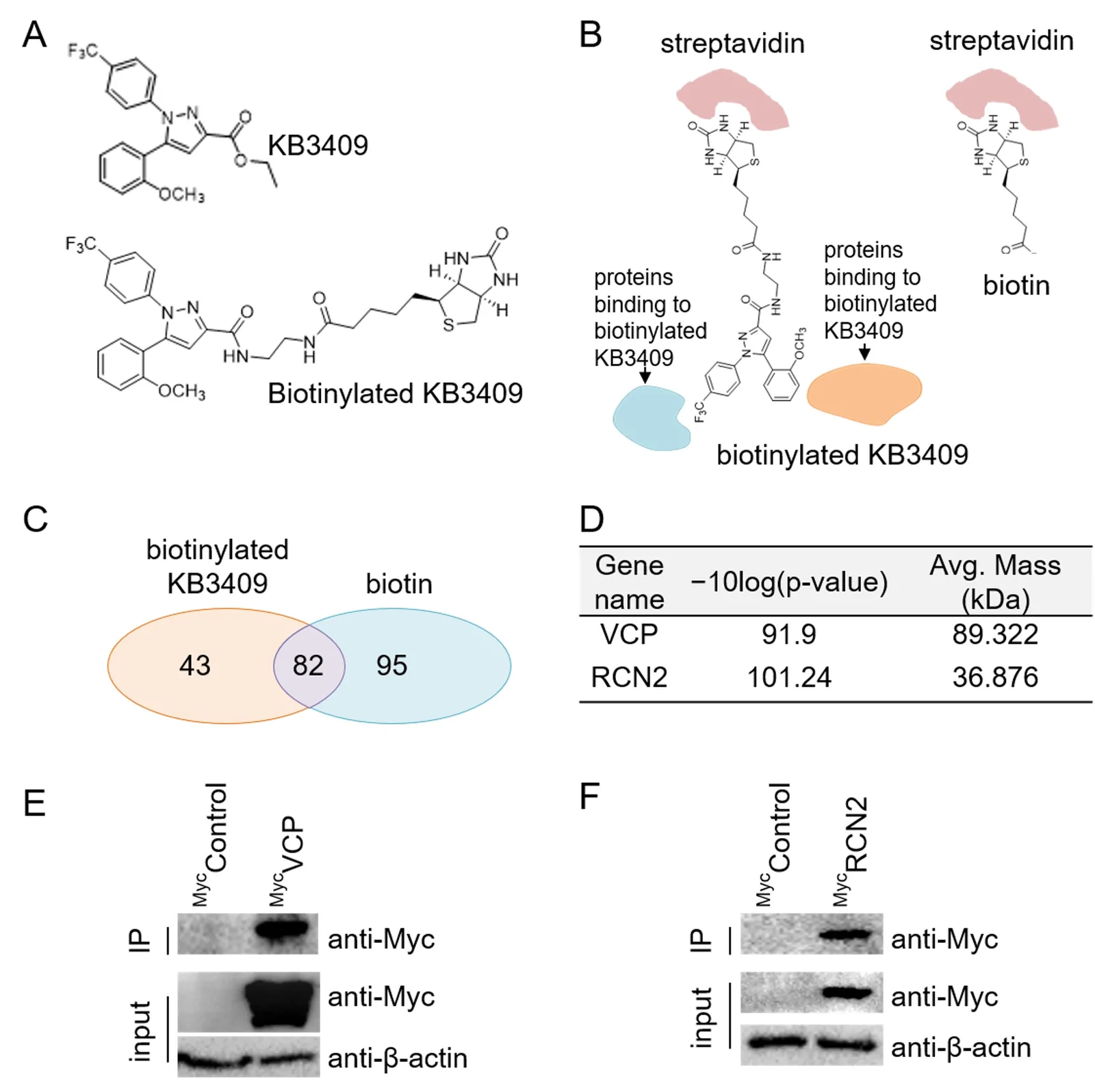
KB3409 interacts with VCP and RCN2. (**A**) Chemical structures of KB3409 and biotinylated KB3409. (**B**) Schematic diagram of the immunoprecipitation process using biotin or biotinylated KB3409. (**C**) Proteins binding to biotin or biotinylated KB3409 were identified using ion trap mass spectrometry. The number of proteins binding to biotinylated KB3409 and the number of proteins binding to biotin are shown in a Venn diagram. Detailed descriptions of the proteins are presented in Tables S3–S5. (**D**) Selected candidate proteins. −10logP: −10log (*p*-value), mean mass (kDa). (**E**,**F**) Co-precipitation of biotinylated KB3409 and Myc-tagged candidate proteins (Myc-tagged RCN2, Myc-tagged VCP). Myc-tag and β-actin antibodies were used in the Western blot. Figure S15 shows the full-size Western blot image.

The discovery that KB3409 effectively reduces ROS levels has sparked research into its underlying mechanisms. Proteins interacting with KB3409 can provide important insights into how this compound mediates ROS reduction. To identify these interacting partners, a pull-down analysis was performed using biotinylated KB3409 as a bait (Figure 2A,B). Biotinylated KB3409 was captured by streptavidin, enabling the co-precipitation of interacting proteins (Figure 2B). A pull-down was performed using biotin alone as a control (Figure 2B). The precipitated proteins were analyzed by ion trap mass spectrometry. A total of 43 proteins were specifically detected only under the biotinylated KB3409 condition, 95 proteins were specifically detected in the biotin control, and 82 proteins were identified under both conditions (Figure 2C; Tables S3–S5).

**Figure 3:**
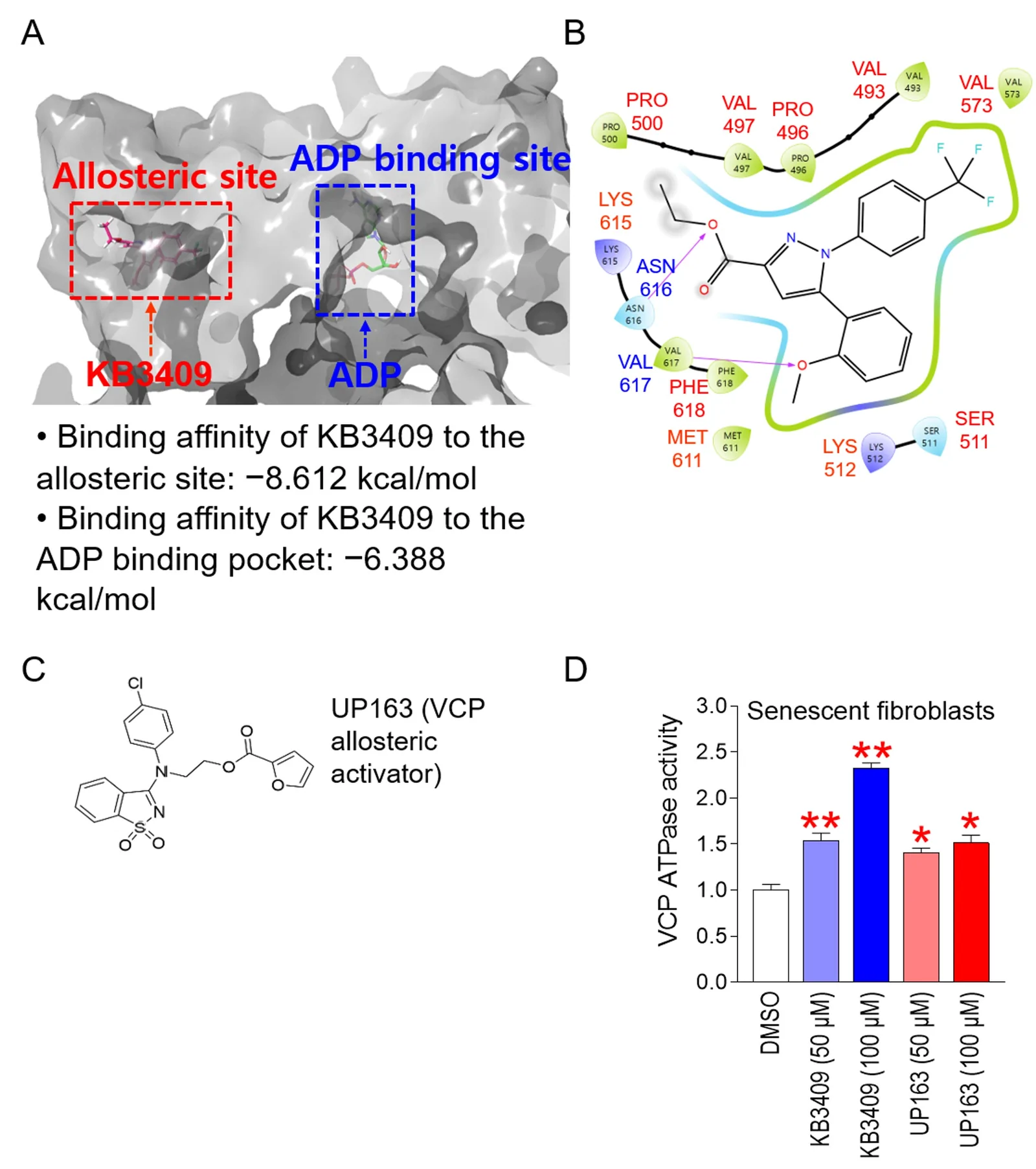
KB3409 functions as an allosteric activator of VCP. (**A**) Binding mode of KB3409 in VCP (PDB ID: 3CF1). Red dotted lines indicate allosteric site of VCP. Blue dotted lines indicate the ADP binding site. ADP anchored within an ADP binding site of VCP. (**B**) 2D interaction analysis revealed the detailed interaction analysis of KB3409 within the allosteric pocket. Purple lines indicate hydrogen-bonding interactions of KB3409 with ASN616 and VAL617. (**C**) Chemical structure of UP163, a reported VCP activator. (**D**) Colorimetric assay for protein phosphatase activity using malachite green phosphate detection kit. His-tagged purified VCP protein was treated with DMSO (0.01%), KB3409 (50, 100 μM) or UP163 (50, 100 μM). Statistical analysis was performed using Bonferroni post hoc tests following one-way ANOVA; findings were considered significant at * *p* < 0.05, ** *p* < 0.01. Mean ± standard deviation, *n* = 3.

Two selection criteria were applied to prioritize candidate proteins potentially involved in antioxidant effects. First, statistical reliability was evaluated by converting the *p*-value to −10log (*p*-value). A higher value indicates a lower probability of random association (Figure 2D; Table S3). Second, functional association with the antioxidant process was considered. Based on these criteria, RCN2 and VCP were selected as candidates for KB3409 interacting proteins. Both proteins exhibit high −10log (*p*-value) scores and are functionally associated with the regulation of oxidative stress (Figure 3D). RCN2 is an endoplasmic reticulum resident protein containing six EF-hand domains that regulate calcium homeostasis [18]. Given that endoplasmic reticulum Ca^2+^ dynamics influence mitochondrial calcium regulation, this interaction may be mechanically associated with the regulation of reactive oxygen species (ROS) generation [17]. VCP is an ATPase involved in various cellular processes, including protein homeostasis and organelle quality control [31]. In particular, VCP plays a crucial role in maintaining mitochondrial homeostasis through the regulation of mitochondrial autophagy, which suggests a potential association with ROS regulation [32].

The interactions between KB3409 and these candidate proteins were further validated through in vivo pull-down analysis. HEK293T cells were transfected with either pCMV–Myc–RCN2 or pCMV–Myc–VCP plasmids and cultured in the presence of biotinylated KB3409. After precipitating cell lysates with streptavidin, immunoblotting with anti-Myc antibodies confirmed that biotinylated KB3409 directly binds to both RCN2 and VCP (Figure 2E,F).

Following confirmation of the interactions of KB3409 with RCN2 and VCP, we analyzed the effects of KB3409 treatment on the expression levels of these two genes. In senescent fibroblasts, KB3409 treatment significantly reduced *RCN2* mRNA expression compared with the DMSO control treatment (Figure S13A). This finding prompted us to further investigate the effect of KB3409 on RCN2 protein levels. Consistent with the gene-expression results, KB3409 treatment markedly reduced RCN2 protein expression compared with the DMSO control treatment (Figures S13B and S14A).

In contrast, KB3409 treatment significantly increased *VCP* mRNA expression in senescent fibroblasts compared with the DMSO control treatment (Figure S13C). Moreover, KB3409 treatment slightly increased VCP protein levels compared with the DMSO control treatment (Figures S13D and S14B).

### 3.3. KB3409 Functions as an Allosteric Activator of VCP

To predict the potential binding modes of KB3409 with VCP (PDB ID: 3CF1) [27], we performed in silico molecular docking and molecular dynamics simulations. Two potential binding sites were identified: an allosteric site (Figure 3A, red box) and the canonical ADP-binding site (Figure 3A, blue box). The molecular docking scores suggested that the allosteric site is the primary target of KB3409, yielding a significantly higher binding affinity compared to the ADP-binding pocket (−8.612 kcal/mol vs. −6.388 kcal/mol, respectively) (Figure 3A).

Detailed interaction analysis of KB3409 within the allosteric pocket revealed that KB3409 is surrounded by VAL493, PRO496, VAL497, PRO500, SER511, LYS512, VAL573, MET611, LYS615, ASN616, VAL617, and PHE618 within a radius of 2.5 Å (Figure 3B). Additionally, KB3409 formed hydrogen bond interactions with ASN616 and VAL617 (Figure 3B).

Based on these structural insights, we hypothesized that KB3409 functions as an allosteric activator of VCP. To experimentally verify this hypothesis, the effect of KB3409 on VCP ATPase activity was evaluated. UP163, a known VCP allosteric activator, was used as a positive control [33] (Figure 3C).

Notably, treatment with KB3409 significantly elevated VCP ATPase activity compared to the DMSO vehicle control (Figure 3D). The magnitude of this enzymatic enhancement was comparable to that induced by the positive control, UP163 (Figure 3D). Taken together, these biochemical and computational studies strongly suggest that KB3409 directly accelerates VCP ATPase activity through an allosteric mechanism of action.

### 3.4. KB3409 Promotes Mitochondrial Quality Control by Enhancing VCP-Associated Autophagic Flux

**Figure 4:**
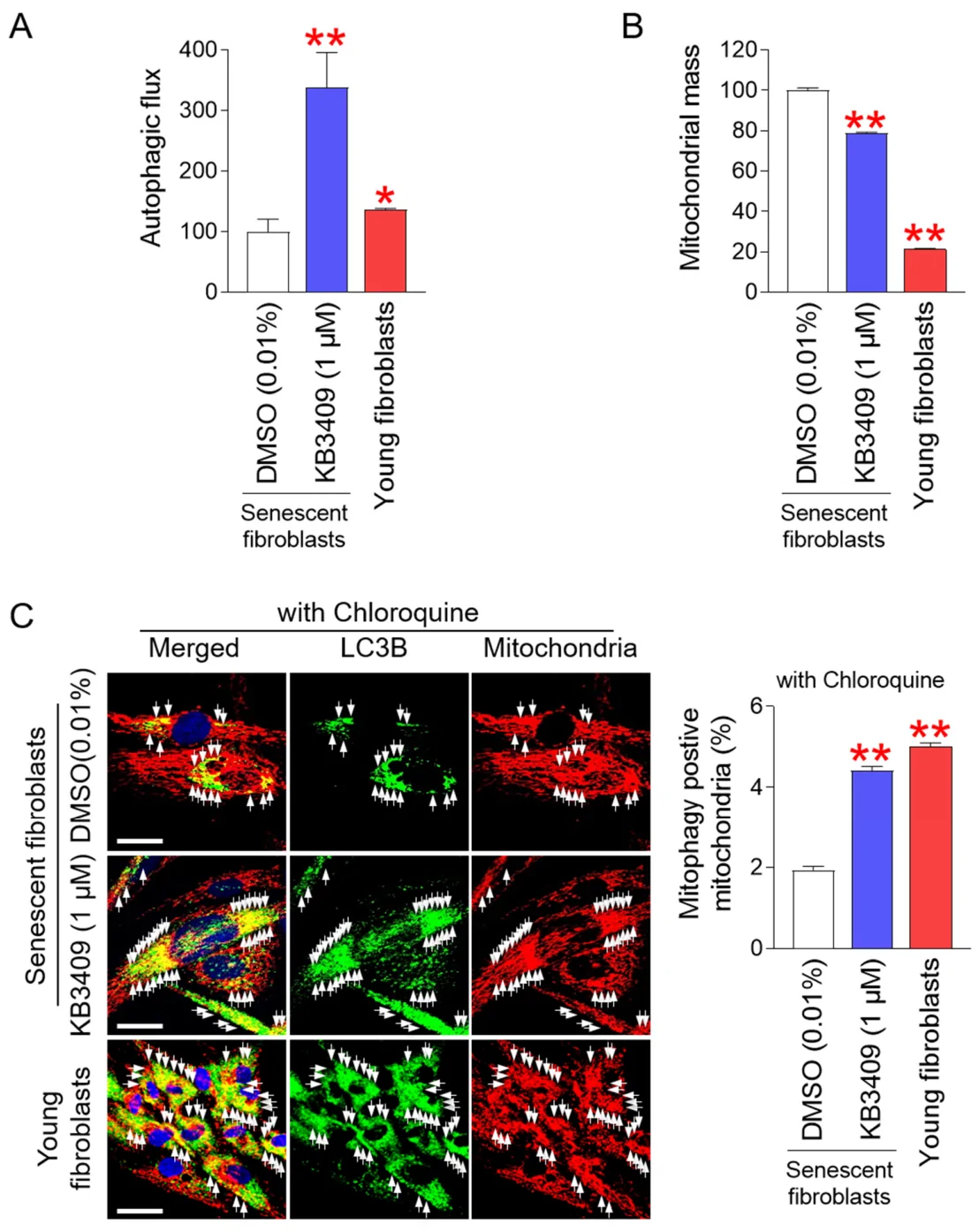
KB3409 promotes mitochondrial quality control by enhancing VCP-associated autophagic flux. (**A**,**B**) Autophagy flux and mitochondrial mass were measured in senescent fibroblasts administered with DMSO (0.01%) or KB3409 (1 μM) at 4-day intervals for 12 days. The positive control was young fibroblasts. Statistical analysis was performed using Bonferroni post hoc tests following one-way ANOVA. * *p* < 0.05 and ** *p* < 0.01 were considered significant. Mean ± standard deviation, *n* = 3. (**C**) Senescent fibroblasts were administered with DMSO (0.01%) or KB3409 (1 μM) at 4-day intervals for 12 days. Senescent fibroblasts were co-treated with 20 μM chloroquine 24 h prior to immunostaining. Subsequently, LC3B (green) and mitochondria (red) were immunostained. Nuclei were stained using Hoechst 33342 (blue). The positive control was young fibroblasts. The scale bar represents 10 μm. Mitophagy is indicated by white arrows. For statistical analysis, Bonferroni post hoc tests were used following one-way ANOVA, and ** *p* < 0.01 value was deemed statistically significant. Mean ± standard deviation, *n* = 3. Full-size immunofluorescence images are shown in Figure S20.

VCP plays a crucial role in maintaining protein homeostasis by regulating the maturation of the autophagy process and promoting the removal of dysfunctional proteins and organelles, including mitochondria [32]. Considering that KB3409 increases VCP ATPase activity, we investigated the effect of KB3409 on autophagy activity by measuring autophagy flux. The positive control was young fibroblasts. Young fibroblasts showed a significant increase in autophagy flux when compared with DMSO-treated senescent fibroblasts (Figure 4A). Moreover, KB3409 significantly increased autophagy flux in senescent fibroblasts compared to DMSO-treated controls, suggesting an enhancement of the autophagy process (Figure 4A).

To determine if this effect also applies to mitochondrial turnover, we measured mitochondrial mass The positive control was young fibroblasts. Young fibroblasts demonstrated a significant decrease in mitochondrial mass compared to DMSO-treated senescent fibroblasts (Figure 4B). Treatment with KB3409 significantly reduced mitochondrial mass in senescent fibroblasts, indicating increased mitochondrial removal consistent with the activation of autophagy activity (Figure 4B).

Mitophagy maintains mitochondrial integrity by selectively removing damaged mitochondria [34]. To evaluate the translocation of mitochondria to autophagosomes in more detail, we analyzed the colocation of microtubule-associated protein 1 light chain 3 beta (LC3B) and mitochondria, which are indicators of autophagosome formation. KB3409 significantly increased the mitochondrial-LC3B colocation compared to the DMSO-treated control group, indicating enhanced mitochondrial influx into the autophagy pathway (Figure S16). To investigate these observations further, senescent fibroblasts were treated with chloroquine (CQ), a lysosomal degradation inhibitor, to induce autophagosome accumulation [35]. The positive control was young fibroblasts. Young fibroblasts demonstrated a significant increase in the mitochondrial-LC3B colocation compared to CQ and DMSO-cotreated senescent fibroblasts (Figure 4C). Moreover, when CQ and KB3409 were co-treated, the mitochondrial-LC3B colocation significantly increased more than when CQ and DMSO were co-treated, suggesting enhanced mitochondrial delivery to autophagosomes (Figure 4C; white arrows).

An increase in LC3B-I levels may reflect enhanced LC3B synthesis, whereas LC3B-II is commonly used as a marker of autophagosome formation [36]. To further assess KB3409-mediated activation of mitophagy, we analyzed LC3B-I and LC3B-II levels by Western blotting. Compared with the DMSO control, KB3409 treatment increased the levels of both LC3B-I and LC3B-II, suggesting an increase in the cellular LC3B pool accompanied by autophagosome accumulation (Figure S17).

To further substantiate that KB3409 promotes mitophagy, we analyzed the colocation of mitochondria and PTEN induced kinase 1 (PINK1), which is molecular trigger for mitophagy [37]. KB3409 treatment significantly increased mitochondrial–PINK1 colocalization compared with the DMSO-treated control group, suggesting enhanced recruitment of PINK1 to mitochondria and activation of the PINK1-associated mitophagy pathway (Figures S18 and S19). These findings provide additional support for the notion that KB3409 promotes mitophagy.

Collectively, these findings suggest that KB3409 promotes mitochondrial quality control by enhancing VCP-associated autophagic flux, thereby facilitating the efficient clearance of damaged mitochondria.

### 3.5. KB3409 Decreases Mitochondrial Ca^2+^ Overload via RCN2

**Figure 5:**
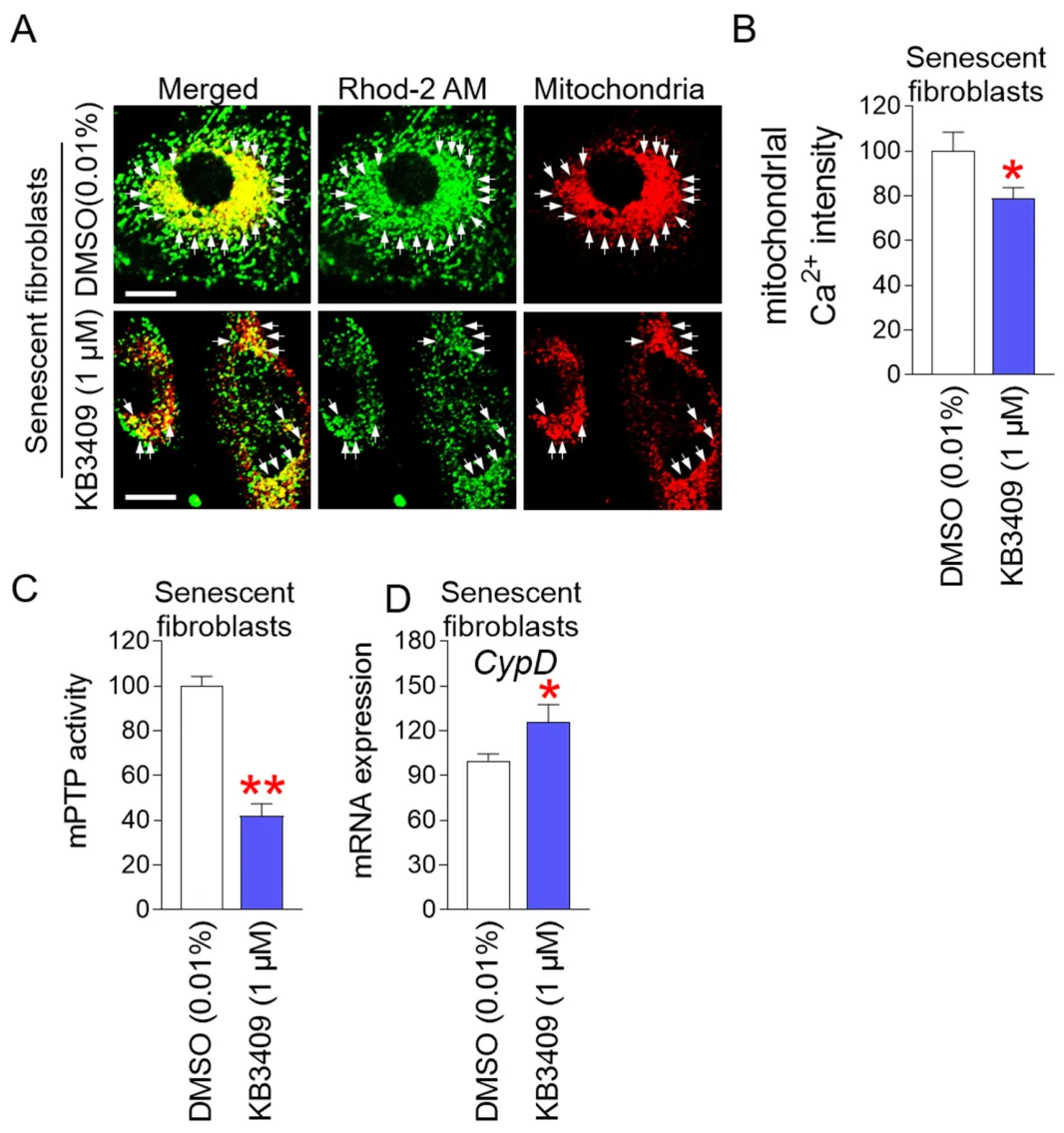
KB3409 decreases mitochondrial Ca^2+^ overload via RCN2. Senescent fibroblasts were administered DMSO (0.01%) or KB3409 (1 μM) at 4-day intervals for 12 days. (**A**) Rhod-2 AM (green) and mitochondrial (red) staining. The scale bar represents 10 μm. Co-locations of mitochondria and calcium ions (yellow) are indicated by white arrows. Figure S21 shows the full-size immunofluorescence image. (**B**) Senescent fibroblasts were treated with 80 μM CoCl_2_ and 2 μM calcein-acetoxymethyl ester (AM) to measure mitochondrial Ca^2+^ intensity. For statistical analysis, Student’s *t*-test was used, and * *p* < 0.05 was deemed statistically significant. Mean ± standard deviation, *n* = 3. (**C**) mPTP activity was measured under conditions with or without the addition of 2 μM calcein-acetoxymethyl ester (AM), 80 μM CoCl_2_, and 0.1 μM ionomycin. For statistical analysis, Student’s *t*-test was used, and ** *p* < 0.01 was deemed statistically significant. Mean ± standard deviation, *n* = 3. (**D**) *CypD* mRNA expression levels were evaluated in senescent fibroblasts administered with DMSO (0.01%) or KB3409 (1 μM) at 4-day intervals for 12 days. For statistical analysis, Student’s *t*-test was used, and * *p* < 0.05 was deemed statistically significant. Mean ± standard deviation, *n* = 3.

Mitochondrial Ca^2+^ homeostasis is an important determinant of mitochondrial function [38]. RCN2, an ER-resident Ca^2+^-binding protein, has been implicated in the regulation of Ca^2+^ transfer at ER–mitochondria contact sites and may contribute to mitochondrial Ca^2+^ overload by modulating inter-organelle Ca^2+^ flux [39]. We therefore hypothesized that KB3409 modulates mitochondrial Ca^2+^ homeostasis via RCN2-associated pathways. To test this, mitochondrial Ca^2+^ levels were assessed using Rhod-2 AM, a cationic fluorescent Ca^2+^ indicator, in combination with MitoTracker to enhance mitochondrial specificity. In senescent fibroblasts, KB3409 treatment reduced the co-localization of Rhod-2 AM with mitochondria compared to DMSO-treated controls, indicating decreased mitochondrial Ca^2+^ accumulation (Figure 5A). Consistently, KB3409 also reduced mitochondrial Ca^2+^ intensity in senescent fibroblasts, as measured using the calcein AM–CoCl_2_ assay (Figure 5B).

Excessive mitochondrial Ca^2+^ accumulation triggers opening of the mitochondrial permeability transition pore (mPTP), leading to a breakdown of mitochondrial function [40]. In line with the observed reduction in mitochondrial Ca^2+^, KB3409 significantly inhibited mPTP opening in senescent fibroblasts relative to DMSO controls (Figure 5C).

We further investigated the effect of KB3409 on the expression of cyclophilin D (CypD), a mitochondrial matrix protein involved in the regulation of mPTP opening [41]. CypD interacts with various protein complexes in the inner mitochondrial membrane and promotes mPTP opening by lowering its activation threshold in response to excessive mitochondrial Ca^2+^ accumulation [41]. KB3409 treatment significantly increased *CypD* mRNA expression in senescent fibroblasts compared to the DMSO control group (Figure 5D). This finding contrasted with the reduced mitochondrial Ca^2+^ accumulation and mPTP opening observed following KB3409 treatment (Figure 5A–C). One possible explanation is that the increase in *CypD* mRNA expression represents a compensatory homeostatic response to the KB3409-induced reductions in mitochondrial Ca^2+^ accumulation and mPTP opening. However, further investigation is required to clarify the precise relationship between *CypD* mRNA expression and mPTP activity in response to KB3409.

### 3.6. KB3409-Mediated Regulation of RCN2 Is Required for Reduction in Mitochondrial Ca^2+^ Overload

**Figure 6:**
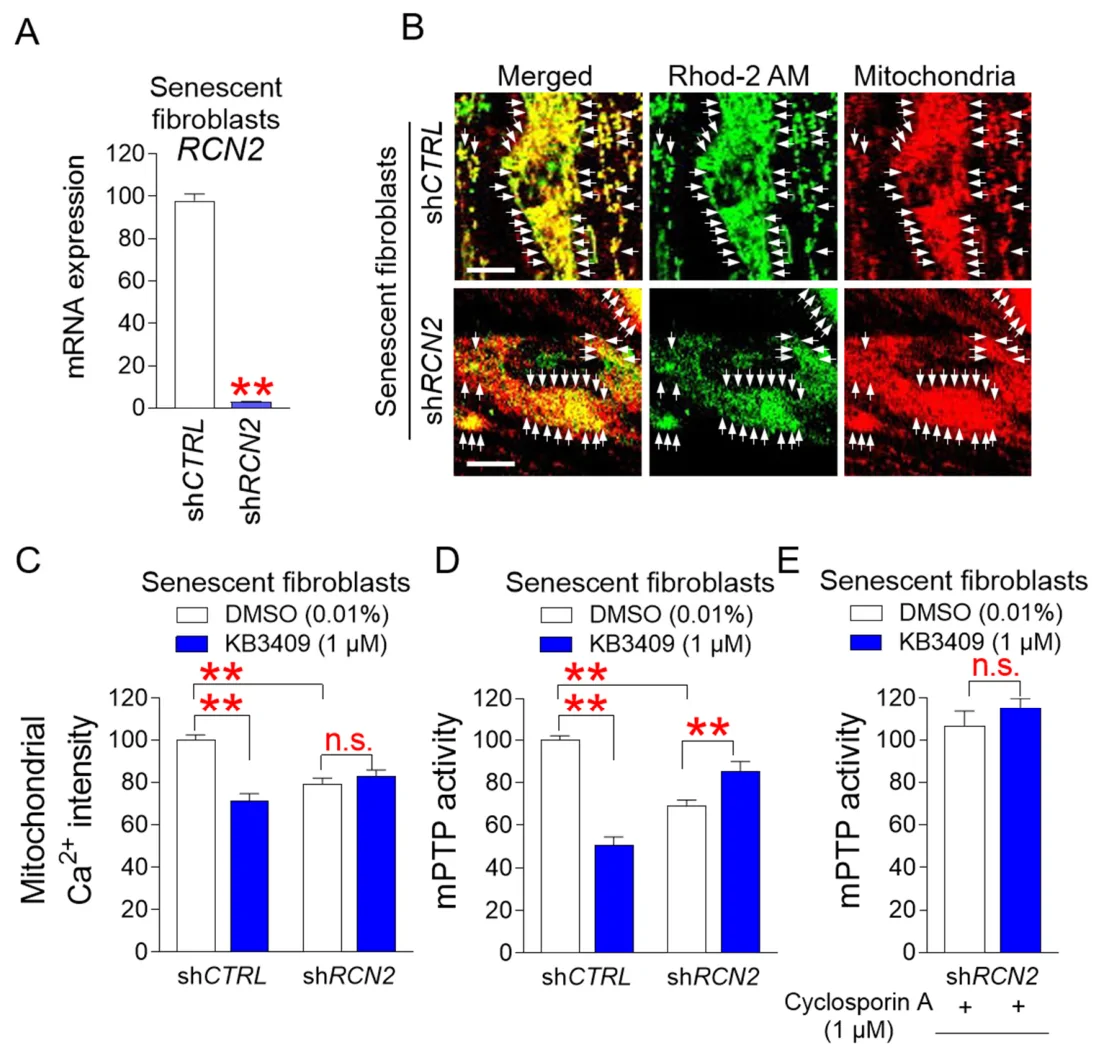
KB3409-mediated regulation of *RCN2* is required for reduction in mitochondrial Ca^2+^ overload. (**A**,**B**) Lentiviruses expressing sh*CTRL* or sh*RCN2* were transduced into senescent fibroblasts. *RCN2* mRNA expression levels (**A**). For statistical analysis, Student’s *t*-test was used, and ** *p* < 0.01 was deemed statistically significant. Mean ± standard deviation, *n* = 3. Rhod-2 AM (green) and mitochondrial (red) staining (**B**). The scale bar represents 10 μm. Co-locations of mitochondria and calcium ions are (yellow) indicated by white arrows. Figure S22 shows full-size immunofluorescence images. (**C**,**D**) Senescent fibroblasts transduced with lentiviruses expressing sh*CTRL* or sh*RCN2* were treated with DMSO (0.01%) or KB3409 (1 μM). mPTP activity and mitochondrial Ca^2+^ intensity were measured. Statistical analysis was performed using Bonferroni post hoc tests following one-way ANOVA. n.s. indicates not significant. ** *p* < 0.01 was considered significant. Mean ± standard deviation, *n* = 3. (**E**) Cyclosporin A/DMSO or cyclosporin A/KB3409 were co-treated to shR*CN2*-transduced senescent fibroblasts, and mPTP activity was evaluated. For statistical analysis, Student’s *t*-test was used, and the results were found to be non-significant (n.s.). Mean ± standard deviation, *n* = 3.

Given that KB3409 inhibits mitochondrial Ca^2+^ overload, we next investigated whether *RCN2* is required for this effect. To this end, endogenous *RCN2* expression was silenced using shRNA targeting RCN2 (sh*RCN2*). sh*RCN2* efficiently reduced *RCN2* expression in senescent fibroblasts compared to the shCTRL group (Figure 6A).

Mitochondrial Ca^2+^ levels were then assessed using Rhod-2 AM in combination with MitoTracker. In senescent fibroblasts, *RCN2* knockdown decreased the co-localization of Rhod-2 AM with mitochondria relative to sh*CTRL*-transduced senescent fibroblasts, indicating reduced mitochondrial Ca^2+^ accumulation (Figure 6B).

To determine whether RCN2 is required for the effects of KB3409, senescent fibroblasts expressing sh*CTRL* or sh*RCN2* were treated with DMSO or KB3409. In sh*CTRL*-transduced senescent fibroblasts, KB3409 significantly reduced mitochondrial Ca^2+^ levels compared to the DMSO control (Figure 6C). In contrast, this effect was abolished in *RCN2*-silenced senescent fibroblasts, where KB3409 failed to further decrease mitochondrial Ca^2+^ levels (Figure 6C).

Consistently, KB3409 significantly suppressed mPTP opening in sh*CTRL*-transduced senescent fibroblasts, whereas this effect was not observed in *RCN2*-silenced senescent fibroblasts (Figure 6D). Notably, KB3409 treatment increased mPTP activity in *RCN2*-silenced senescent fibroblasts, suggesting a context-dependent effect that warrants further investigation.

To determine whether the increase in mPTP activity observed in *RCN2*-deficient cells following KB3409 treatment represented a mechanistically relevant phenomenon rather than experimental variation, we performed a cotreatment experiment using cyclosporin A, an inhibitor of mPTP opening [42]. When *RCN2*-suppressed senescent fibroblasts were treated with cyclosporin A and KB3409 in combination, mPTP activity did not differ significantly from that observed following combined treatment with cyclosporin A and DMSO (Figure 6E). Accordingly, the increase in mPTP activity observed after KB3409 treatment alone in *RCN2*-deficient cells was no longer detected in the presence of cyclosporin A (Figure 6D,E).

Collectively, these results demonstrate that KB3409-mediated regulation of *RCN2* is required for reduction in mitochondrial Ca^2+^ overload and subsequent inhibition of mPTP opening.

### 3.7. KB3409 Enhances Mitochondrial Respiratory Capacity

**Figure 7:**
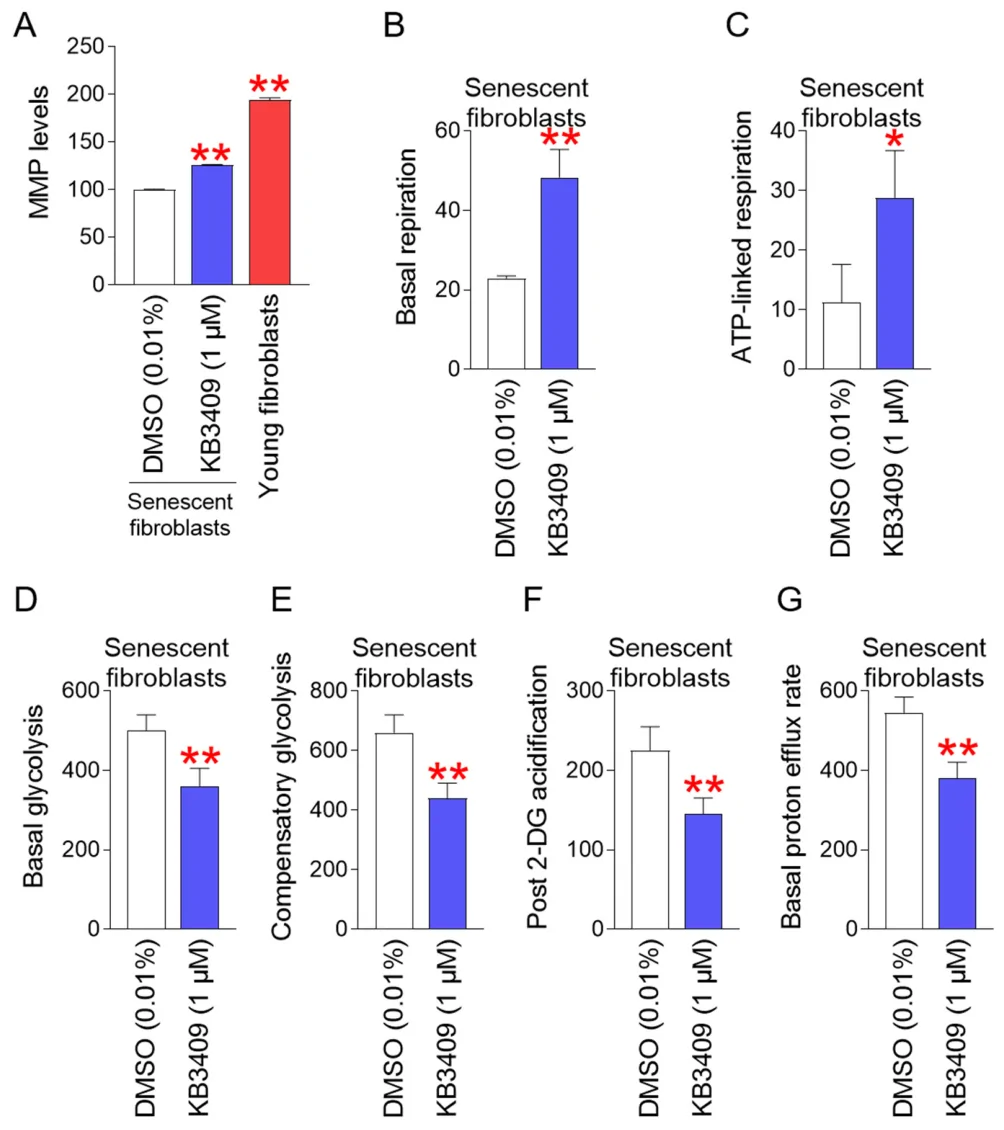
KB3409 enhances mitochondrial respiratory capacity. (**A**) Flow cytometric analysis was conducted to assess the levels of mitochondria membrane potential (MMP) staining with JC-10. The positive control was young fibroblasts. Statistical analysis was performed using Bonferroni post hoc tests following one-way ANOVA. ** *p* < 0.01 was considered significant. Mean ± standard deviation, *n* = 3. (**B**,**C**) OCR was used to measure basal respiration and ATP-linked respiration. For statistical analysis, Student’s *t*-test was used. * *p* < 0.05 and ** *p* < 0.01 were considered significant. Mean ± standard deviation, *n* = 3. (**D**–**G**) ECAR was used to measure basal glycolysis, compensatory glycolysis, post 2-DG acidification, and basal proton efflux rate. For statistical analysis, Student’s *t*-test was used. ** *p* < 0.01 was considered significant. Mean ± standard deviation, *n* = 3.

Opening of the mPTP leads to reduction in the mitochondrial membrane potential (MMP), the electrochemical gradient established by ETC-driven proton pumping across the inner mitochondrial membrane [43]. Considering that KB3409 reduces mPTP activity, we next investigated its effect on MMP. The positive control was young fibroblasts. Young fibroblasts showed a significant increase in MMP compared to DMSO-treated senescent fibroblasts (Figure 7A). Moreover, senescent fibroblasts treated with KB3409 showed a significant increase in MMP compared to the DMSO control group, indicating that the preservation of mitochondrial membrane integrity and electrochemical coupling (Figure 7A).

Since mitochondrial membrane potential (MMP) is an important determinant of oxidative phosphorylation (OXPHOS) capacity, mitochondrial respiratory function was further evaluated by measuring the oxygen consumption rate (OCR). Treatment with KB3409 significantly increased basal respiration compared with DMSO-treated senescent fibroblasts, indicating that KB3409 enhances mitochondrial respiratory activity (Figure 7B). In addition, KB3409 significantly increased ATP-linked respiration compared with DMSO treatment, suggesting that KB3409 improves the capacity of mitochondria to generate ATP through OXPHOS (Figure 7C).

To evaluate whether these alterations were accompanied by changes in cellular metabolic preference, the basal glycolysis, compensatory glycolysis, acidification after 2-DG treatment, and basal proton efflux rate were evaluated. In particular, the level of basal glycolysis in senescent fibroblasts treated with KB3409 was significantly lower than that of fibroblasts treated with DMSO, indicating that KB3409 reduced the rate at which glucose is converted to lactate [44] (Figure 7D). Compensatory glycolysis, which inhibits OXPHOS and induces a compensatory switch to glycolysis, was reduced in senescent fibroblasts treated with KB3409, indicating that KB3409 reduced maximum glycolytic capacity [44] (Figure 7E). Furthermore, senescent fibroblasts treated with KB3409 showed a significant reduction in acidification after 2-deoxy-D-glucose (2-DG) treatment, indicating that KB3409 reduces residual glycolysis, which 2-DG does not completely stop [44] (Figure 7F). Lactic acid is produced through glycolysis using protons that are not required for cellular respiration [45]. A small amount of energy is generated during the inefficient energy metabolism of lactic acid fermentation [45]. More protons are generated while pyruvate is converted to lactic acid [46]. Senescent fibroblasts treated with KB3409 had a significantly lower basal proton flow rate than cells treated with DMSO (Figure 7G). These results demonstrate that KB3409 slowed down the rate of glycolysis.

Collectively, these results support a model in which inhibition of mPTP opening by KB3409 maintains mitochondrial membrane potential (MMP), enhances mitochondrial respiratory capacity, and relatively reduces glycolytic activity in senescent fibroblasts.

### 3.8. KB3409 as a Potential Compound for Ameliorating Senescence-Associated Phenotypes

**Figure 8:**
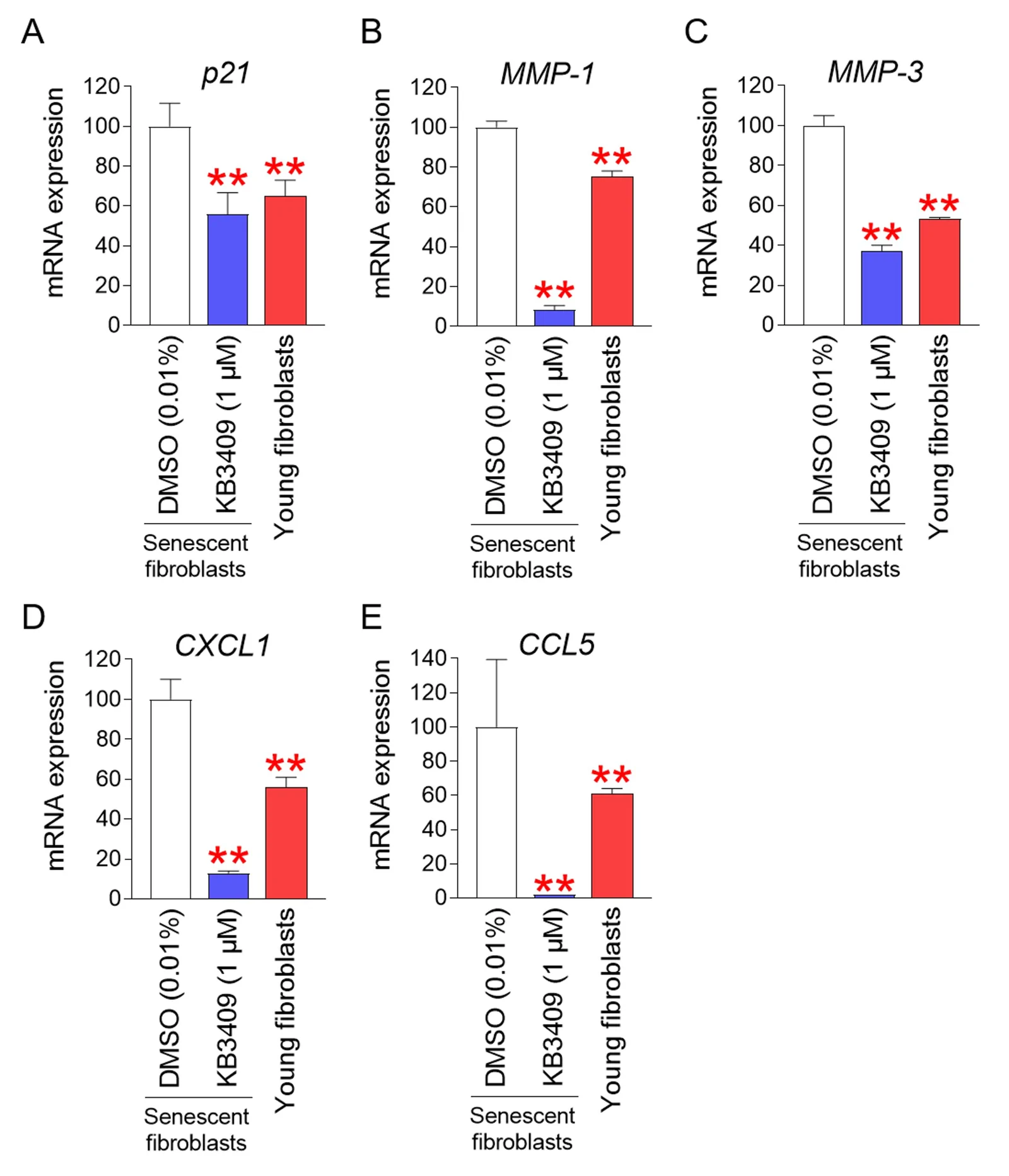
KB3409 as a potential compound for ameliorating senescence-associated phenotypes. mRNA expression levels of *p21* (**A**), *MMP-1* (**B**), *MMP-3* (**C**), *CXCL1* (**D**), and *CCL5* (**E**) were measured in senescent fibroblasts treated with DMSO (0.01%) or KB3409 (1 μM) for 12 days at 4-day intervals. The positive control was young fibroblasts. Statistical analysis was performed using Bonferroni post hoc tests following one-way ANOVA. ** *p* < 0.01 was considered significant. Mean ± standard deviation, *n* = 3.

Restoration of mitochondrial function is considered a key factor in the process of reversing senescence [47]. In senescent cells, increased transcriptional activation of p21 induces cell cycle arrest [47]. Accordingly, we evaluated the effect of KB3409 on *p21* mRNA expression. The positive control was young fibroblasts. Young fibroblasts showed a significant decrease in *p21* mRNA expression when compared to DMSO-treated senescent fibroblasts (Figure 8A). Moreover, *p21* mRNA expression in senescent fibroblasts treated with KB3409 was significantly reduced compared to the DMSO-treated group, suggesting a partial alleviation of cell cycle arrest (Figure 8A).

Persistent cell cycle arrest leads to the acquisition of senescence-associated phenotypes, including the senescence-associated secretory phenotype (SASP), which contributes to the active reconfiguration of the tissue microenvironment [48]. Among the components of SASPs, substrate metalloproteinases (MMPs) play a central role in the degradation of the extracellular matrix [49]. MMP-1 contributes to the structural deterioration of the extracellular matrix by directly degrading type 1 and type 3 collagen, whereas MMP-3 amplifies these effects by activating other members of the MMP family [50]. We then investigated the effects of KB3409 on *MMP-1* and *MMP-3* expression. The positive control was young fibroblasts. Young fibroblasts showed a significant decrease in the mRNA expression of *MMP-1* and *MMP-3* compared to DMSO-treated senescent fibroblasts (Figure 8B,C). Moreover, KB3409 treatment significantly reduced the mRNA expression of *MMP-1* and *MMP-3* in senescent fibroblasts compared to DMSO-treated controls, suggesting that KB3409 attenuates SASP-related deterioration of the extracellular matrix (Figure 8B,C).

Chemokine (C-X-C motif) ligand 1 (CXCL1) and chemokine (C-C motif) ligand 5 (CCL5) are major SASP components secreted by senescent cells that contribute to inflammatory signaling [51]. In parallel, we investigated the effects of KB3409 on *CXCL1* and *CCL5* expression. The positive control was young fibroblasts. Young fibroblasts demonstrated a significant decrease in the mRNA expression of *CXCL1* and *CCL5* compared to DMSO-treated senescent fibroblasts (Figure 8D,E). Moreover, KB3409 treatment significantly reduced *CXCL1* and *CCL5* mRNA expression in senescent fibroblasts compared to DMSO controls, suggesting that KB3409 also inhibits the inflammatory components of SASP (Figure 8D,E).

### 3.9. KB3409’s Effect Was Not Restricted to Senescent Fibroblasts

The finding that KB3409 improved senescence-associated phenotypes let us investigate whether its effects were selective for senescent fibroblasts or were also observed in young fibroblasts. Thus, we examined the effects of KB3409 on intracellular ROS levels and mitochondrial mass.

**Figure 9:**
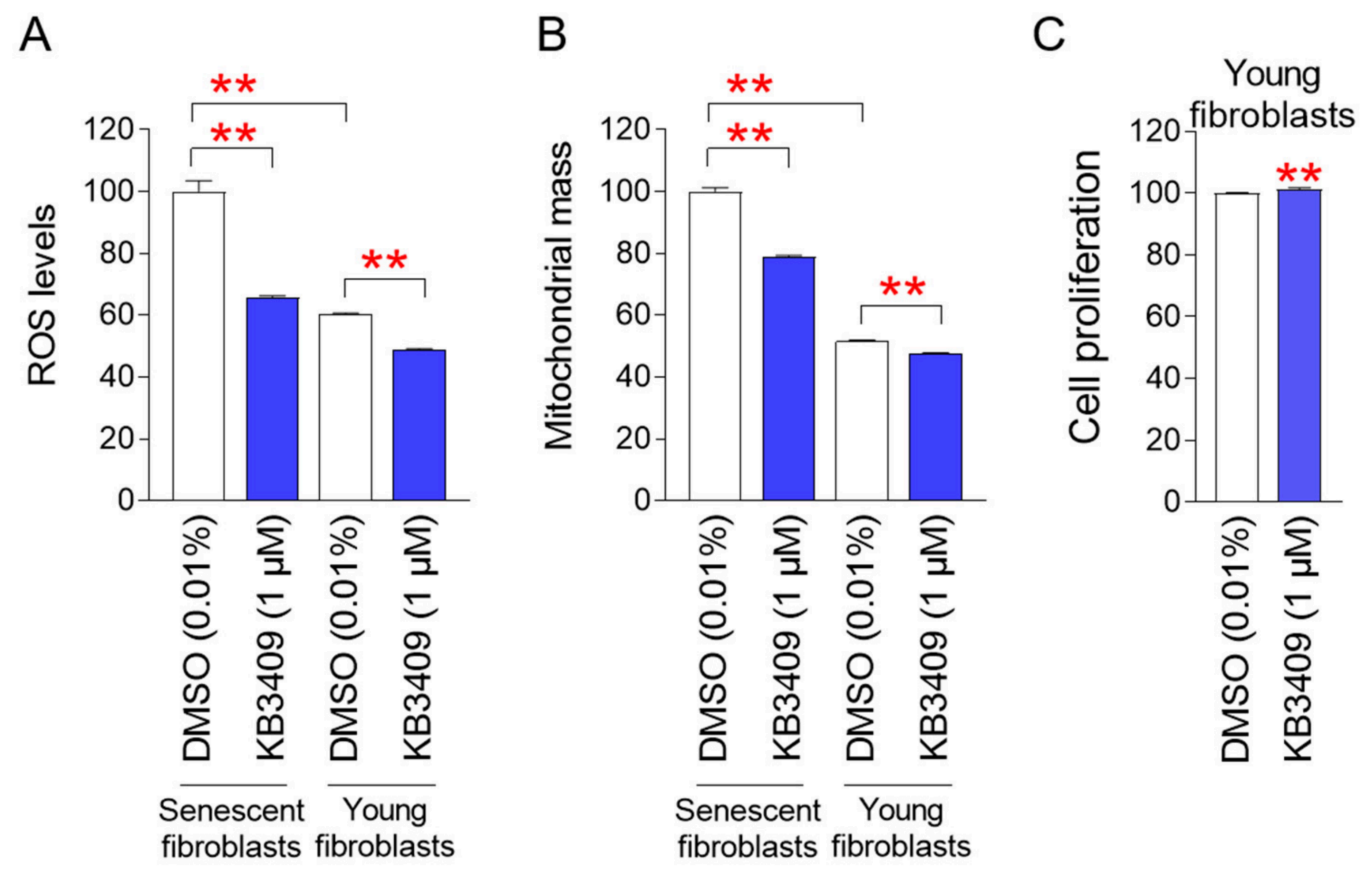
KB3409’s effect was not restricted to senescent fibroblasts. (**A**) ROS levels were measured in senescent and young fibroblasts treated with DMSO (0.01%) or KB3409 (1 μM) for 12 days at 4-day intervals. DHR123 was used. Statistical analysis was performed using Bonferroni post hoc tests following one-way ANOVA. ** *p* < 0.01 was considered significant. Mean ± standard deviation, *n* = 3. (**B**) Mitochondrial mass was measured in senescent and young fibroblasts treated with DMSO (0.01%) or KB3409 (1 μM) for 12 days at 4-day intervals. MitoTrackerTM Deep Red FM Dye was used. Statistical analysis was performed using Bonferroni post hoc tests following one-way ANOVA. ** *p* < 0.01 was considered significant. Mean ± standard deviation, *n* = 3. (**C**) Cell proliferation was evaluated in young fibroblasts treated with DMSO (0.01%) or KB3409 (1 μM) for 12 days at 4-day intervals. For statistical analysis, Student’s *t*-test was used. ** *p* < 0.01 was considered significant. Mean ± standard deviation, *n* = 6.

ROS levels were initially compared between young and senescent fibroblasts. Young fibroblasts exhibited significantly lower ROS levels than senescent fibroblasts, consistent with the increased ROS levels observed in senescent fibroblasts [52] (Figure 9A). In senescent fibroblasts, KB3409 treatment significantly decreased ROS levels compared with the DMSO-treated control group, thereby confirming its previously observed ROS-lowering effect (Figure 1D and Figure 9A). KB3409 also significantly decreased ROS levels in young fibroblasts compared to the corresponding DMSO control group (Figure 9A). These findings indicate that KB3409 reduces ROS levels in both senescent and young fibroblasts, suggesting that this effect is not restricted to senescent cells.

Mitochondrial mass was significantly higher in senescent than in young fibroblasts, consistent with the accumulation of damaged or dysfunctional mitochondria during cellular senescence [53] (Figure 4B and Figure 9B). In senescent fibroblasts, KB3409 treatment significantly decreased mitochondrial mass compared with the DMSO-treated control group, thereby confirming its previously observed mitochondrial mass-reducing effect (Figure 4B and Figure 9B). KB3409 also significantly decreased mitochondrial mass in young fibroblasts compared with the corresponding DMSO control group (Figure 9B). These findings indicate that KB3409 reduces mitochondrial mass in both senescent and young fibroblasts, suggesting that this effect is not restricted to senescent cells.

Next, we investigated whether KB3409 promotes cell proliferation in young fibroblasts as well. In young fibroblasts, KB3409 significantly increased cell proliferation compared with the corresponding DMSO control group (Figure 9C). However, the increase in proliferation observed in young fibroblasts was 1.23%, which was significantly lower than the increase of 37.78% observed in senescent fibroblasts (Figure 1B and Figure 9C). This difference may be related to the fact that the population doubling time for young fibroblasts is less than 2 days [20,21,22,23]. Since young fibroblasts already exhibit high basal proliferative capacity and double in cell number within 2 days, the range of additional proliferation increase that can be induced by KB3409 treatment may be limited. On the other hand, since senescent fibroblasts have significantly reduced basal proliferative capacity, the recovery effect following KB3409 treatment may appear relatively large.

### 3.10. VCP-Dependent and -Independent Effects of KB3409 on Cellular Rejuvenation in Senescent Fibroblasts

**Figure 10:**
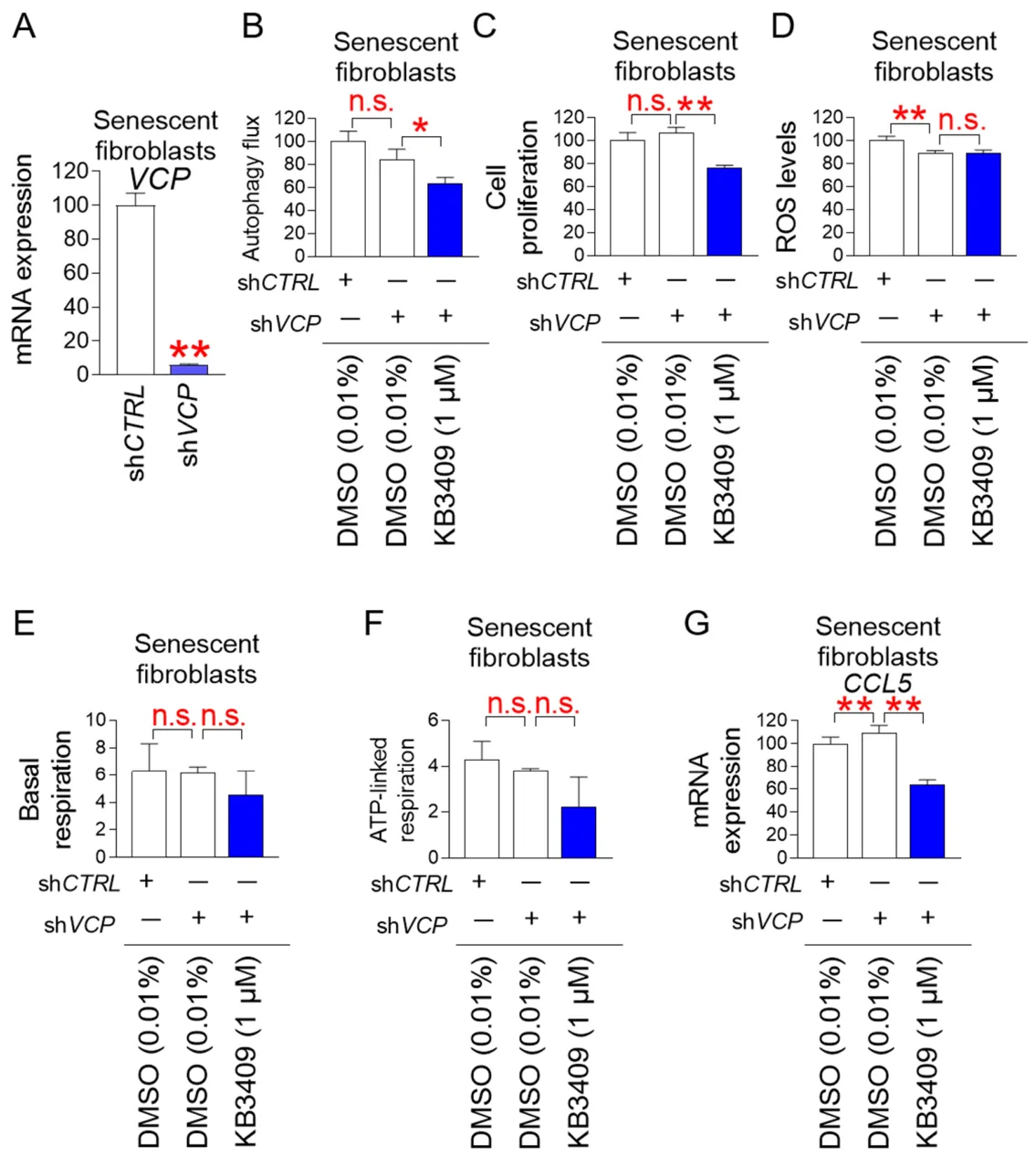
VCP-dependent and -independent effects of KB3409 on cellular rejuvenation in senescent fibroblasts. (**A**) Senescent fibroblasts were transduced with lentivirus expressing sh*CTRL* or sh*VCP*. mRNA expression levels of *VCP.* For statistical analysis, Student’s *t*-test was used. ** *p* < 0.01 was considered significant. Mean ± standard deviation, *n* = 3. (**B**–**G**) Senescent fibroblasts transduced with sh*CTRL* or sh*RCN2* were treated with DMSO (0.01%) or KB3409 (1 μM). Autophagy flux (**B**), cell proliferation (**C**), ROS levels (**D**), basal respiration (**E**), ATP-linked respiration (**F**) and *CCL5* mRNA expression (**G**) were measured. Statistical analysis was performed using Bonferroni post hoc tests following one-way ANOVA. n.s. indicates not significant. * *p* < 0.05 and ** *p* < 0.01 were considered significant. Mean ± standard deviation, *n* = 3.

Following confirmation of the interaction between KB3409 and VCP, as well as the KB3409-mediated increases in VCP expression and activity, we investigated the role of VCP in KB3409-induced cellular rejuvenation. To this end, senescent fibroblasts were transduced with lentiviruses expressing either sh*CTRL* or sh*VCP*. Endogenous *VCP* mRNA expression was efficiently silenced by shRNA targeting VCP, as confirmed by the reduced *VCP* expression in sh*VCP*-transduced senescent fibroblasts compared with the sh*CTRL* group (Figure 10A).

To determine whether the KB3409-mediated enhancement of autophagic activity depends on VCP, we first assessed autophagic flux in senescent fibroblasts transduced with sh*CTRL* or sh*VCP*. *VCP* knockdown alone did not significantly alter autophagic flux compared with sh*CTRL* transduction (Figure 10B). However, in sh*VCP*-transduced senescent fibroblasts, KB3409 treatment significantly reduced autophagic flux compared with the corresponding DMSO-treated control group (Figure 10B). These findings indicate that the effect of KB3409 on autophagic flux is altered by VCP depletion and may reflect a context-dependent response to *VCP* deficiency.

We next examined whether VCP is required for the effect of KB3409 on cell proliferation. *VCP* knockdown alone did not significantly affect cell proliferation compared with sh*CTRL* transduction (Figure 10C). However, KB3409 treatment significantly reduced cell proliferation in sh*VCP*-transduced senescent fibroblasts compared with the corresponding DMSO-treated control group (Figure 10C). These findings indicate that the proliferative response to KB3409 is altered by *VCP* knockdown and may reflect a context-dependent response to *VCP* deficiency.

We then examined whether VCP is required for the effect of KB3409 on intracellular ROS levels. ROS levels were significantly lower in sh*VCP*-transduced senescent fibroblasts than in sh*CTRL*-transduced cells. However, KB3409 treatment did not further reduce ROS levels in sh*VCP*-transduced senescent fibroblasts compared with the corresponding DMSO-treated control group (Figure 10D). This finding suggests that the effect of KB3409 on ROS levels is dependent, at least in part, on *VCP* expression.

Next, we examined whether VCP is required for the effects of KB3409 on mitochondrial respiration. *VCP* knockdown alone did not significantly alter basal respiration compared with sh*CTRL* transduction. However, in sh*VCP*-transduced senescent fibroblasts, KB3409 treatment failed to increase basal respiration compared with the corresponding DMSO-treated control group (Figure 10E). Similarly, *VCP* knockdown alone did not significantly affect ATP-linked respiration, whereas KB3409 treatment failed to increase ATP-linked respiration in sh*VCP*-transduced senescent fibroblasts compared with the corresponding DMSO-treated control group (Figure 10F). Collectively, these findings suggest that the effects of KB3409 on basal and ATP-linked respiration require VCP or are at least strongly dependent on *VCP* expression.

Finally, we examined whether VCP is required for the effect of KB3409 on *CCL5* mRNA expression, a marker associated with cellular senescence. *CCL5* mRNA expression was significantly higher in sh*VCP*-transduced senescent fibroblasts than in sh*CTRL*-transduced cells (Figure 10G). Nevertheless, KB3409 treatment significantly reduced *CCL5* mRNA expression in sh*VCP*-transduced senescent fibroblasts compared to the corresponding DMSO-treated control group (Figure 10G). These findings indicate that the suppressive effect of KB3409 on *CCL5* mRNA expression is maintained despite *VCP* knockdown and may therefore occur, at least in part, independently of VCP.

### 3.11. RCN2-Dependent and -Independent Effects of KB3409 on Cellular Rejuvenation in Senescent Fibroblasts

Following confirmation of the interaction between KB3409 and RCN2, as well as the KB3409-induced reductions in mitochondrial Ca^2+^ accumulation and mPTP opening, we investigated the role of RCN2 in KB3409-induced cellular rejuvenation.

**Figure 11:**
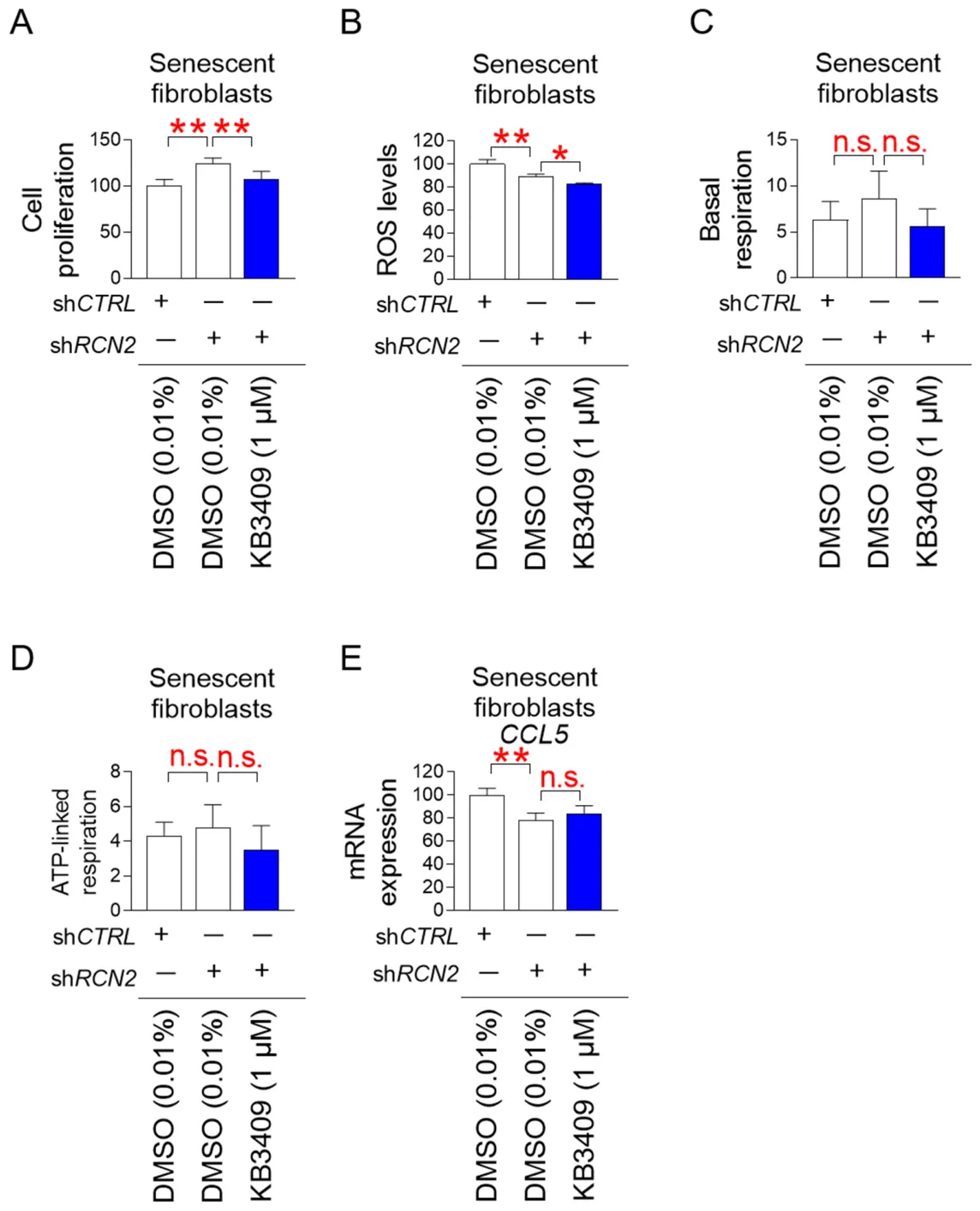
RCN2-dependent and -independent effects of KB3409 on cellular rejuvenation in senescent fibroblasts. Senescent fibroblasts transduced with sh*CTRL* or sh*RCN2* were treated with DMSO or KB3409. Cell proliferation (**A**), ROS levels (**B**), basal respiration (**C**), ATP-linked respiration (**D**) and *CCL5* mRNA expression (**E**) were measured. Statistical analysis was performed using Bonferroni post hoc tests following one-way ANOVA. n.s. indicates not significant. * *p* < 0.05 and ** *p* < 0.01 were considered significant. Mean ± standard deviation, *n* = 3.

We first examined whether RCN2 is required for the effect of KB3409 on cell proliferation. Cell proliferation was significantly higher in sh*RCN2*-transduced senescent fibroblasts than in sh*CTRL*-transduced cells (Figure 11A). However, KB3409 treatment significantly reduced cell proliferation in sh*RCN2*-transduced senescent fibroblasts compared to the corresponding DMSO-treated control group (Figure 11A). These findings indicate that the pro-proliferative effect of KB3409 was lost following *RCN2* knockdown, suggesting that RCN2 is required for the pro-proliferative effect of KB3409 under the experimental conditions tested.

We next examined whether RCN2 is required for the effect of KB3409 on intracellular ROS levels. ROS levels were significantly lower in sh*RCN2*-transduced senescent fibroblasts than in sh*CTRL*-transduced cells (Figure 11B). Moreover, KB3409 treatment further reduced ROS levels in sh*RCN2*-transduced senescent fibroblasts compared to the corresponding DMSO-treated control group (Figure 11B). These findings indicate that the ROS-lowering effect of KB3409 is maintained following *RCN2* knockdown and may therefore occur, at least in part, independently of RCN2.

We then examined whether RCN2 is required for the effects of KB3409 on mitochondrial respiration. *RCN2* knockdown alone did not significantly alter basal respiration compared with sh*CTRL* transduction (Figure 11C). However, in sh*RCN2*-transduced senescent fibroblasts, KB3409 treatment failed to increase basal respiration compared with the corresponding DMSO-treated control group, suggesting that the stimulatory effect of KB3409 on basal respiration was not maintained following *RCN2* knockdown (Figure 11C). Similarly, *RCN2* knockdown alone did not significantly affect ATP-linked respiration compared with sh*CTRL* transduction (Figure 11D). In sh*RCN2*-transduced senescent fibroblasts, KB3409 treatment also failed to increase ATP-linked respiration compared with the corresponding DMSO-treated control group (Figure 11D). Collectively, these findings suggest that the beneficial effects of KB3409 on both basal and ATP-linked respiration require RCN2 or are at least strongly dependent on RCN2 expression.

Finally, we examined whether RCN2 is required for the effect of KB3409 on *CCL5* mRNA expression, a marker associated with cellular senescence. *CCL5* mRNA expression was significantly lower in sh*RCN2*-transduced senescent fibroblasts than in sh*CTRL*-transduced cells (Figure 11E). However, KB3409 treatment did not further alter *CCL5* mRNA expression in sh*RCN2*-transduced senescent fibroblasts compared to the corresponding DMSO-treated control group (Figure 11E). These findings indicate that the suppressive effect of KB3409 on *CCL5* mRNA expression was not observed following RCN2 knockdown, suggesting that RCN2 may be required for KB3409-mediated suppression of *CCL5* mRNA expression.

## 4. Discussion

Mitochondria are the primary source of intracellular ROS, which are predominantly generated as byproducts of ETC activity [54]. Electron leakage from complexes I and III results in the partial reduction in molecular oxygen, producing superoxide anions within the mitochondrial matrix and intermembrane space. Under conditions of mitochondrial dysfunction, impaired electron flow through the ETC leads to electron accumulation and increased premature electron transfer to oxygen, thereby exacerbating superoxide generation [54]. Excess mitochondrial ROS damages mitochondrial DNA, proteins, and lipid membranes, further compromising ETC function and establishing a self-amplifying cycle of oxidative stress and bioenergetic decline that contributes to cellular senescence [40]. These observations underscore the importance of limiting mitochondrial ROS production as a strategy to mitigate senescence-associated phenotypes. In this study, we sought to identify small molecules capable of reducing ROS. Through chemical library screening of oxazole- and pyrazole-based compounds, we identified the pyrazole derivative KB3409 as a potent candidate. Biochemical analyses revealed that KB3409 binds to an allosteric site of VCP, enhancing its ATPase activity to a degree comparable to that of the known VCP activator UP163. VCP plays a central role in ubiquitin-dependent protein quality control by extracting and remodeling ubiquitinated substrates, thereby facilitating their processing through autophagic pathways [32]. Notably, activation of VCP enhances autophagic flux, including mitophagy, enabling the selective removal of damaged mitochondria. By promoting mitochondrial quality control, VCP activation reduces the burden of dysfunctional mitochondria, a major source of intracellular ROS. To our knowledge, this study is the first to demonstrate that KB3409 enhances mitochondrial quality control by activating VCP ATPase activity and promoting VCP-dependent autophagic flux. The identification of KB3409 as a functional regulator of the VCP–autophagy–mitophagy axis highlights its potential as a therapeutic strategy for mitigating oxidative stress and senescence-associated dysfunction.

Maintaining mitochondrial Ca^2+^ homeostasis is essential for proper mitochondrial function, as physiological Ca^2+^ levels act as cofactors for key metabolic enzymes and support efficient OXPHOS [55]. In contrast, excessive mitochondrial Ca^2+^ accumulation triggers opening of the mPTP, leading to increased inner membrane permeability, loss of MMP, and impaired OXPHOS [55]. RCN2, a Ca^2+^-binding protein containing six EF-hand motifs, has been implicated in regulating Ca^2+^ transfer at ER–mitochondria contact sites and may contribute to mitochondrial Ca^2+^ overload [39]. In this study, we demonstrate that KB3409 directly binds to RCN2. Functionally, KB3409 treatment significantly reduced mitochondrial Ca^2+^ levels and suppressed mPTP opening. To determine whether RCN2 is required for these effects, RCN2-silenced senescent fibroblasts were treated with DMSO or KB3409. In shCTRL-transduced cells, KB3409 significantly reduced mitochondrial Ca^2+^ levels and inhibited mPTP opening compared to DMSO-treated controls. In contrast, these effects were abolished in RCN2-depleted cells, indicating that RCN2 is required for KB3409-mediated attenuation of mitochondrial Ca^2+^ overload and mPTP opening. These findings suggest that KB3409 has therapeutic potential for maintaining mitochondrial Ca^2+^ homeostasis. However, further studies are needed to elucidate the molecular mechanism by which KB3409 regulates RCN2 activity. Collectively, our results provide a conceptual framework for the development of KB3409-based therapeutic strategies targeting aging and age-related diseases.

Mitochondria are the primary energy-producing organelles of cells and provide a stable supply of ATP [56]. However, senescence-related mitochondrial dysfunction reduces OXPHOS efficiency, thereby decreasing ATP production [53]. Consequently, senescent cells increase their reliance on glycolysis to compensate for energy deficiency. This metabolic reprogramming accelerates the decline of cellular function and leads to an imbalance in energy metabolism [57]. Furthermore, this imbalance in energy metabolism is not only a consequence of senescence but also acts as a factor that accelerates the progression of senescence [57]. Therefore, metabolic reprogramming strategies to correct metabolic abnormalities are presented as a promising approach for the treatment of aging and age-related diseases. In this study, we found that RCN2 regulation via KB3409 inhibits mitochondrial Ca^2+^ overload and restores MMP, thereby improving OXPHOS efficiency. These changes are interpreted as reprogramming the energy metabolism of senescent fibroblasts by reducing their reliance on glycolysis. In summary, KB3409 can serve as a potential strategy to mitigate the aging phenotype through metabolic reprogramming.

## 5. Conclusions

In summary, this study elucidated the mechanism by which the pyrazole analog KB3409 alleviates senescence-associated phenotypes by improving mitochondrial dysfunction. Mechanistically, KB3409 enhances mitochondrial function through two complementary pathways. First, it activates VCP-dependent autophagy flow to strengthen mitochondrial quality control and promote the selective removal of degraded mitochondria. Second, through interaction with RCN2, it reduces Ca^2+^ accumulation within mitochondria and inhibits the opening of mPTPs, thereby preserving MMP and respiratory function. These combined effects lead to a reduction in ROS levels, highlighting the central role of dysfunctional mitochondria clearance and Ca^2+^ homeostasis in regulating cellular senescence. Although further research is needed to elucidate the precise molecular interactions between KB3409, RCN2, and VCP, this study suggests that the regulation of VCP activity and RCN2-mediated Ca^2+^ dynamics could be a potential therapeutic strategy to target age-related diseases.

## Figures and Tables

**Table 1:** Culture condition for human dermal fibroblasts.

Components	Catalogue Number
Dulbecco’s Modified Eagle Medium (25 mM glucose)	MD10000A, Bandio, Seoul, Republic of Korea
10% fetal bovine serum	BAN.SR10000A, Bandio
antibiotics (penicillin 100 U/mL and streptomycin 100 µg/mL)	SV30079.01, HyClone, Waltham, MA, USA

**Table 2:** Information on the analysis of ROS, mitochondrial mass, MMP, and autophagy flux.

Analysis	Dye Name	Concentration	Catalogue Number	Company
ROS	DHR123	30 µM	10056-1	Biotium, Fremont, CA, USA
Mitochondrial mass	MitoTracker^TM^ Deep Red FM Dye	50 nM	M46753	Invitrogen, Waltham, MA, USA
MMP	JC-10	0.6 µg/mL	ENZ-52305	Enzo Life Sciences, Farmingdale, NY, USA
Autophagy flux	Cyto-ID detection reagent	Not available	ENZ-51031-0050	Enzo Life Sciences

**Table 3:** Details of primers are used to construct plasmids.

Target	Orientation	Sequence (5′–3′)
pCMV–Myc–RCN2 (Accession number: NM_002902.3)	forward	TAGTCCAGTGTGGTGGAATTATGCGGCTGGGCCC
	reverse	CGGCCGCCACTGTGCTGGATGTGATGGTGATGGTGATGTTAAAGCTCATCATGATAGAAATAGTCATCATGGAG
pCMV–Myc–VCP (Accession number: NM_007126.5)	forward	TAGTCCAGTGTGGTGGAATTATGGCTTCTGGAGCCGATTC
	reverse	CGGCCGCCACTGTGCTGGATGTGATGGTGATGGTGATGTTAGCCATACAGGTCATCATCATTGTCT
pLKO.1–sh*RCN2*(Targeting sequence: CCTTGGGTAGTACCTAATAAT)	forward	CCGGCCTTGGGTAGTACCTAATAATTCAAGAGATTATTAGGTACTACCCAAGGTTTTTG
	reverse	AATTCAAAAACCTTGGGTAGTACCTAATAATCTCTTGAATTATTAGGTACTACCCAAGG
pLKO.1–sh*RCN2*(Targeting sequence: CCGGTTTGAGTCTTGAAGAAT)	forward	TGGAAAGGACGAAACACCGGCCGGTTTGAGTCTTGAAGAATTCAAGAGATTCTTCAAGACTCAAACCGG
	reverse	TTGTCTCGAGGTCGAGAATTCCGGTTTGAGTCTTGAAGAATCTCTTGAATTCTTCAAGACTCAAACCGG
pLKO.1–sh*VCP *(Targeting sequence: GAATAGAGTTGTTCGGAATAA)	forward	TGGAAAGGACGAAACACCGGGAATAGAGTTGTTCGGAATAAtcaagagTTATTCCGAACAACTCTATTC
	reverse	TGTCTCGAGGTCGAGAATTGAATAGAGTTGTTCGGAATAActcttgaTTATTCCGAACAACTCTATTC
pLKO.1–sh*VCP* Targeting sequence: ACCGTCCCAATCGGTTAATTG)	forward	TGGAAAGGACGAAACACCGGACCGTCCCAATCGGTTAATTGTCAAGAGCAATTAACCGATTGGGACGGT
	reverse	TTGTCTCGAGGTCGAGAATTACCGTCCCAATCGGTTAATTGCTCTTGACAATTAACCGATTGGGACGGT

**Table 4:** Details regarding Western blot analysis.

Target	Antibody	Dilution	Catalogue Number	Company
β-actin	HRP-conjugated β-actin antibody	1:1000	sc-47778	Santa Cruz Biotechnology, Dallas, TX, USA
Myc tag	anti-Myc tag	1:1000	2276S	Cell Signaling Technology, Danvers, MA, USA
	HRP-conjugated anti-mouse IgG antibody	1:2000	sc-358914	Santa Cruz Biotechnology
RCN2	anti-RCN2	1:1000	A19943	Abclonal, Boston, MA, USA
	HRP-conjugated anti-rabbit IgG antibody	1:2000	sc-2357	Santa Cruz Biotechnology
VCP	anti-VCP	1:1000	A2795	Abclonal
	HRP-conjugated anti-rabbit IgG antibody	1:2000	sc-2357	Santa Cruz Biotechnology
LC3B	anti-LC3B rabbit antibody	1:1000	A19665	Abclonal
	HRP-conjugated anti-rabbit IgG antibody	1:2000	sc-2357	Santa Cruz Biotechnology

**Table 5:** Details regarding immunofluorescence.

Target	Reagent	Dilution	Catalogue Number	Company
Calcium ions	Rhod-2 AM	1:1000	M34153	Thermo Fisher Scientific
Mitochondria	MitoTracker™ Green FM	1:10,000	M7514	Thermo Fisher Scientific
OXPHOS	anti-OXPHOS cocktail mouse antibody	1:1000	ab110411	Abcam, Cambridge, UK
	Alexa Fluor^®^ 647-conjugated anti-mouse IgG antibody	1:2500	A–28181	Invitrogen
LC3B	anti-LC3B rabbit antibody	1:1000	A19665	Abclonal
	Alexa Fluor^®^ 488-conjugated anti-rabbit IgG antibody	1:2500	A–11008	Invitrogen
PINK1	anti-PINK1 rabbit antibody	1:1000	ab216144	Abclonal
	Alexa Fluor^®^ 488-conjugated anti-rabbit IgG antibody	1:2500	A–11008	Invitrogen

**Table 6:** Lists of the specific primers used in qPCR.

Target	Orientation	Sequence (5′–3′)	Size (bp)
*36B4* (Accession number: NM_053275)	forward	CAGCAAGTGGGAAGGTGTAATCC	23
	reverse	CCCATTCTATCATCAACGGGTACAA	25
*p21* (Accession number: NM_000389.5)	forward	AGGTGGACCTGGAGACTCTCAG	22
	reverse	TCCTCTTGGAGAAGATCAGCCG	22
*TGF-β1* (Accession number: NM_000660.7)	forward	AGCTGTACCAGAAATACAGCAACAAT	26
	reverse	CCGGTGACATCAAAAGATAACCAC	24
*MMP1* (Accession number: NM_002421.4)	forward	ATGAAGCAGCCCAGATGTGGAG	22
	reverse	TGGTCCACATCTGCTCTTGGCA	22
*MMP3* (Accession number: NM_002422.5)	forward	GATTAATGGAGATGCCCACTTTGATGA	27
	reverse	GGAGTGGCCAATTTCATGAGCA	22
*CXCL1* (Accession number: NM_001511.4)	forward	AAACCGAAGTCATAGCCACACTC	23
	reverse	TCACTGTTCAGCATCTTTTCGATGATTT	28
*CCL5* (Accession number: NM_002985.3)	forward	CCTGCTGCTTTGCCTACATTGC	22
	reverse	ACACACTTGGCGGTTCTTTCGG	22
*RCN2* (Accession number: NM_002902)	forward	AGCTAACCAGGATTCAGGTCCC	22
	reverse	TTCTTCCAAACTAACAAATCCATCACCATT	30
*VCP* (Accession number: NM_007126)	forward	AACTGCCCCTGAGACATCC	19
	reverse	GGACCATTGATCAAGAAGAAGAAGGC	26
*CypD* (Accession number: NM_005038)	forward	CAGAATGGGACAGGTGGAGAAAG	23
	reverse	CTGAGAACCGTTTGTGTTGCGG	22

## Data Availability

The original contributions presented in the study are included in the article, further inquiries can be directed to the corresponding authors.

## Supplementary Materials

The following supporting information can be downloaded at https://www.mdpi.com/article/10.3390/antiox15081006/s1: Figure S1: Synthesis of KB3409 and biotinylated KB3409; Figure S2: ^1^H NMR spectra of compound 1; Figure S3: ^1^H NMR spectra of compound 2(KB3409); Figure S4: HPLC data of compound 2(KB3409); Figure S5: HRMS data of compound 2 (KB3409); Figure S6: ^1^H NMR spectra of compound 3; Figure S7: ^1^H NMR spectra of compound 4(biotinylated KB3409); Figure S8: HPLC spectra of compound 4 (biotinylated KB3409); Figure S9: HRMS spectra of compound 4 (biotinylated KB3409); Figure S10: Characteristics of senescent and young fibroblasts; Figure S11: Measurement of the solubility of KB3409; Figure S12: Measurement of the excitation and emission wavelengths of KB3409; Figure S13: mRNA and protein expression levels of RCN2 and VCP were measured in senescent fibroblasts treated with DMSO (0.01%) or KB3409 (1 μM) for 12 days at 4-day intervals; Figure S14: Full-size image of Western blot. (A) Figure S13B, (B) Figure S13D; Figure S15: Full-size image of western blot. (A) Figure 2E, (B) Figure 2F; Figure S16: Immunofluorescence analysis of LC3B (green fluorescence) and mitochondria (red fluorescence) in senescent fibroblasts treated with DMSO (0.01%) or KB3409 (1 μM) for 12 days at 4-day intervals; Figure S17: Protein expression levels of LC3B-I and II; Figure S18: Immunofluorescence analysis of PINK1 (green fluorescence) and mitochondria (red fluorescence) in senescent fibroblasts treated with DMSO (0.01%) or KB3409 (1 μM) for 12 days at 4-day intervals; Figure S19: Full-size image of immunofluorescence in Figure S18; Figure S20: Full-size image of immunofluorescence in Figure 4C; Figure S21: Full-size image of immunofluorescence in Figure 5A; Figure S22: Full-size image of immunofluorescence in Figure 6B; Table S1: Estimated Solubility of the Compounds Predicted Using ChemDraw; Table S2: The detailed list of ROS level in high throughput screening; Table S3: List of 43 proteins that bind only to biotinylated KB3409 and not to biotin; Table S4: List of 95 proteins that bind only to biotin and not to biotinylated KB3409; Table S5: List of 82 proteins that bind to both biotinylated KB3409 and biotin.
