# Supplementary material for: A Pyrazole-Based Small Molecule, KB3409, Restores Mitochondrial Function via VCP Activation and RCN2-Dependent Ca2+ Regulation
Source: Antioxidants (Basel). 2026 Aug 13;15(8):1006. doi: 10.3390/antiox15081006 (PMC13509518; doi:10.3390/antiox15081006)
Supplement: Supplementary file 1 [file antioxidants-15-01006-s001.zip › antioxidants-4421021-supplementary.pdf]

Supplementary experimental procedures

Procedure for the synthesis of KB3409 and biotinylated KB3409

All reagents and analytical-grade solvents were purchased from commercial suppliers and used without further purification unless otherwise specified. Reaction progress was monitored by thin-layer chromatography (TLC) on pre-coated silica gel 60F254 plates (Merck, Darmstadt, Germany), with visualization under UV light at 254 or 365 nm. Column chromatography was performed on silica gel (Silica gel 60:230-400 mesh ASTM, Merck, Darmstadt, Germany). ^{1}H and ^{13}C nuclear magnetic resonance (NMR) spectra were recorded at room temperature using a Bruker Ultrashield 600 MHz Plus spectrometer. Chemical shifts (δ) are reported in parts per million (ppm) relative to tetramethylsilane (TMS) or residual solvent peaks ($CDCl_3$: δ = 7.26 for ^{1}H NMR, δ = 77.16 for ^{13}C NMR; DMSO-d_6: δ = 2.50 for ^{1}H NMR, δ = 39.52 for ^{13}C NMR). Signal multiplicities are indicated as follows: s (singlet), brs (broad singlet), d (doublet), t (triplet), q (quartet), m (multiplet), dd (doublet of doublets), etc. High-resolution mass spectra (HRMS) were obtained using an Agilent 6530 Accurate-Mass Q-TOF LC/MS system. Compound purity was assessed by analytical reverse-phase HPLC (RP-HPLC) on an Agilent 1260 Infinity system equipped with a C18 column (Phenomenex, 150 mm × 4.6 mm, 3 μm, 110 Å), using water (0.1% TFA) and acetonitrile (0.1% TFA) as mobile phases. UV detection was performed at 220 or 245 nm.

Ethyl 2-hydroxy-4-(2-methoxyphenyl)-4-oxobut-2-enoate (*1*)

A solution of 2-methoxyacetophenone (600 μL, 4.36 mmol) in THF (10 mL) was cooled to -78 °C under argon. 1 M lithium diisopropylamide in THF/hexanes (5.22 mL, 5.22 mmol, 1.2 eq) was added dropwise while maintaining the reaction temperature. The reaction was stirred for 1 h at -78°C and then diethyl oxalate (708 μL, 5.22 mmol, 1.2 eq) was added slowly. The reaction was allowed to warm to room temperature and stirred for 12 h. The solution was partitioned between ethyl acetate and water. The organic layer washed with brine, dried over MgSO4, and evaporated. The residue was purified by column chromatography on a silica gel (Hexane/Ethyl acetate = 9:1 to 6:1, v/v) to afford compound 1 (601 mg, 55%) as yellow solid. R_f = 0.27 (Hexane/ethyl acetate = 3:1, v/v). ^{1}H NMR (600 MHz, $CDCl_3$) δ 7.89 (dd, J = 7.8, 1.7 Hz, 1H), 7.54 – 7.50 (m, 1H), 7.30 (s, 1H), 7.08 – 7.03 (m, 1H), 7.00 (d, J = 8.4 Hz, 1H), 4.38 (q, J = 7.1 Hz, 2H), 3.95 (s, 3H), 1.40 (t, J = 7.1 Hz, 3H).

Ethyl 5-(2-methoxyphenyl)-1-(4-(trifluoromethyl)phenyl)-1H-pyrazole-3-carboxylate (*2*)

A mixture of compound 1 (300 mg, 1.20 mmol), 4-(trifluoromethyl)phenyl hydrazine (902mg, 5.12 mmol, 4.3 eq), and acetic acid (293 μL, 5.12 mmol, 4.3 eq) in ethanol (10 mL) was purged with argon and stirred at 80°C for 12 h. The solution was partitioned between dichloromethane and water. The aqueous layer was further extracted with dichloromethane and the combined organics washed

with brine, dried over MgSO4, and evaporated. The residue was purified by column chromatography on a silica gel (Hexane/ethyl acetate = 8:1 to 4:1, v/v) to afford compound 2 (171 mg, 37%) as yellow solid. $R_f = 0.63$ (Hexane/Ethyl acetate = 3:1, v/v). ^{1}H NMR (600 MHz, $CDCl_3$) δ 7.55 (d, J = 8.5 Hz, 2H), 7.45 (d, J = 8.5 Hz, 2H), 7.42 – 7.38 (m, 1H), 7.32 (dd, J = 7.5, 1.7 Hz, 1H), 7.03 (td, J = 7.5, 0.9 Hz, 1H), 7.00 (s, 1H), 6.81 (d, J = 8.2 Hz, 1H), 4.46 (q, J = 7.1 Hz, 2H), 3.36 (s, 3H), 1.43 (t, J = 7.1 Hz, 3H). HRMS m/z calculated for $C_{20}H_{17}F_3N_2O_3$ $[M+H]^+$: 391.1273; found: 391.1234, $[M+Na]^+$: 413.1092; found: 413.1046.

5-(2-Methoxyphenyl)-1-(4-(trifluoromethyl) phenyl)-1H-pyrazole-3-carboxylic acid (3)

To a solution of compound 2 (171mg, 0.44mmol) in EtOH (10mL) and 1N NaOH was added 2 to 3 dropwise and stirred at room temperature for 1.5 h. The solution was partitioned between ethyl acetate and water. The aqueous layer was acidified and extracted with ethyl acetate. The combined organic layer washed with brine, dried over MgSO4, and evaporated. The residue was triturated with hexane and diethyl ether to afford compound 3 (107mg, 68 %) as pale orange solid. $R_f = 0.02$ (Hexane/Ethyl acetate = 3:1, v/v). ^{1}H NMR (600 MHz, DMSO) δ 7.77 (d, J = 8.5 Hz, 2H), 7.45 (d, J = 8.5 Hz, 2H), 7.43 (m, J = 1.5 Hz, 1H), 7.40 (dd, J = 7.5, 1.4 Hz, 1H), 7.06 (t, J = 7.4 Hz, 1H), 6.99 (d, J = 8.3 Hz, 1H), 6.96 (s, 1H), 3.30 (s, 3H). HRMS m/z calculated for $C_{18}H_{13}F_3N_2O_3$ [M+H]+: 363.0973; found: 363.0890.

5-(2-Methoxyphenyl)-N-(2-(5-((3aS,4S,6aR)-2-oxohexahydro-1H-thieno[3,4-d]imidazol-4-yl)pentanamido)ethyl)-1-(4-(trifluoromethyl)phenyl)-1H-pyrazole-3-carboxamide (4)

A mixture of compound 3 (26 mg, 0.071 mmol), HOBt (1.6 eq, 16 mg), EDC-HCl (1.6 eq, 22 mg) in DMF (5 mL) was stirred at room temperature for 10 min. To a solution was *N*-(2-aminoethyl)biotinamide (1.2 eq, 25 mg, 0.087 mmol) and the reaction mixture was stirred at room temperature for 24 h. Excess DMF was removed at reduced pressure. Column chromatography on a silica gel (DCM/MeOH=12:1 to 8:1, v/v) to afford compound 4 (21mg, 47%) as white solid. $R_f = 0.24$ (DCM/MeOH = 8:1, v/v). ^{1}H NMR (600 MHz, MeOD) δ 7.61 (d, J = 8.5 Hz, 4H), 7.45 (d, J = 8.4 Hz, 4H), 7.40 – 7.37 (m, 2H), 7.33 (dd, J = 7.5, 1.6 Hz, 2H), 7.01 (t, J = 7.3 Hz, 2H), 6.89 (d, J = 8.3 Hz, 2H), 6.85 (s, 2H), 4.35 (dd, J = 7.8, 4.8 Hz, 2H), 4.17 (dd, J = 7.9, 4.5 Hz, 2H), 3.51 – 3.45 (m, 4H), 3.38 (t, J = 5.7 Hz, 4H), 3.31 (s, 6H), 3.29 (s, 1H), 3.10 – 3.05 (m, 2H), 2.80 (dd, J = 12.7, 5.0 Hz, 2H), 2.60 (d, J = 12.7 Hz, 2H), 2.16 (tt, J = 9.7, 5.0 Hz, 4H), 1.68 – 1.45 (m, 9H), 1.40 – 1.31 (m, 4H). HRMS m/z calculated for $C_{30}H_{33}F_3N_6O_4S$ $[M+H]^+$: 631.2309; found: 631.2327, $[M+Na]^+$: 653.2128; found: 653.2145.

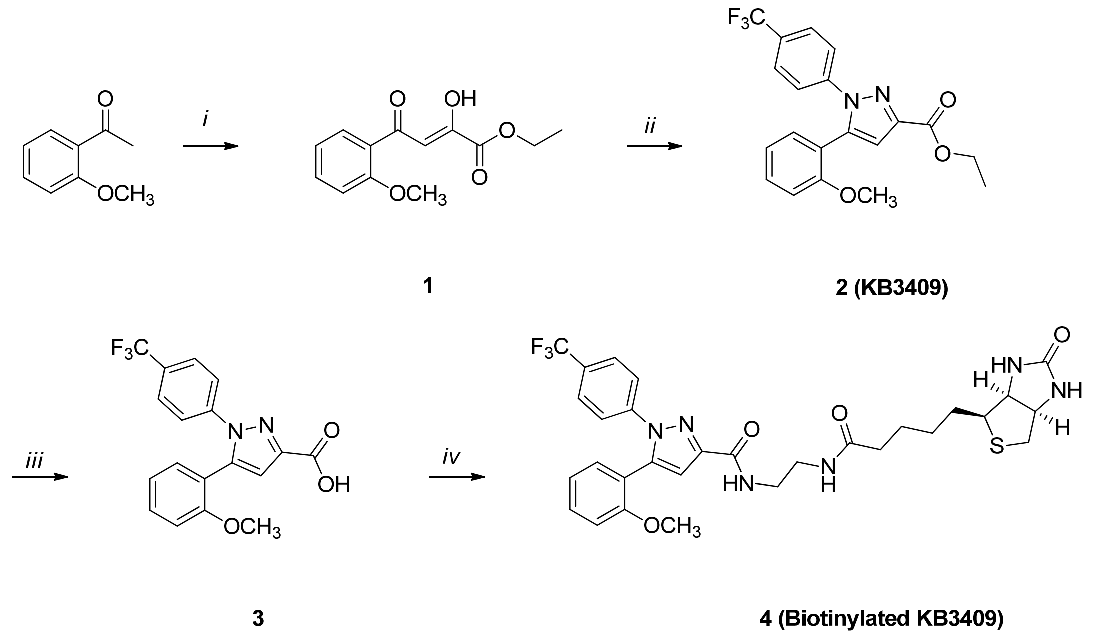

Reagents and conditions: (*i*) Lithium diisopropylamide, diethyl oxalate , THF, -78°C to r.t. Ar, 12 h; (*ii*) 4-(Trifluoromethyl)phenyl hydrazine, acetic acid, EtOH, 80°C, 12 h ; (*iii*) NaOH, EtOH, r.t., 1.5 h; (*iv*) *N*-(2-Aminoethyl)biotinamide, HOBt, EDC-HCl, DMF, r.t., 24 h

Figure S1. Synthesis of KB3409 and biotinylated KB3409.

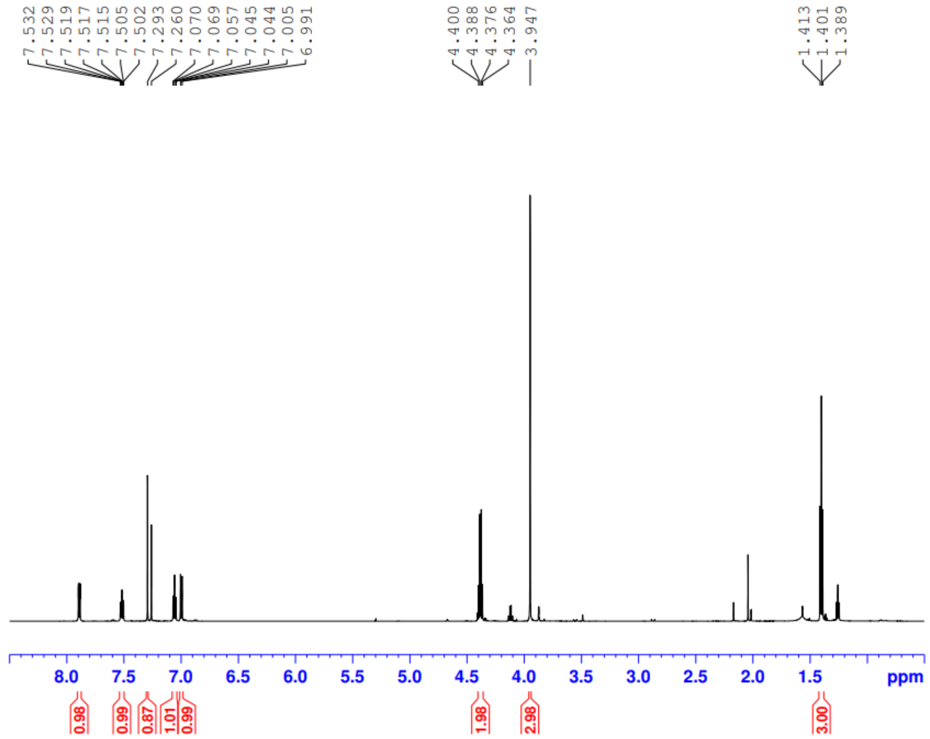

Figure S2. ^{1}H NMR spectra of compound 1.

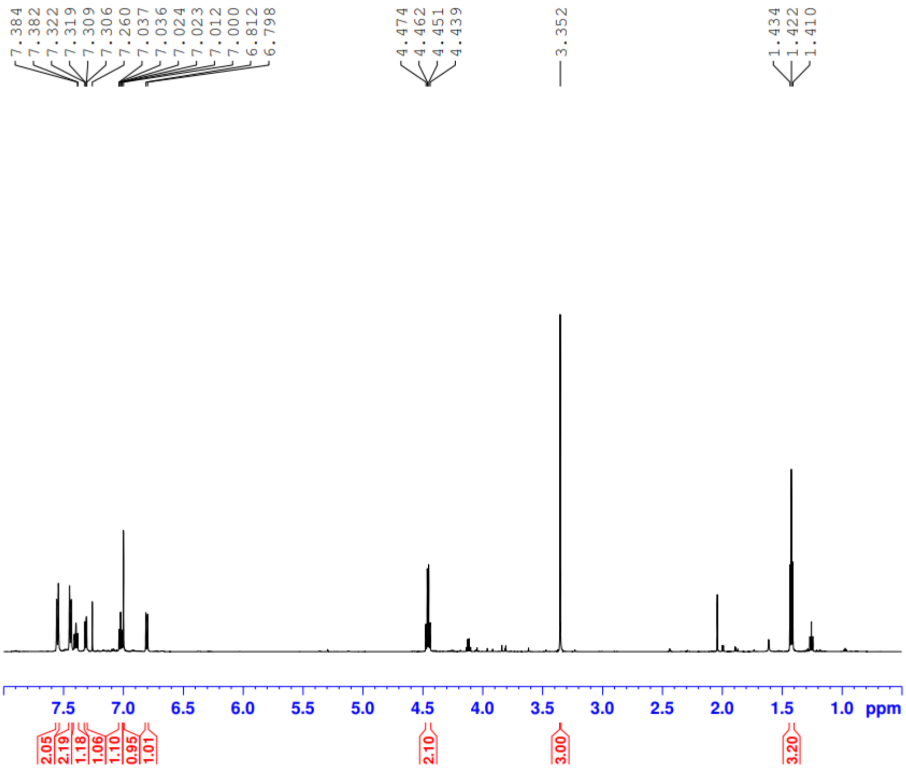

Figure S3. ^{1}H NMR spectra of compound 2(KB3409).

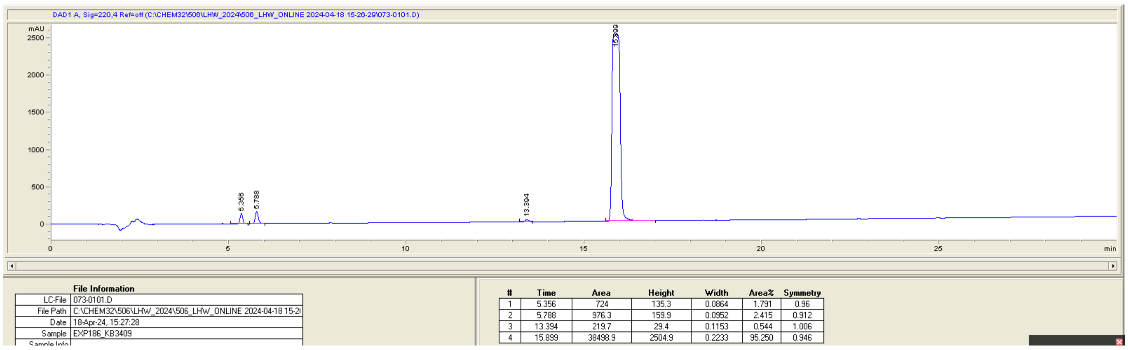

Time	Water (0.1 % TFA)	ACN (0.1 % TFA)
0	50	50
30	10	90
Flow rate : 1.0 mL/min		

Figure S4. HPLC data of compound 2(KB3409).

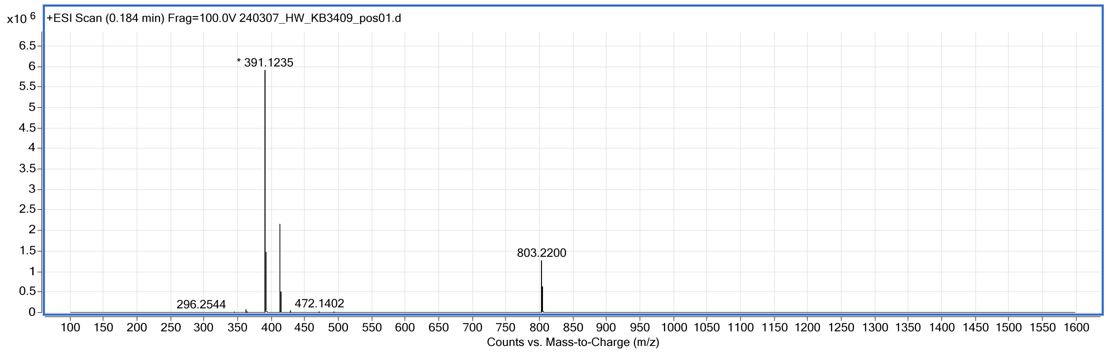

Figure S5. HRMS data of compound 2 (KB3409).

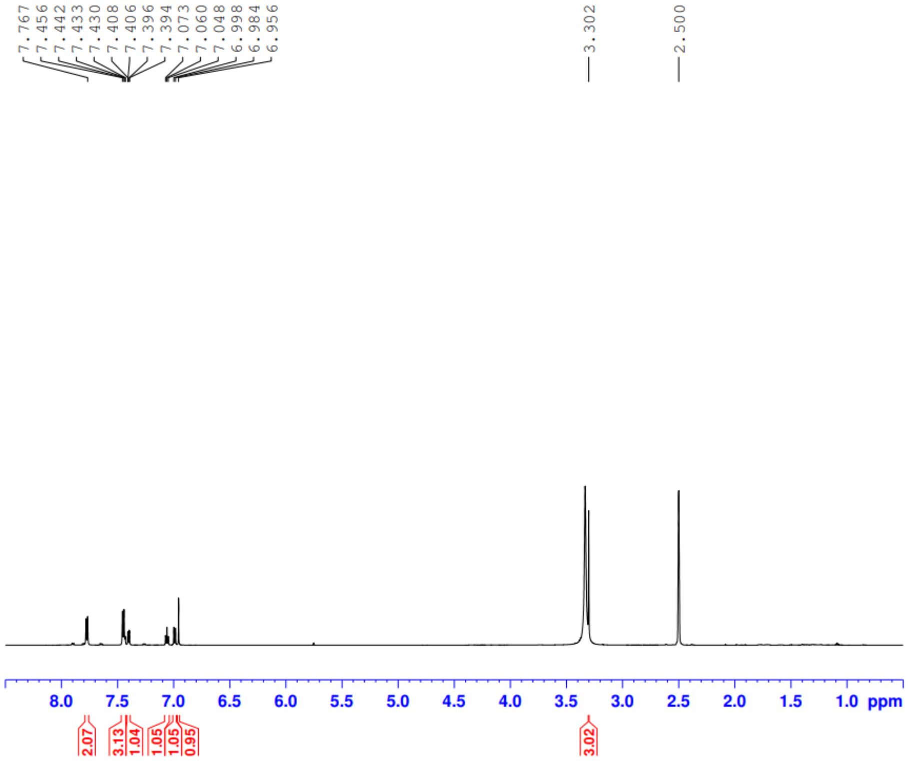

Figure S6. ^{1}H NMR spectra of compound 3.

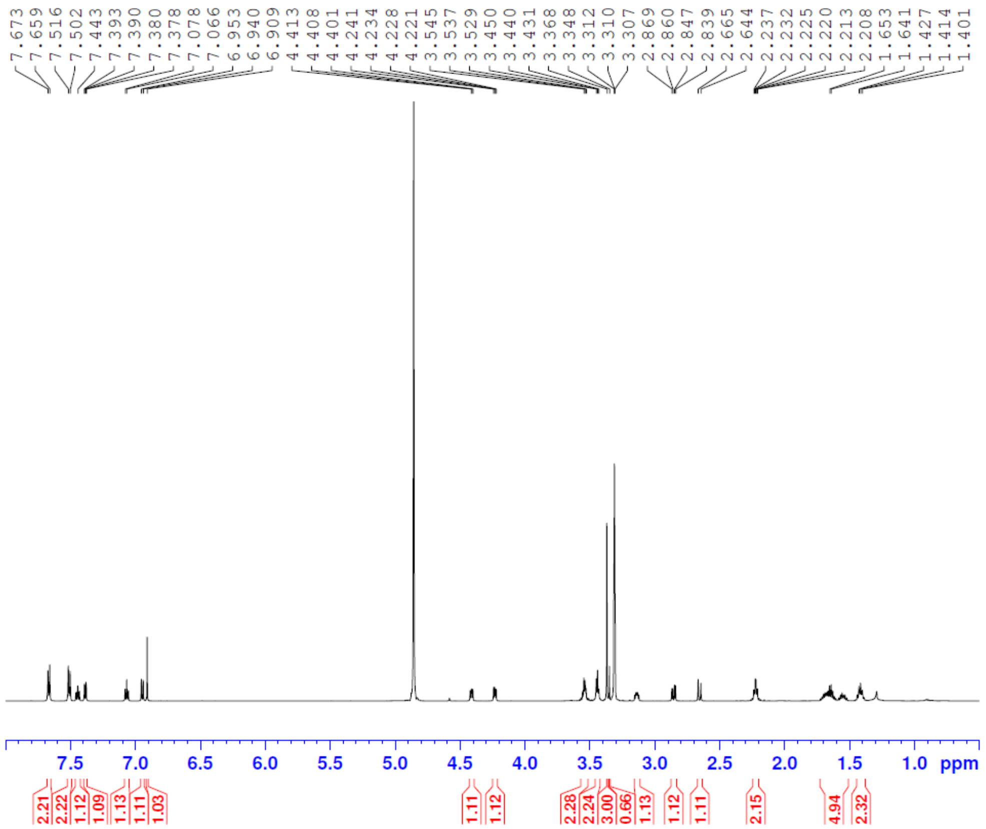

Figure S7. ^{1}H NMR spectra of compound 4(biotinylated KB3409).

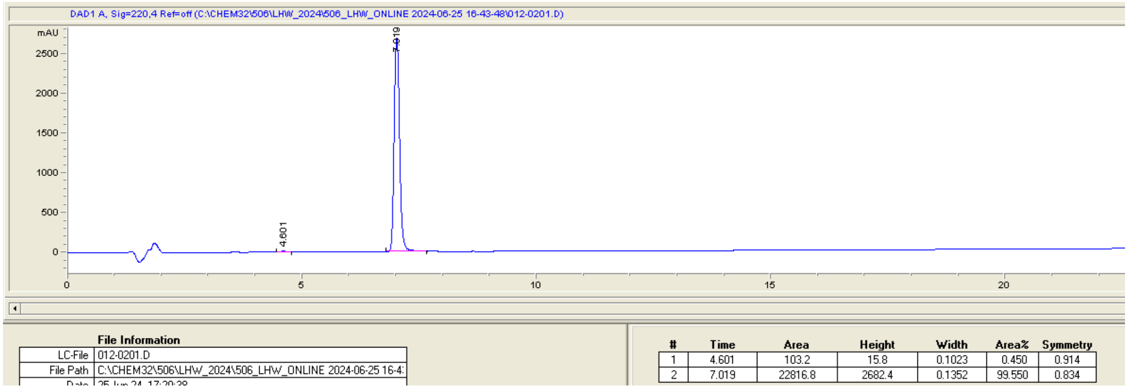

Time	Water (0.1 % TFA)	ACN (0.1 % TFA)
0	60	40
30	20	80
Flow rate : 1.0 mL/min		

Figure S8. HPLC spectra of compound 4 (biotinylated KB3409).

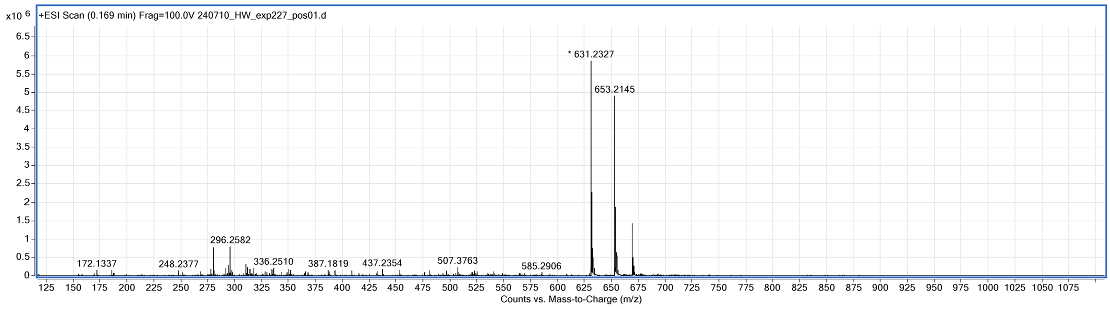

Figure S9. HRMS spectra of compound 4 (biotinylated KB3409).

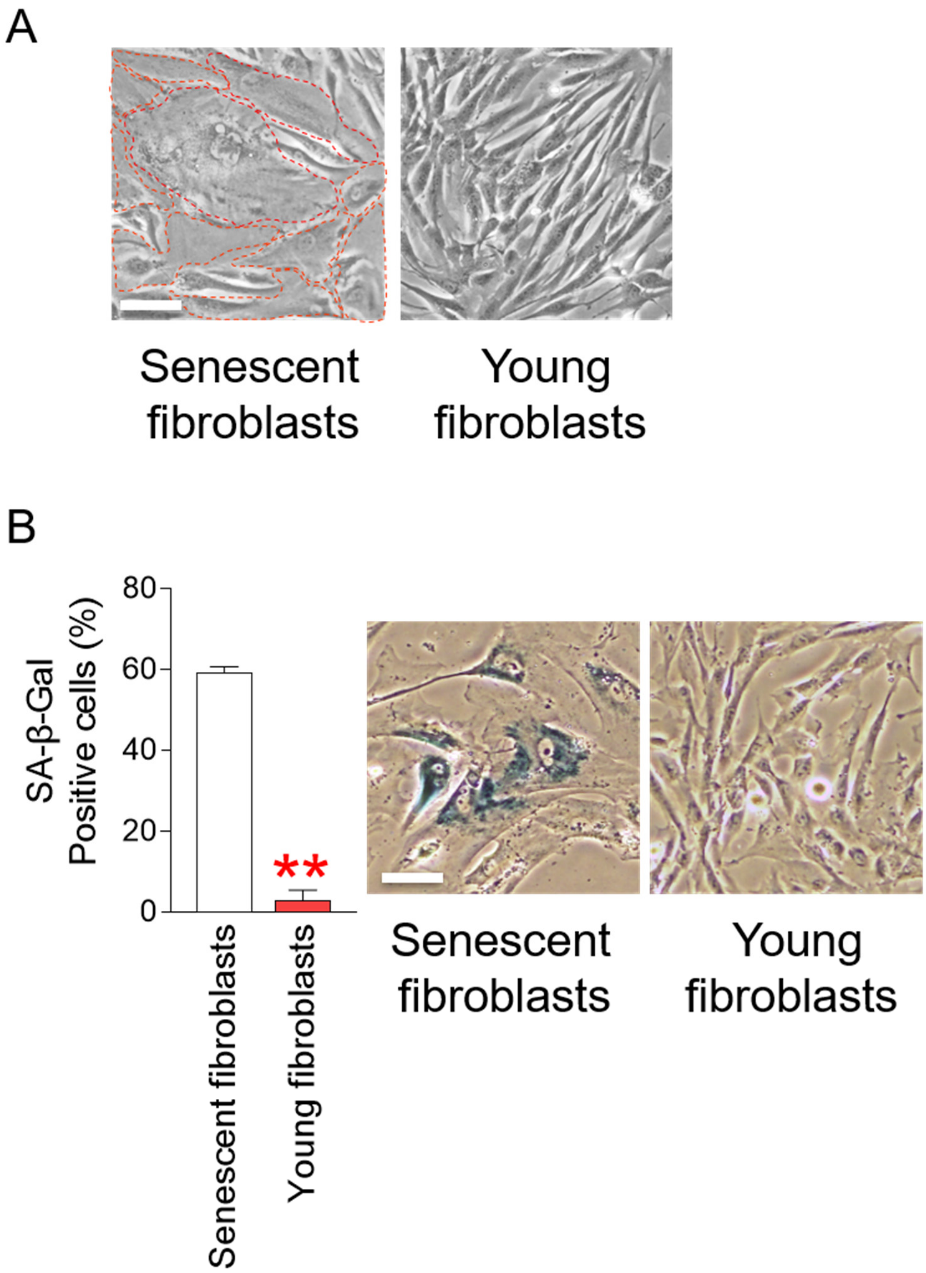

Figure S10. Characteristics of senescent and young fibroblasts. (A) Representative morphologies of senescent and young fibroblasts. Senescent fibroblasts exhibited a broad, flattened morphology (dotted lines), whereas young fibroblasts displayed a thin, spindle-shaped morphology. Scale bar, 20 μm. (B) Quantification of senescence-associated β-galactosidase (SA-β-gal)-positive cells in senescent and young fibroblasts. ** $P < 0.01$, Student's t-test. Data are presented as means ± S.D. ($n = 3$). Scale bar, 20 μm.

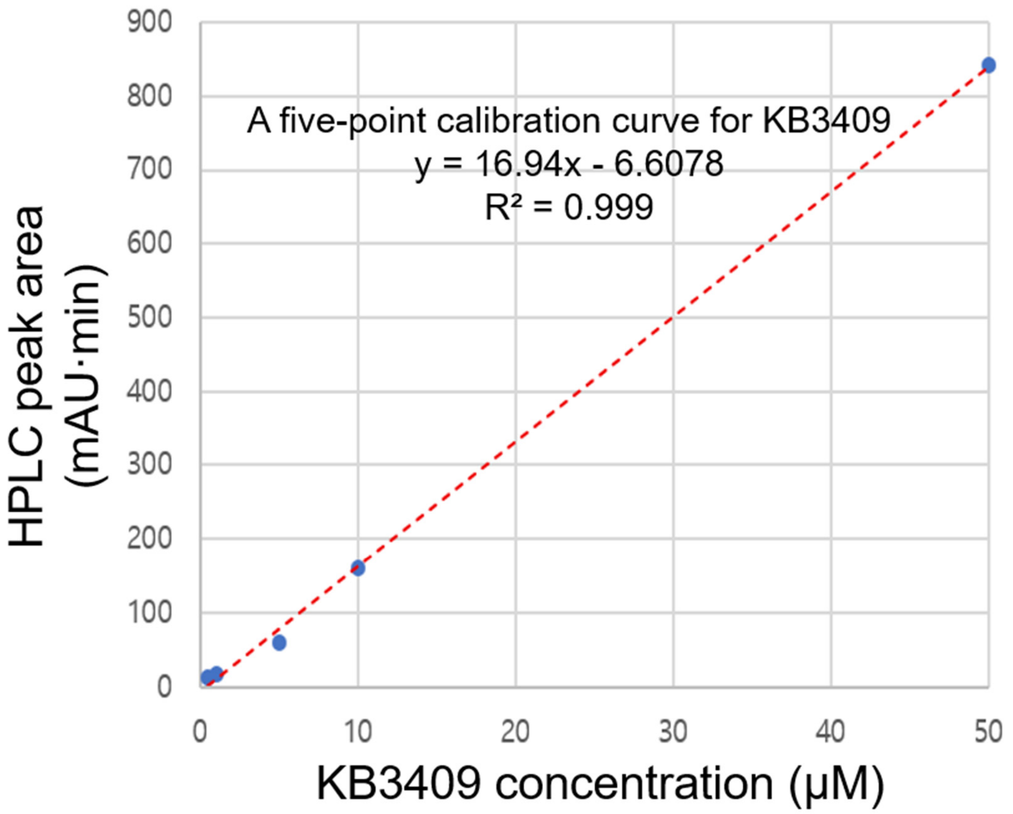

Figure S11. Measurement of the solubility of KB3409. An excess amount of KB3409 was added to 1 mL of PBS, sonicated for 2 h, and equilibrated to room temperature. The resulting suspension was filtered, and the concentration of dissolved KB3409 was quantified by high-performance liquid chromatography (HPLC). For quantification, a five-point calibration curve was prepared using KB3409 concentrations of 0.5, 1, 5, 10, and 50 µM. These standards were prepared by diluting a 10 mM KB3409 stock solution in DMSO with a 1:1 (v/v) mixture of acetonitrile and distilled water. The calibration curve was generated by plotting the HPLC detector response against the KB3409 concentration ($y = 16.94x - 6.6078$, $R^2 = 0.999$). The measured HPLC peak area was 15.60 mAU·min, corresponding to an estimated solubility of 1.311 µM.

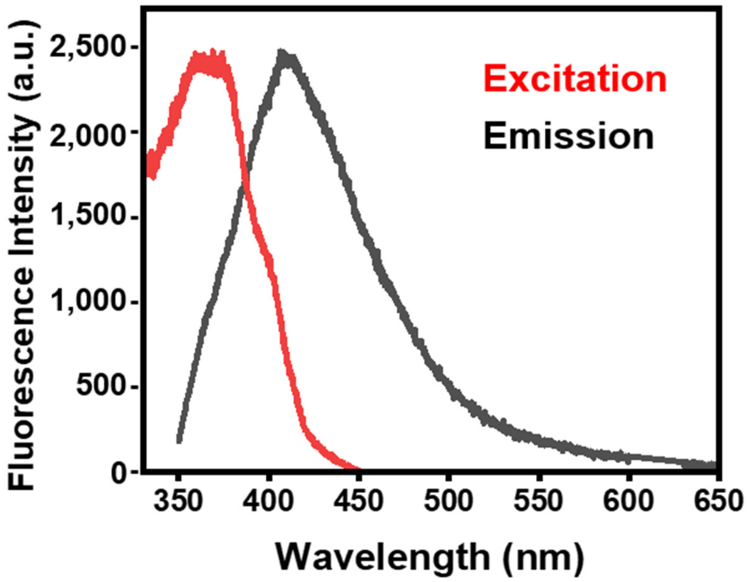

Figure S12. Measurement of the excitation and emission wavelengths of KB3409. The excitation and emission spectra of KB3409 were measured using a fluorescence spectrophotometer (F-7000; Hitachi, Tokyo, Japan). The wavelengths corresponding to the maximum fluorescence intensities were identified as the maximum excitation and emission wavelengths of KB3409.

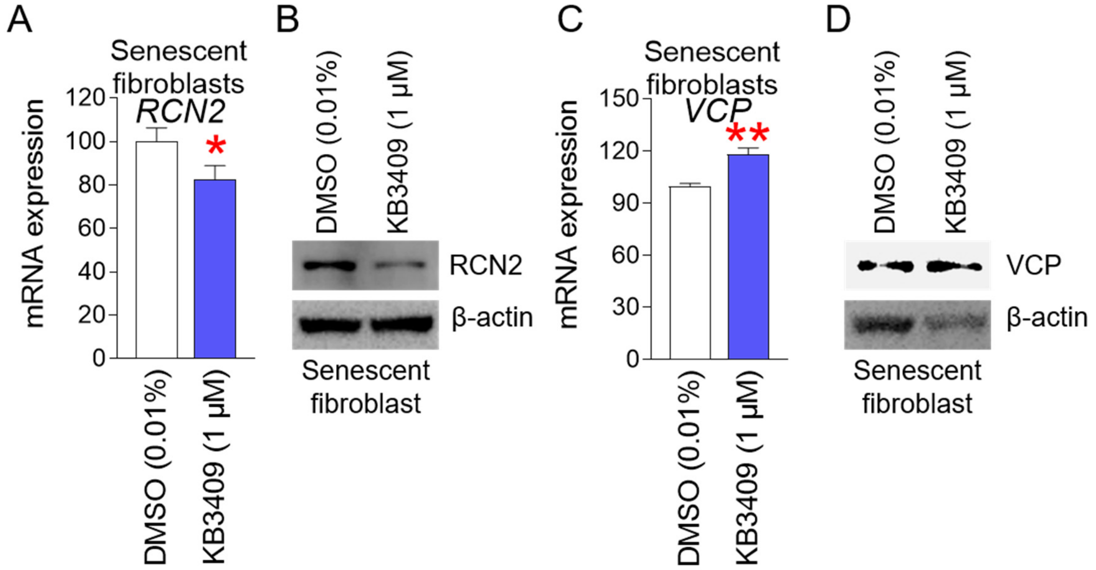

Figure S13. mRNA and protein expression levels of *RCN2* and *VCP* were measured in senescent fibroblasts treated with DMSO (0.01%) or KB3409 (1 μM) for 12 days at 4-day intervals. Statistical analysis was performed using Student's t–test, with results considered significant at * $p < 0.05$, ** $p < 0.01$. Mean ± S.D., $n = 3$. Full-size images of western blot are shown in Figure S14**.**

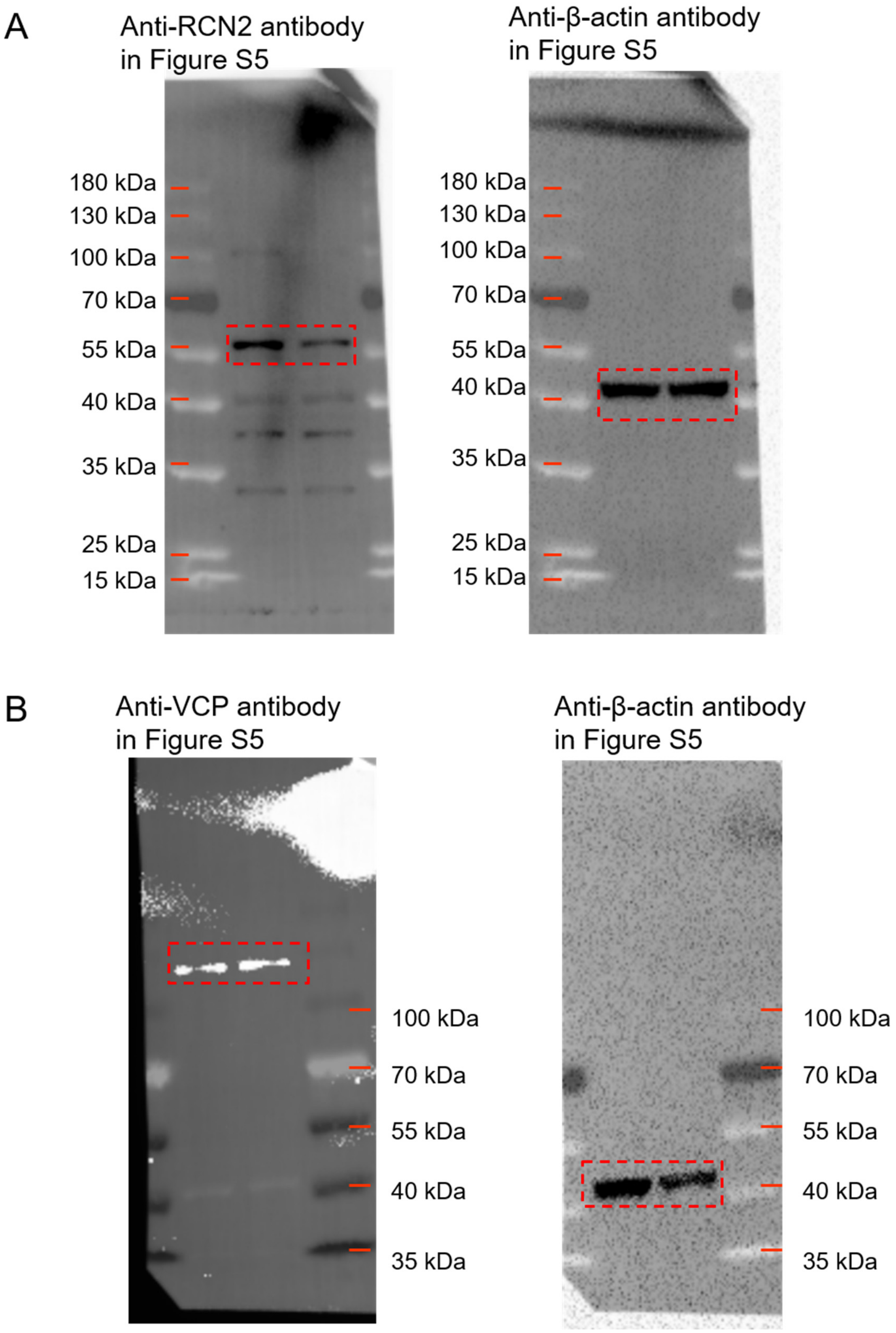

Figure S14. Full-size image of western blot. (A) Figure S13B, (B) Figure S13D.

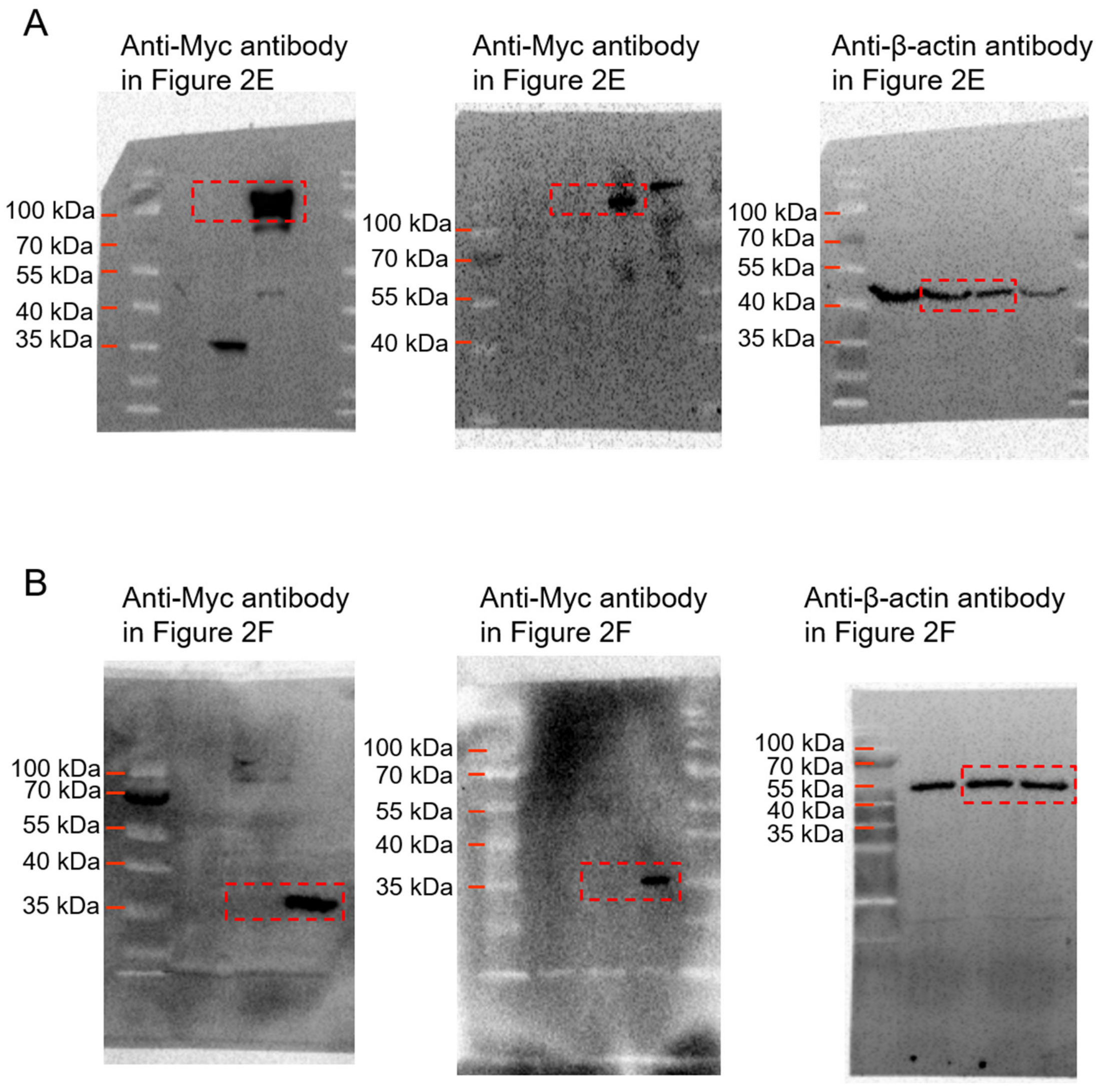

Figure S15. Full-size image of western blot. (A) Figure 2E, (B) Figure 2F.

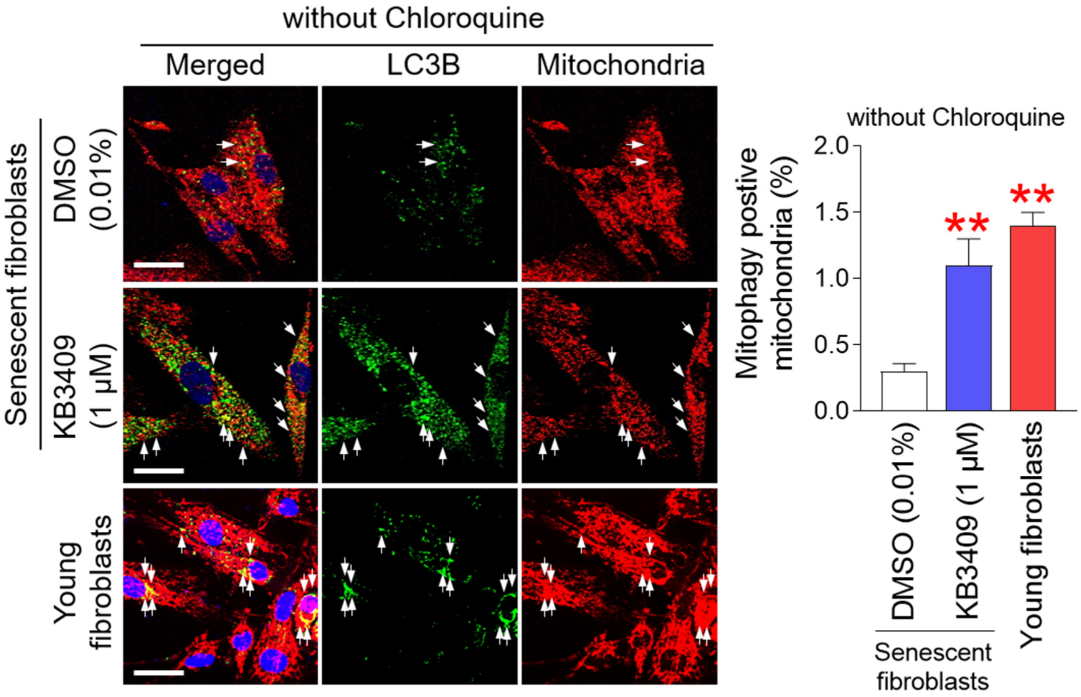

Figure S16. Immunofluorescence analysis of LC3B (green fluorescence) and mitochondria (red fluorescence) in senescent fibroblasts treated with DMSO (0.01%) or KB3409 (1 μM) for 12 days at 4-day intervals. Senescent fibroblasts were not co-treated with 20 μM chloroquine 24 h before Immunostaining. Young fibroblasts were used as a positive control. Scale bar 10 μm. Mitophagy is indicated by a white arrow. ***P* < 0.01, Student's t–test. Mean ± S.D., *n* = 3.

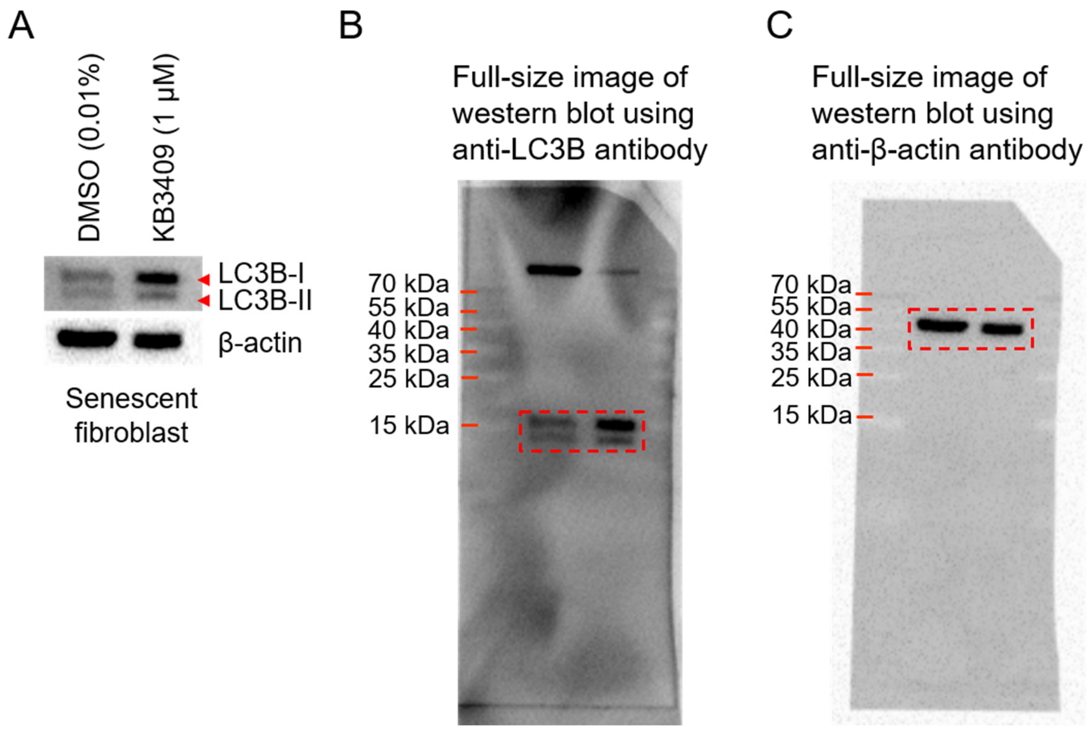

Figure S17. Protein expression levels of LC3B-I and II. (A) Protein expression levels of LC3B-I and II were measured in senescent fibroblasts treated with DMSO (0.01%) or KB3409 (1 μM) for 12 days at 4-day intervals. (B and C) Full-size images of western blot are shown in Figure S17A.

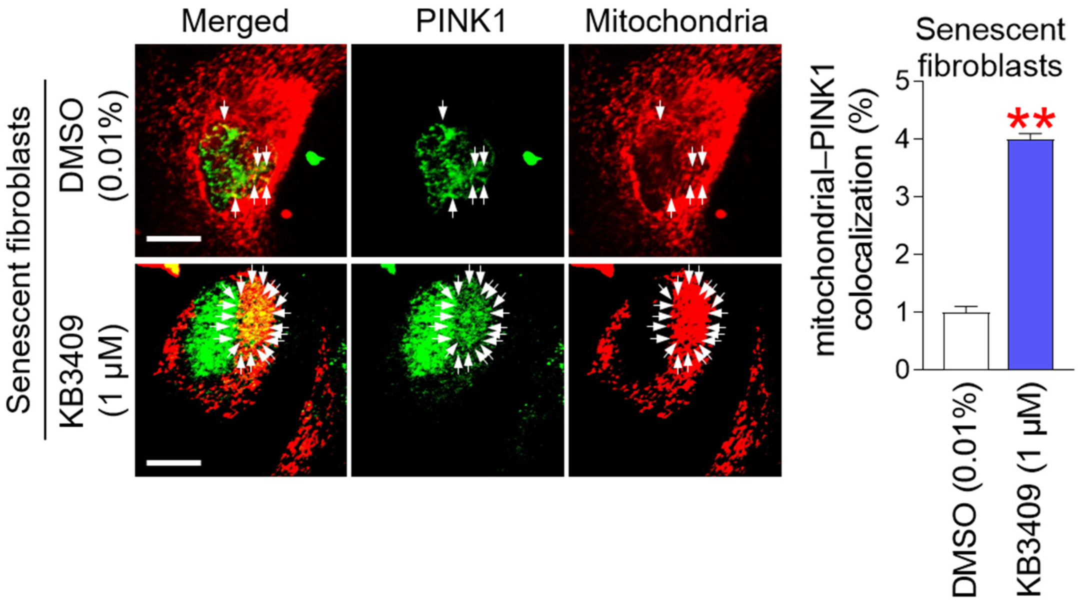

Figure S18. Immunofluorescence analysis of PINK1 (green fluorescence) and mitochondria (red fluorescence) in senescent fibroblasts treated with DMSO (0.01%) or KB3409 (1 μM) for 12 days at 4-day intervals. Scale bar 10 μm. Mitochondrial–PINK1 colocalization is indicated by a white arrow. For statistical analysis, Student's t-test was used, and $^{**}p < 0.01$ was deemed statistically significant. Mean ± standard deviation, $n = 3$.

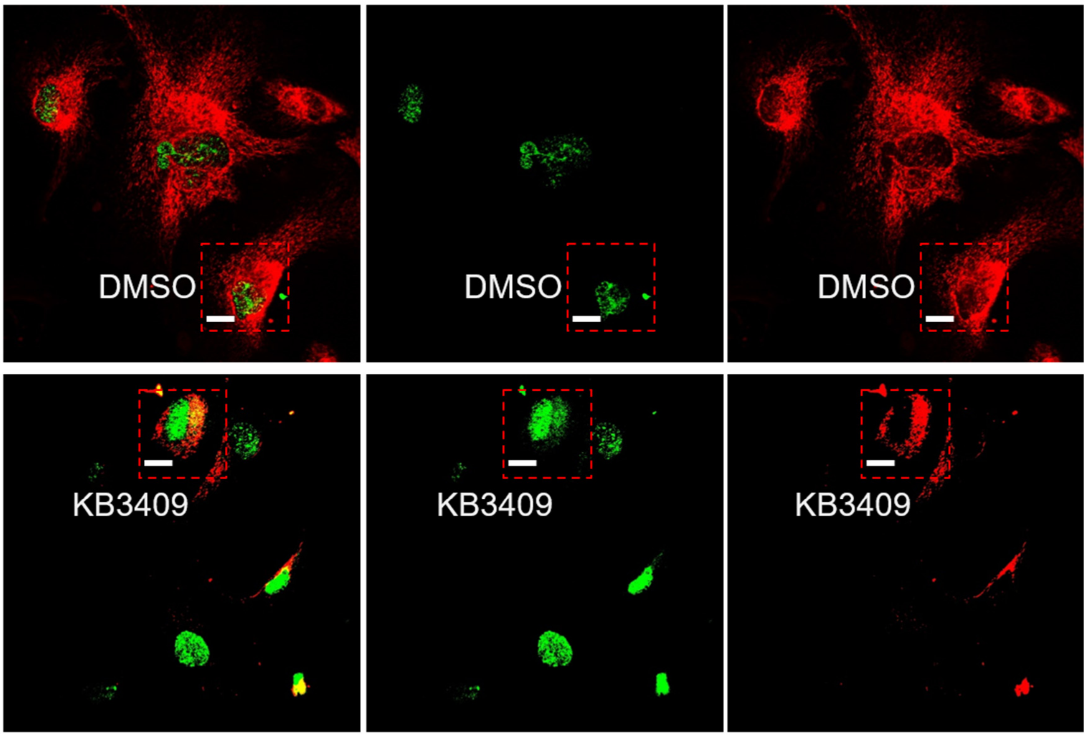

Figure S19. Full-size image of immunofluorescence in Figure S18. Scale bar 10 μm.

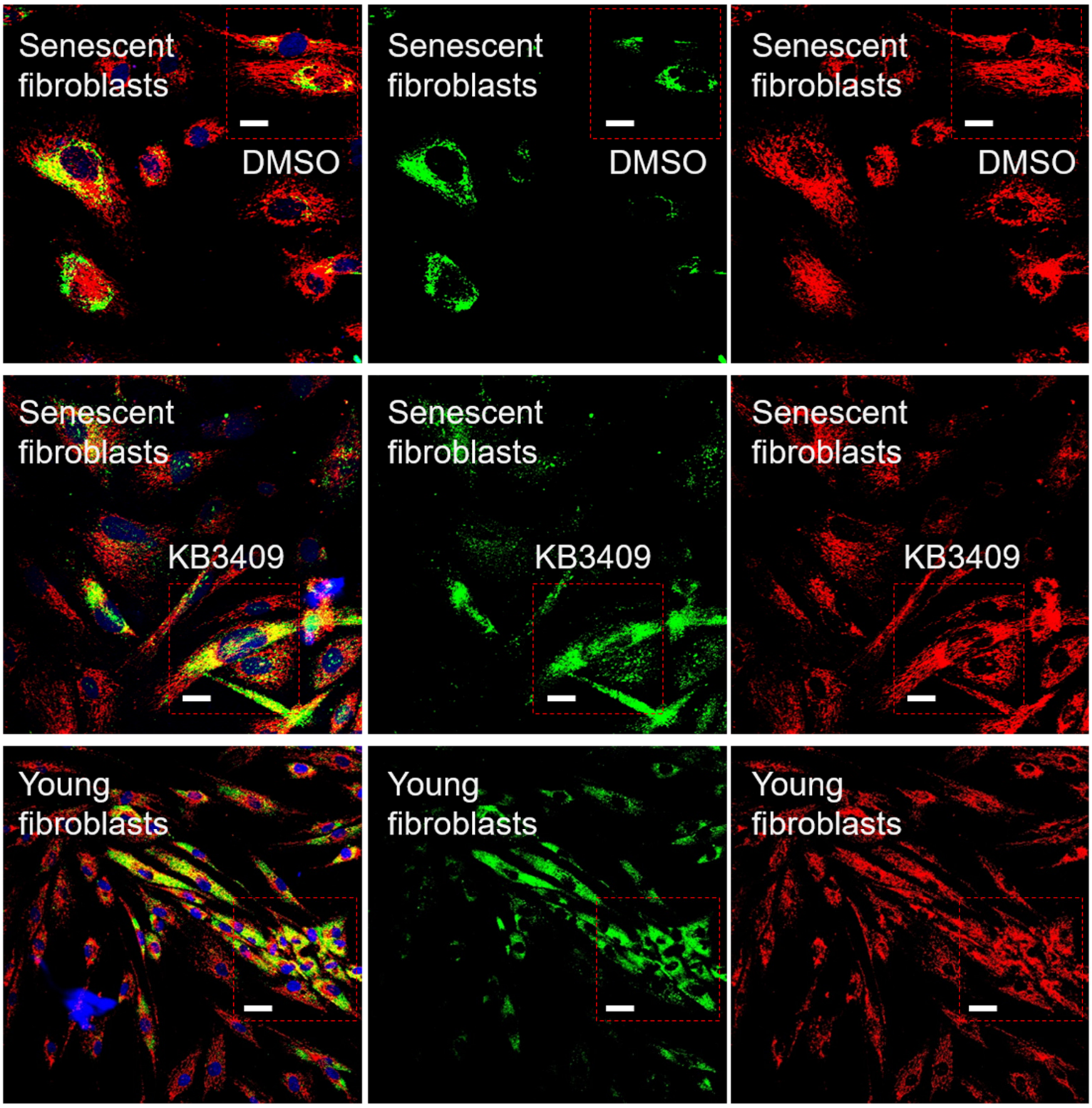

Figure S20. Full-size image of immunofluorescence in Figure 4C. Scale bar 10 μm.

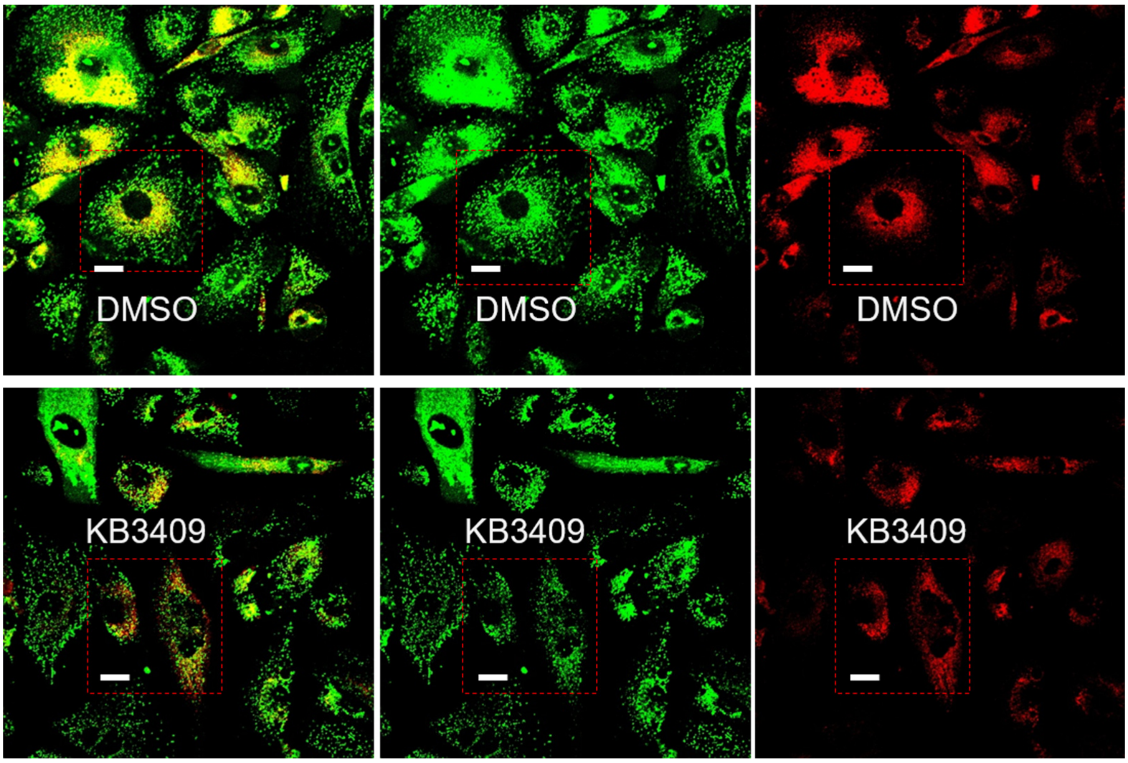

Figure S21. Full-size image of immunofluorescence in Figure 5A. Scale bar 10 μm.

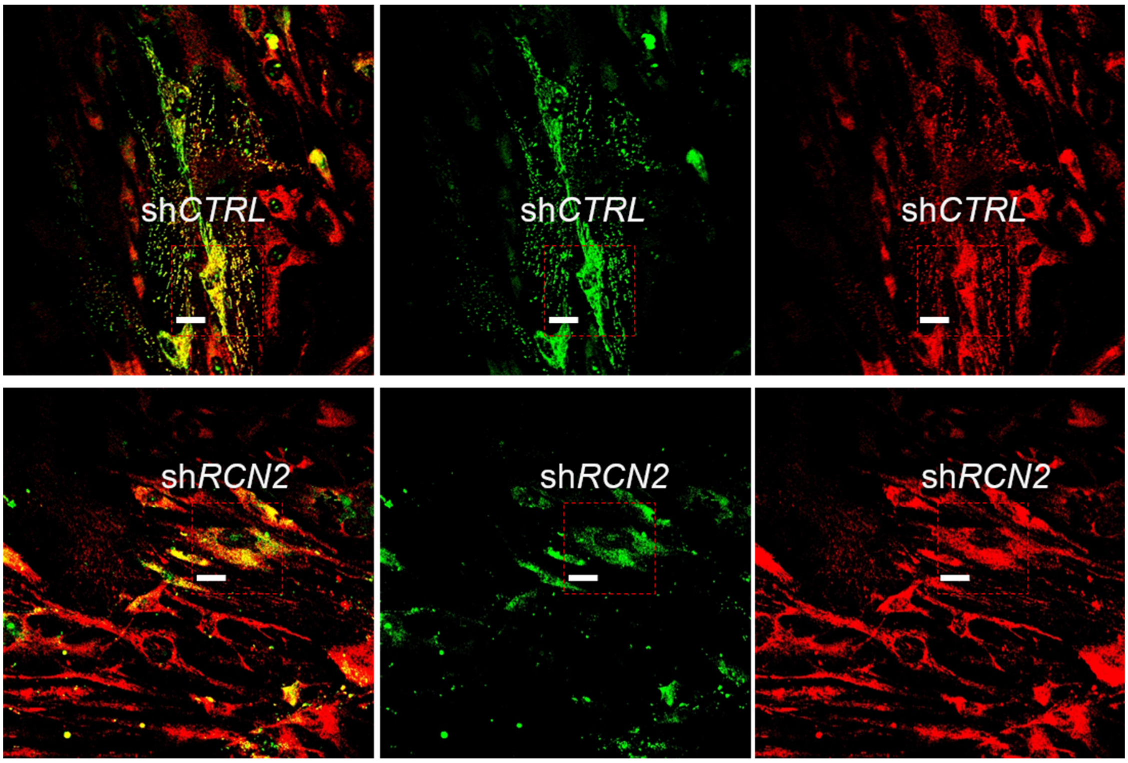

Figure S22. Full-size image of immunofluorescence in Figure 6B. Scale bar 10 µm.

Table S1. Estimated Solubility of the Compounds Predicted Using ChemDraw.

Compound	Molecular weight	Chemdraw log(the estimated solubility expressed in mol/L)	Estimated Solubility (μM)
3409	390.361	-6.174	0.67
2743	361.32	-5.926	1.19
2744	333.266	-3.662	217.77
2745	391.346	-6	1.00
2746	363.292	-3.738	182.81
2747	391.346	-5.969	1.07
2749	379.31	-6.182	0.66
2750	379.31	-6.207	0.62
2751	351.257	-3.92	120.23
2758	404.388	-6.344	0.45
2759	362.308	-5.062	8.67
2761	404.388	-6.303	0.50
3075	302.24	-3.715	192.75
3092	286.24	-4.116	76.56
3093	286.24	-3.797	159.59
3408	390.361	-6.148	0.71
3410	346.308	-4.211	61.52
3412	374.362	-6.467	0.34
3413	374.362	-6.443	0.36
3415	346.308	-4.18	66.07
4302	280.282	-4.69	20.42
4352	413.432	-6.79	0.16
4355	429.474	-6.507	0.31
4514	329.346	-4.383	41.40
4525	353.417	-3.796	159.96
4526	341.381	-4.18	66.07
4528	315.774	-5.189	6.47
4529	327.81	-5.018	9.59
4530	362.255	-5.701	1.99
4700	273.291	-3.027	939.72
4701	287.318	-3.607	247.17
4702	301.344	-4.082	82.79
4703	289.351	-3.803	157.40
4704	303.378	-4.383	41.40

Table S2. The detailed list of ROS level in high throughput screening.

Name	Cell proliferation Value (Normalized by DMSO value)						Average	DHR123 VALUE (Normalized by DMSO value)						ROS Level (DHR123 Value/Cell proliferation contents)						Average	STDEV
DMSO	0.92	0.98	1.00	0.96	1.07	1.08	1.00	0.91	1.05	1.06	0.95	0.94	1.10	0.91	1.05	1.06	0.95	0.94	1.10	1.00	0.08
KB3075	1.12	1.02	1.09	1.05	1.06	1.07	1.07	1.68	1.37	1.44	1.31	1.37	1.49	1.57	1.28	1.35	1.22	1.29	1.39	1.35	0.12
KB3092	1.07	1.04	1.15	1.01	1.04	1.14	1.07	1.64	1.93	1.73	1.57	1.86	2.02	1.53	1.79	1.61	1.46	1.73	1.88	1.67	0.16
KB3093	1.09	0.92	1.07	0.96	1.01	1.07	1.02	2.21	2.03	2.03	1.92	1.92	1.85	2.16	1.99	1.99	1.88	1.88	1.82	1.95	0.12
KB3408	1.07	1.08	0.96	1.00	1.03	1.01	1.02	1.43	1.35	1.28	1.28	1.68	1.16	1.39	1.31	1.25	1.25	1.64	1.13	1.33	0.18
KB3409	1.27	1.08	1.12	1.02	1.09	1.10	1.11	0.86	0.75	0.54	0.57	0.64	0.55	0.78	0.67	0.49	0.51	0.58	0.50	0.59	0.12
KB3410	1.03	0.99	0.96	1.05	1.06	1.02	1.02	1.24	1.51	1.62	1.16	1.44	1.44	1.22	1.48	1.59	1.14	1.41	1.41	1.38	0.17
KB3412	0.94	0.96	0.96	1.03	1.13	1.05	1.01	1.57	1.72	1.65	1.73	1.81	1.80	1.56	1.71	1.63	1.71	1.80	1.78	1.70	0.09
KB3413	1.02	1.02	0.92	1.03	1.06	1.12	1.03	1.81	1.84	1.66	1.59	1.83	1.87	1.76	1.79	1.61	1.54	1.78	1.82	1.72	0.11
KB3415	1.03	0.94	0.94	1.02	1.00	1.00	0.99	1.38	1.40	1.27	1.27	1.27	1.36	1.40	1.41	1.29	1.29	1.29	1.37	1.34	0.06
KB2743	0.97	0.94	1.01	0.98	1.06	1.12	1.01	1.35	1.07	1.36	1.38	1.17	1.41	1.33	1.05	1.34	1.36	1.16	1.39	1.27	0.13
KB2744	1.10	0.92	1.06	1.02	1.08	1.12	1.05	1.60	1.62	1.45	1.75	1.55	1.71	1.53	1.55	1.38	1.67	1.48	1.63	1.54	0.11
KB2745	0.98	1.14	1.09	1.11	1.13	1.20	1.11	1.89	1.87	1.96	2.23	1.82	1.98	1.71	1.69	1.77	2.02	1.64	1.79	1.77	0.13
KB2746	1.04	1.03	1.03	1.01	1.04	1.08	1.04	1.50	1.54	1.56	1.71	1.59	1.40	1.44	1.48	1.51	1.65	1.53	1.35	1.49	0.10
KB2747	0.99	0.95	1.05	1.03	1.03	1.07	1.02	2.08	2.16	2.04	2.34	2.30	2.19	2.04	2.12	2.00	2.30	2.26	2.15	2.14	0.12
KB2749	1.10	1.07	1.02	0.94	0.96	1.18	1.05	1.45	1.30	1.40	1.26	1.40	1.39	1.39	1.25	1.34	1.20	1.34	1.33	1.31	0.07
KB2750	0.95	1.03	1.11	1.04	1.01	1.06	1.03	1.83	1.88	1.92	1.82	1.93	1.91	1.77	1.82	1.86	1.76	1.87	1.85	1.82	0.05
KB2751	1.21	1.03	0.97	1.01	1.04	1.10	1.06	1.64	1.70	1.87	1.49	1.52	1.62	1.54	1.60	1.76	1.41	1.43	1.53	1.55	0.13
KB2758	1.12	0.90	1.00	0.97	1.00	1.05	1.01	1.38	1.16	1.14	1.18	1.27	1.22	1.37	1.15	1.13	1.17	1.26	1.21	1.21	0.09
KB2759	1.16	0.97	0.94	0.94	0.95	0.98	0.99	1.56	1.35	1.33	1.21	1.32	1.31	1.57	1.36	1.34	1.22	1.34	1.32	1.36	0.12
KB2761	0.98	1.07	0.94	1.01	1.08	0.93	1.00	1.81	1.68	1.51	1.65	1.80	1.75	1.81	1.68	1.51	1.65	1.80	1.75	1.70	0.11
KB4302	0.87	0.97	1.01	1.06	1.03	1.02	0.99	1.46	1.29	1.72	1.67	1.33	1.46	1.46	1.30	1.73	1.68	1.34	1.47	1.50	0.17
KB4352	1.22	0.96	0.26	1.05	1.08	1.12	0.95	1.33	1.35	0.62	1.83	1.80	1.54	1.40	1.42	0.65	1.93	1.89	1.62	1.49	0.46
KB4355	1.12	1.13	0.94	0.99	1.09	0.99	1.04	1.57	1.76	1.71	2.17	1.76	1.58	1.51	1.69	1.64	2.08	1.69	1.51	1.69	0.21
KB4514	1.20	1.13	0.96	0.97	0.96	0.97	1.03	1.53	1.34	1.23	1.19	1.15	1.37	1.48	1.30	1.19	1.16	1.12	1.32	1.26	0.13
KB4525	0.98	1.00	1.06	1.12	1.10	1.00	1.04	1.30	1.45	1.50	1.52	1.56	1.37	1.25	1.39	1.43	1.46	1.50	1.31	1.39	0.09
KB4526	1.22	1.06	1.03	1.05	1.11	1.13	1.10	2.06	1.65	1.56	1.54	1.90	1.77	1.87	1.50	1.42	1.40	1.73	1.61	1.59	0.19
KB4528	1.06	0.89	0.99	0.98	0.96	1.13	1.00	1.33	1.16	1.10	1.08	1.06	1.43	1.33	1.16	1.10	1.08	1.06	1.43	1.19	0.15
KB4529	1.01	1.04	0.97	0.96	0.93	0.95	0.98	0.97	1.01	0.89	0.96	0.86	0.92	0.99	1.03	0.91	0.98	0.88	0.94	0.96	0.06
KB4530	1.05	0.89	0.91	0.94	0.90	0.97	0.95	0.97	0.74	0.82	0.77	0.79	0.78	1.02	0.79	0.87	0.81	0.84	0.83	0.86	0.08
KB4700	1.06	1.04	0.99	1.04	1.02	0.97	1.02	1.49	1.43	1.26	1.18	1.20	1.14	1.47	1.41	1.24	1.16	1.18	1.12	1.26	0.14
KB4701	0.96	1.06	0.99	1.05	0.91	1.04	1.00	1.69	1.76	1.47	1.60	1.47	1.53	1.69	1.76	1.47	1.60	1.47	1.53	1.59	0.12
KB4702	1.26	0.96	0.92	0.95	0.93	1.02	1.01	1.69	1.75	1.31	1.25	1.52	1.16	1.68	1.73	1.29	1.24	1.51	1.15	1.43	0.24
KB4703	1.08	0.98	0.96	0.99	1.00	1.01	1.00	1.68	1.48	1.53	1.51	1.52	1.45	1.68	1.48	1.53	1.51	1.52	1.45	1.53	0.08
KB4704	1.08	0.90	0.89	0.96	0.85	0.72	0.90	1.29	0.98	0.92	1.08	0.98	0.80	1.43	1.09	1.02	1.20	1.09	0.89	1.12	0.18

Table S3. List of 43 proteins that bind only to biotinylated KB3409 and not to biotin.

Number	Accession	-10lgP	Avg. Mass	Description
1	P12036	95.25	111838	Neurofilament heavy polypeptide OS=Homo sapiens OX=9606 GN=NEFH PE=1 SV=5
2	Q07065	250.09	66023	Cytoskeleton-associated protein 4 OS=Homo sapiens OX=9606 GN=CKAP4 PE=1 SV=2
3	P46940	101.55	189251	Ras GTPase-activating-like protein IQGAP1 OS=Homo sapiens OX=9606 GN=IQGAP1 PE=1 SV=1
4	Q99878	89.17	13936	Histone H2A type 1-J OS=Homo sapiens OX=9606 GN=H2AC14 PE=1 SV=3
5	Q96KK5	89.17	13906	Histone H2A type 1-H OS=Homo sapiens OX=9606 GN=H2AC12 PE=1 SV=3
6	Q9BTM1	89.17	14019	Histone H2A.J OS=Homo sapiens OX=9606 GN=H2AJ PE=1 SV=1
7	P04908	89.17	14135	Histone H2A type 1-B/E OS=Homo sapiens OX=9606 GN=H2AC4 PE=1 SV=2
8	P0C0S8	89.17	14091	Histone H2A type 1 OS=Homo sapiens OX=9606 GN=H2AC11 PE=1 SV=2
9	P20671	89.17	14107	`
10	Q93077	89.17	14105	Histone H2A type 1-C OS=Homo sapiens OX=9606 GN=H2AC6 PE=1 SV=3
11	Q7L7L0	89.17	14121	Histone H2A type 3 OS=Homo sapiens OX=9606 GN=H2AC25 PE=1 SV=3
12	Q16777	89.17	13988	Histone H2A type 2-C OS=Homo sapiens OX=9606 GN=H2AC20 PE=1 SV=4
13	Q6FI13	89.17	14095	Histone H2A type 2-A OS=Homo sapiens OX=9606 GN=H2AC18 PE=1 SV=3
14	Q9NYL9	127.06	39595	Tropomodulin-3 OS=Homo sapiens OX=9606 GN=TMOD3 PE=1 SV=1
15	P55072	91.9	89322	Transitional endoplasmic reticulum ATPase OS=Homo sapiens OX=9606 GN=VCP PE=1 SV=4
16	P08758	104.43	35937	Annexin A5 OS=Homo sapiens OX=9606 GN=ANXA5 PE=1 SV=2
17	P62987	76.49	14728	Ubiquitin-ribosomal protein eL40 fusion protein OS=Homo sapiens OX=9606 GN=UBA52 PE=1 SV=2
18	P62979	76.49	17965	Ubiquitin-ribosomal protein eS31 fusion protein OS=Homo sapiens OX=9606 GN=RPS27A PE=1 SV=2
19	P0CG47	76.49	25762	Polyubiquitin-B OS=Homo sapiens OX=9606 GN=UBB PE=1 SV=1

20	P0CG48	76.49	77039	Polyubiquitin-C OS=Homo sapiens OX=9606 GN=UBC PE=1 SV=3
21	O75489	109.61	30242	NADH dehydrogenase [ubiquinone] iron-sulfur protein 3 mitochondrial OS=Homo sapiens OX=9606 GN=NDUFS3 PE=1 SV=1
22	P14618	99.18	57937	Pyruvate kinase PKM OS=Homo sapiens OX=9606 GN=PKM PE=1 SV=4
23	P25705	91.76	59751	ATP synthase subunit alpha mitochondrial OS=Homo sapiens OX=9606 GN=ATP5F1A PE=1 SV=1
24	P07355	101.95	38604	Annexin A2 OS=Homo sapiens OX=9606 GN=ANXA2 PE=1 SV=2
25	P07437	106.39	49671	Tubulin beta chain OS=Homo sapiens OX=9606 GN=TUBB PE=1 SV=2
26	P21709	34.48	108127	Ephrin type-A receptor 1 OS=Homo sapiens OX=9606 GN=EPHA1 PE=1 SV=4
27	Q5T9A4	36.03	72573	ATPase family AAA domain-containing protein 3B OS=Homo sapiens OX=9606 GN=ATAD3B PE=1 SV=1
28	Q14257	101.24	36876	Reticulocalbin-2 OS=Homo sapiens OX=9606 GN=RCN2 PE=1 SV=1
29	Q14696	96.62	26077	LRP chaperone MESD OS=Homo sapiens OX=9606 GN=MESD PE=1 SV=2
30	Q7L2H7	79.28	42503	Eukaryotic translation initiation factor 3 subunit M OS=Homo sapiens OX=9606 GN=EIF3M PE=1 SV=1
31	Q9Y2Q5	50.61	13508	Ragulator complex protein LAMTOR2 OS=Homo sapiens OX=9606 GN=LAMTOR2 PE=1 SV=1
32	O14924	32.11	156357	Regulator of G-protein signaling 12 OS=Homo sapiens OX=9606 GN=RGS12 PE=1 SV=1
33	Q7Z3V4	29.58	123098	Ubiquitin-protein ligase E3B OS=Homo sapiens OX=9606 GN=UBE3B PE=1 SV=3
34	P21980	35.3	77329	Protein-glutamine gamma-glutamyltransferase 2 OS=Homo sapiens OX=9606 GN=TGM2 PE=1 SV=2
35	O00469	61.27	84686	Procollagen-lysine 2-oxoglutarate 5-dioxygenase 2 OS=Homo sapiens OX=9606 GN=PLOD2 PE=1 SV=2
36	P50454	33.85	46441	Serpin H1 OS=Homo sapiens OX=9606 GN=SERPINH1 PE=1 SV=2
37	P62888	81.1	12784	Large ribosomal subunit protein eL30 OS=Homo sapiens OX=9606 GN=RPL30 PE=1 SV=2
38	Q12797	78.23	85863	Aspartyl/asparaginyl beta-hydroxylase OS=Homo sapiens OX=9606 GN=ASPH PE=1 SV=3
39	Q5T2N8	75.37	46380	ATPase family AAA domain-containing protein 3C OS=Homo sapiens OX=9606 GN=ATAD3C PE=1

				SV=2
40	Q9NVI7	75.37	71369	ATPase family AAA domain-containing protein 3A OS=Homo sapiens OX=9606 GN=ATAD3A PE=1 SV=2
41	P00352	62.03	54862	Aldehyde dehydrogenase 1A1 OS=Homo sapiens OX=9606 GN=ALDH1A1 PE=1 SV=2
42	O75380	47.59	13712	NADH dehydrogenase [ubiquinone] iron-sulfur protein 6 mitochondrial OS=Homo sapiens OX=9606 GN=NDUFS6 PE=1 SV=1
43	P61353	29.74	15798	Large ribosomal subunit protein eL27 OS=Homo sapiens OX=9606 GN=RPL27 PE=1 SV=2

Table S4. List of 95 proteins that bind only to biotin and not to biotinylated KB3409.

Number	Accession	-10lgP	Avg. Mass	Description
1	P04259	270.26	60067	Keratin type II cytoskeletal 6B OS=Homo sapiens OX=9606 GN=KRT6B PE=1 SV=5
2	P08779	232.48	51268	Keratin type I cytoskeletal 16 OS=Homo sapiens OX=9606 GN=KRT16 PE=1 SV=4
3	P48668	278.94	60025	Keratin type II cytoskeletal 6C OS=Homo sapiens OX=9606 GN=KRT6C PE=1 SV=3
4	P02538	277.65	60045	Keratin type II cytoskeletal 6A OS=Homo sapiens OX=9606 GN=KRT6A PE=1 SV=3
5	Q04695	212.85	48106	Keratin type I cytoskeletal 17 OS=Homo sapiens OX=9606 GN=KRT17 PE=1 SV=2
6	P08727	122.53	44106	Keratin type I cytoskeletal 19 OS=Homo sapiens OX=9606 GN=KRT19 PE=1 SV=4
7	P13646	125.12	49588	Keratin type I cytoskeletal 13 OS=Homo sapiens OX=9606 GN=KRT13 PE=1 SV=4
8	Q01546	133.76	65841	Keratin type II cytoskeletal 2 oral OS=Homo sapiens OX=9606 GN=KRT76 PE=1 SV=2
9	P13647	214.06	62378	Keratin type II cytoskeletal 5 OS=Homo sapiens OX=9606 GN=KRT5 PE=1 SV=3
10	P05783	96.13	48058	Keratin type I cytoskeletal 18 OS=Homo sapiens OX=9606 GN=KRT18 PE=1 SV=2
11	P19013	108.46	56144	Keratin type II cytoskeletal 4 OS=Homo sapiens OX=9606 GN=KRT4 PE=1 SV=5
12	P12035	137.26	64417	Keratin type II cytoskeletal 3 OS=Homo sapiens OX=9606 GN=KRT3 PE=1 SV=3
13	P43235	182.68	36966	Cathepsin K OS=Homo sapiens OX=9606 GN=CTSK PE=1 SV=1
14	P62913	149.6	20252	Large ribosomal subunit protein uL5 OS=Homo sapiens OX=9606 GN=RPL11 PE=1 SV=2
15	Q99623	136.71	33296	Prohibitin-2 OS=Homo sapiens OX=9606 GN=PHB2 PE=1 SV=2
16	P62807	148.2	13906	Histone H2B type 1-C/E/F/G/I OS=Homo sapiens OX=9606 GN=H2BC4 PE=1 SV=4
17	Q99879	148.2	13989	Histone H2B type 1-M OS=Homo sapiens OX=9606 GN=H2BC14 PE=1 SV=3
18	Q93079	148.2	13892	Histone H2B type 1-H OS=Homo sapiens OX=9606 GN=H2BC9 PE=1 SV=3
19	P58876	148.2	13936	Histone H2B type 1-D OS=Homo sapiens OX=9606 GN=H2BC5 PE=1 SV=2
20	Q5QNW6	148.2	13920	Histone H2B type 2-F OS=Homo sapiens OX=9606 GN=H2BC18 PE=1 SV=3
21	Q99877	148.2	13922	Histone H2B type 1-N OS=Homo sapiens OX=9606 GN=H2BC15 PE=1 SV=3

22	O60814	148.2	13890	Histone H2B type 1-K OS=Homo sapiens OX=9606 GN=H2BC12 PE=1 SV=3
23	P57053	148.2	13944	Histone H2B type F-S OS=Homo sapiens OX=9606 GN=H2BC12L PE=1 SV=2
24	Q99880	148.2	13952	Histone H2B type 1-L OS=Homo sapiens OX=9606 GN=H2BC13 PE=1 SV=3
25	P41091	174.44	51110	Eukaryotic translation initiation factor 2 subunit 3 OS=Homo sapiens OX=9606 GN=EIF2S3 PE=1 SV=3
26	P04792	154.06	22783	Heat shock protein beta-1 OS=Homo sapiens OX=9606 GN=HSPB1 PE=1 SV=2
27	P04080	138.97	11140	Cystatin-B OS=Homo sapiens OX=9606 GN=CSTB PE=1 SV=2
28	P09234	109.04	17394	U1 small nuclear ribonucleoprotein C OS=Homo sapiens OX=9606 GN=SNRPC PE=1 SV=1
29	Q96DA6	124.08	12499	Mitochondrial import inner membrane translocase subunit TIM14 OS=Homo sapiens OX=9606 GN=DNAJC19 PE=1 SV=3
30	P62269	96.46	17719	Small ribosomal subunit protein uS13 OS=Homo sapiens OX=9606 GN=RPS18 PE=1 SV=3
31	Q5U7I5	82.01	15872	Transthyretin OS=Pan troglodytes OX=9598 GN=TTR PE=2 SV=1
32	Q99470	105.66	23026	Stromal cell-derived factor 2 OS=Homo sapiens OX=9606 GN=SDF2 PE=1 SV=2
33	P46782	101.62	22876	Small ribosomal subunit protein uS7 OS=Homo sapiens OX=9606 GN=RPS5 PE=1 SV=4
34	P39019	140.96	16060	Small ribosomal subunit protein eS19 OS=Homo sapiens OX=9606 GN=RPS19 PE=1 SV=2
35	P05204	120.35	9393	Non-histone chromosomal protein HMG-17 OS=Homo sapiens OX=9606 GN=HMGN2 PE=1 SV=3
36	P25398	96.11	14515	Small ribosomal subunit protein eS12 OS=Homo sapiens OX=9606 GN=RPS12 PE=1 SV=3
37	P07093	58.16	44002	Glia-derived nexin OS=Homo sapiens OX=9606 GN=SERPINE2 PE=1 SV=1
38	O75323	67.75	33743	Protein NipSnap homolog 2 OS=Homo sapiens OX=9606 GN=NIPSNAP2 PE=1 SV=1
39	Q7RTV0	91.82	12405	PHD finger-like domain-containing protein 5A OS=Homo sapiens OX=9606 GN=PHF5A PE=1 SV=1
40	Q9Y3D7	58.62	13825	Mitochondrial import inner membrane translocase subunit TIM16 OS=Homo sapiens

				OX=9606 GN=PAM16 PE=1 SV=2
41	P62081	80.25	22127	Small ribosomal subunit protein eS7 OS=Homo sapiens OX=9606 GN=RPS7 PE=1 SV=1
42	O95139	54.79	15489	NADH dehydrogenase [ubiquinone] 1 beta subcomplex subunit 6 OS=Homo sapiens OX=9606 GN=NDUFB6 PE=1 SV=3
43	Q96AG4	63.37	34930	Leucine-rich repeat-containing protein 59 OS=Homo sapiens OX=9606 GN=LRRC59 PE=1 SV=1
44	P04406	129.85	36053	Glyceraldehyde-3-phosphate dehydrogenase OS=Homo sapiens OX=9606 GN=GAPDH PE=1 SV=3
45	Q15388	47.07	16298	Mitochondrial import receptor subunit TOM20 homolog OS=Homo sapiens OX=9606 GN=TOMM20 PE=1 SV=1
46	P62899	39.24	14463	Large ribosomal subunit protein eL31 OS=Homo sapiens OX=9606 GN=RPL31 PE=1 SV=1
47	P14678	69.73	24610	Small nuclear ribonucleoprotein-associated proteins B and B' OS=Homo sapiens OX=9606 GN=SNRPB PE=1 SV=2
48	P63162	69.73	24614	Small nuclear ribonucleoprotein-associated protein N OS=Homo sapiens OX=9606 GN=SNRPN PE=1 SV=1
49	Q9UHD8	70.95	65402	Septin-9 OS=Homo sapiens OX=9606 GN=SEPTIN9 PE=1 SV=2
50	B2RPK0	91.52	24238	Putative high mobility group protein B1-like 1 OS=Homo sapiens OX=9606 GN=HMGB1P1 PE=5 SV=1
51	P09429	91.52	24894	High mobility group protein B1 OS=Homo sapiens OX=9606 GN=HMGB1 PE=1 SV=3
52	Q14247	54.2	61586	Src substrate cortactin OS=Homo sapiens OX=9606 GN=CTTN PE=1 SV=2
53	P06733	135.8	47169	Alpha-enolase OS=Homo sapiens OX=9606 GN=ENO1 PE=1 SV=2
54	Q6NZI2	101.4	43476	Caveolae-associated protein 1 OS=Homo sapiens OX=9606 GN=CAVIN1 PE=1 SV=1
55	P06748	51.42	32575	Nucleophosmin OS=Homo sapiens OX=9606 GN=NPM1 PE=1 SV=2
56	Q15149	95.32	531796	Plectin OS=Homo sapiens OX=9606 GN=PLEC PE=1 SV=3
57	P08579	55.7	25486	U2 small nuclear ribonucleoprotein B'' OS=Homo sapiens OX=9606 GN=SNRPB2 PE=1 SV=1
58	Q9NYF8	61.03	106122	Bcl-2-associated transcription factor 1 OS=Homo sapiens OX=9606 GN=BCLAF1

				PE=1 SV=2
59	Q16181	65.45	50680	Septin-7 OS=Homo sapiens OX=9606 GN=SEPTIN7 PE=1 SV=2
60	P62937	118.47	18012	Peptidyl-prolyl cis-trans isomerase A OS=Homo sapiens OX=9606 GN=PPIA PE=1 SV=2
61	P47813	73.47	16460	Eukaryotic translation initiation factor 1A X-chromosomal OS=Homo sapiens OX=9606 GN=EIF1AX PE=1 SV=2
62	O14602	73.47	16442	Eukaryotic translation initiation factor 1A Y-chromosomal OS=Homo sapiens OX=9606 GN=EIF1AY PE=1 SV=4
63	P31946	96.31	28082	14-3-3 protein beta/alpha OS=Homo sapiens OX=9606 GN=YWHAB PE=1 SV=3
64	Q9UII2	37.12	12249	ATPase inhibitor mitochondrial OS=Homo sapiens OX=9606 GN=ATP5IF1 PE=1 SV=1
65	Q09666	69.18	629114	Neuroblast differentiation-associated protein AHNAK OS=Homo sapiens OX=9606 GN=AHNAK PE=1 SV=2
66	P27348	77.9	27764	14-3-3 protein theta OS=Homo sapiens OX=9606 GN=YWHAQ PE=1 SV=1
67	O00422	39.32	17561	Histone deacetylase complex subunit SAP18 OS=Homo sapiens OX=9606 GN=SAP18 PE=1 SV=1
68	Q9UJ98	30.26	139033	Cohesin subunit SA-3 OS=Homo sapiens OX=9606 GN=STAG3 PE=1 SV=2
69	P62258	89.59	29174	14-3-3 protein epsilon OS=Homo sapiens OX=9606 GN=YWHAE PE=1 SV=1
70	P19404	70.73	27392	NADH dehydrogenase [ubiquinone] flavoprotein 2 mitochondrial OS=Homo sapiens OX=9606 GN=NDUFV2 PE=1 SV=2
71	O95336	63.36	27547	6-phosphogluconolactonase OS=Homo sapiens OX=9606 GN=PGLS PE=1 SV=2
72	Q5VSP4	54.49	17918	Putative lipocalin 1-like protein 1 OS=Homo sapiens OX=9606 GN=LCN1P1 PE=5 SV=1
73	P31025	54.49	19250	Lipocalin-1 OS=Homo sapiens OX=9606 GN=LCN1 PE=1 SV=1
74	P08238	34.43	83264	Heat shock protein HSP 90-beta OS=Homo sapiens OX=9606 GN=HSP90AB1 PE=1 SV=4
75	P46821	31.48	270632	Microtubule-associated protein 1B OS=Homo sapiens OX=9606 GN=MAP1B PE=1 SV=2
76	P09874	64.02	113084	Poly [ADP-ribose] polymerase 1 OS=Homo sapiens OX=9606 GN=PARP1 PE=1 SV=4
77	Q9NVA2	55.96	49398	Septin-11 OS=Homo sapiens OX=9606 GN=SEPTIN11 PE=1 SV=3

78	P61981	92.24	28303	14-3-3 protein gamma OS=Homo sapiens OX=9606 GN=YWHAG PE=1 SV=2
79	E9PAV3	85.17	205419	Nascent polypeptide-associated complex subunit alpha muscle-specific form OS=Homo sapiens OX=9606 GN=NACA PE=1 SV=1
80	Q13765	85.17	23384	Nascent polypeptide-associated complex subunit alpha OS=Homo sapiens OX=9606 GN=NACA PE=1 SV=1
81	Q9Y2G1	28.72	124397	Myelin regulatory factor OS=Homo sapiens OX=9606 GN=MYRF PE=1 SV=3
82	Q9NX63	42.71	26152	MICOS complex subunit MIC19 OS=Homo sapiens OX=9606 GN=CHCHD3 PE=1 SV=1
83	Q16270	30.99	29130	Insulin-like growth factor-binding protein 7 OS=Homo sapiens OX=9606 GN=IGFBP7 PE=1 SV=1
84	P37108	29.23	14570	Signal recognition particle 14 kDa protein OS=Homo sapiens OX=9606 GN=SRP14 PE=1 SV=2
85	P40429	40.41	23577	Large ribosomal subunit protein uL13 OS=Homo sapiens OX=9606 GN=RPL13A PE=1 SV=2
86	P19338	29.01	76615	Nucleolin OS=Homo sapiens OX=9606 GN=NCL PE=1 SV=3
87	Q13813	57.65	284538	Spectrin alpha chain non-erythrocytic 1 OS=Homo sapiens OX=9606 GN=SPTAN1 PE=1 SV=3
88	Q9Y4L1	73.35	111335	Hypoxia up-regulated protein 1 OS=Homo sapiens OX=9606 GN=HYOU1 PE=1 SV=1
89	P10809	58.51	61055	60 kDa heat shock protein mitochondrial OS=Homo sapiens OX=9606 GN=HSPD1 PE=1 SV=2
90	P08123	53.17	129314	Collagen alpha-2(I) chain OS=Homo sapiens OX=9606 GN=COL1A2 PE=1 SV=7
91	P98082	35.67	82448	Disabled homolog 2 OS=Homo sapiens OX=9606 GN=DAB2 PE=1 SV=3
92	Q15057	31.74	88029	Arf-GAP with coiled-coil ANK repeat and PH domain-containing protein 2 OS=Homo sapiens OX=9606 GN=ACAP2 PE=1 SV=3
93	Q9H9T3	29.75	62259	Elongator complex protein 3 OS=Homo sapiens OX=9606 GN=ELP3 PE=1 SV=2
94	F5HC97	29.32	46263	DNA polymerase processivity factor OS=Human cytomegalovirus (strain Merlin) OX=295027 GN=UL44 PE=3 SV=1
95	P16790	29.32	46233	DNA polymerase processivity factor OS=Human cytomegalovirus (strain AD169) OX=10360 GN=UL44 PE=1 SV=1

Table S5. List of 82 proteins that bind to both biotinylated KB3409 and biotin.

Num ber	Accession	-10lgP	Avg. Mass	Description
1	P08670	442.41	53652	Vimentin OS=Homo sapiens OX=9606 GN=VIM PE=1 SV=4
2	P60709	361.29	41737	Actin cytoplasmic 1 OS=Homo sapiens OX=9606 GN=ACTB PE=1 SV=1
3	P63261	360.55	41793	Actin cytoplasmic 2 OS=Homo sapiens OX=9606 GN=ACTG1 PE=1 SV=1
4	P04264	375.21	66039	Keratin type II cytoskeletal 1 OS=Homo sapiens OX=9606 GN=KRT1 PE=1 SV=6
5	P13645	371.86	58827	Keratin type I cytoskeletal 10 OS=Homo sapiens OX=9606 GN=KRT10 PE=1 SV=6
6	P63267	296.03	41877	Actin gamma-enteric smooth muscle OS=Homo sapiens OX=9606 GN=ACTG2 PE=1 SV=1
7	P62736	296.03	42009	Actin aortic smooth muscle OS=Homo sapiens OX=9606 GN=ACTA2 PE=1 SV=1
8	P68133	296.03	42051	Actin alpha skeletal muscle OS=Homo sapiens OX=9606 GN=ACTA1 PE=1 SV=1
9	P68032	296.03	42019	Actin alpha cardiac muscle 1 OS=Homo sapiens OX=9606 GN=ACTC1 PE=1 SV=1
10	P35527	318.46	62064	Keratin type I cytoskeletal 9 OS=Homo sapiens OX=9606 GN=KRT9 PE=1 SV=3
11	P02768	236.71	69367	Albumin OS=Homo sapiens OX=9606 GN=ALB PE=1 SV=2
12	P27487	305.1	88279	Dipeptidyl peptidase 4 OS=Homo sapiens OX=9606 GN=DPP4 PE=1 SV=2
13	P35579	466.16	226530	Myosin-9 OS=Homo sapiens OX=9606 GN=MYH9 PE=1 SV=4
14	P23284	278.76	23743	Peptidyl-prolyl cis-trans isomerase B OS=Homo sapiens OX=9606 GN=PPIB PE=1 SV=2

15	P35908	321.24	65433	Keratin type II cytoskeletal 2 epidermal OS=Homo sapiens OX=9606 GN=KRT2 PE=1 SV=2
16	Q6S8J3	232.58	121363	POTE ankyrin domain family member E OS=Homo sapiens OX=9606 GN=POTEE PE=1 SV=3
17	Q562R1	231.16	42003	Beta-actin-like protein 2 OS=Homo sapiens OX=9606 GN=ACTBL2 PE=1 SV=2
18	P02533	242.03	51562	Keratin type I cytoskeletal 14 OS=Homo sapiens OX=9606 GN=KRT14 PE=1 SV=4
19	P17661	167.2	53536	Desmin OS=Homo sapiens OX=9606 GN=DES PE=1 SV=3
20	P35268	187.27	14787	Large ribosomal subunit protein eL22 OS=Homo sapiens OX=9606 GN=RPL22 PE=1 SV=2
21	Q9P2E9	329.45	152456	Ribosome-binding protein 1 OS=Homo sapiens OX=9606 GN=RRBP1 PE=1 SV=5
22	P05787	122.87	53704	Keratin type II cytoskeletal 8 OS=Homo sapiens OX=9606 GN=KRT8 PE=1 SV=7
23	Q14956	125.69	63923	Transmembrane glycoprotein NMB OS=Homo sapiens OX=9606 GN=GPNMB PE=1 SV=2
24	Q7Z794	150.05	61901	Keratin type II cytoskeletal 1b OS=Homo sapiens OX=9606 GN=KRT77 PE=1 SV=3
25	P09012	146.3	31280	U1 small nuclear ribonucleoprotein A OS=Homo sapiens OX=9606 GN=SNRPA PE=1 SV=3
26	P60660	208.97	16930	Myosin light polypeptide 6 OS=Homo sapiens OX=9606 GN=MYL6 PE=1 SV=2
27	P67936	169.19	28522	Tropomyosin alpha-4 chain OS=Homo sapiens OX=9606 GN=TPM4 PE=1 SV=3
28	P62318	169.17	13916	Small nuclear ribonucleoprotein Sm D3 OS=Homo sapiens OX=9606 GN=SNRPD3 PE=1

				SV=1
29	P19105	239.54	19794	Myosin regulatory light chain 12A OS=Homo sapiens OX=9606 GN=MYL12A PE=1 SV=2
30	O14950	239.54	19779	Myosin regulatory light chain 12B OS=Homo sapiens OX=9606 GN=MYL12B PE=1 SV=2
31	P09493	209.41	32709	Tropomyosin alpha-1 chain OS=Homo sapiens OX=9606 GN=TPM1 PE=1 SV=2
32	P07951	198.07	32851	Tropomyosin beta chain OS=Homo sapiens OX=9606 GN=TPM2 PE=1 SV=1
33	P06576	225.92	56560	ATP synthase subunit beta mitochondrial OS=Homo sapiens OX=9606 GN=ATP5F1B PE=1 SV=3
34	P24844	129.58	19827	Myosin regulatory light polypeptide 9 OS=Homo sapiens OX=9606 GN=MYL9 PE=1 SV=4
35	P84090	137.72	12259	Enhancer of rudimentary homolog OS=Homo sapiens OX=9606 GN=ERH PE=1 SV=1
36	P68104	144.59	50141	Elongation factor 1-alpha 1 OS=Homo sapiens OX=9606 GN=EEF1A1 PE=1 SV=1
37	Q5VTE0	144.59	50185	Putative elongation factor 1-alpha-like 3 OS=Homo sapiens OX=9606 GN=EEF1A1P5 PE=5 SV=1
38	P02751	240.68	272318	Fibronectin OS=Homo sapiens OX=9606 GN=FN1 PE=1 SV=5
39	Q86Y46	117.75	58923	Keratin type II cytoskeletal 73 OS=Homo sapiens OX=9606 GN=KRT73 PE=1 SV=1
40	P62750	191.28	17695	Large ribosomal subunit protein uL23 OS=Homo sapiens OX=9606 GN=RPL23A PE=1 SV=1
41	P35580	211.66	228997	Myosin-10 OS=Homo sapiens OX=9606 GN=MYH10 PE=1 SV=3

42	P06753	150.45	32950	Tropomyosin alpha-3 chain OS=Homo sapiens OX=9606 GN=TPM3 PE=1 SV=2
43	P23396	181.77	26688	Small ribosomal subunit protein uS3 OS=Homo sapiens OX=9606 GN=RPS3 PE=1 SV=2
44	P05198	115.61	36112	Eukaryotic translation initiation factor 2 subunit 1 OS=Homo sapiens OX=9606 GN=EIF2S1 PE=1 SV=3
45	Q9BPW8	121.13	33310	Protein NipSnap homolog 1 OS=Homo sapiens OX=9606 GN=NIPSNAP1 PE=1 SV=1
46	P62249	107.15	16445	Small ribosomal subunit protein uS9 OS=Homo sapiens OX=9606 GN=RPS16 PE=1 SV=2
47	P62306	99.75	9725	Small nuclear ribonucleoprotein F OS=Homo sapiens OX=9606 GN=SNRPF PE=1 SV=1
48	P62826	97.44	24423	GTP-binding nuclear protein Ran OS=Homo sapiens OX=9606 GN=RAN PE=1 SV=3
49	Q92804	160.77	61830	TATA-binding protein-associated factor 2N OS=Homo sapiens OX=9606 GN=TAF15 PE=1 SV=1
50	Q15155	231.01	134324	BOS complex subunit NOMO1 OS=Homo sapiens OX=9606 GN=NOMO1 PE=1 SV=5
51	P69849	231.01	134133	BOS complex subunit NOMO3 OS=Homo sapiens OX=9606 GN=NOMO3 PE=1 SV=2
52	Q5JPE7	231.01	139439	BOS complex subunit NOMO2 OS=Homo sapiens OX=9606 GN=NOMO2 PE=1 SV=1
53	Q14CN4	122.48	55877	Keratin type II cytoskeletal 72 OS=Homo sapiens OX=9606 GN=KRT72 PE=1 SV=2
54	Q15287	108.42	34208	RNA-binding protein with serine-rich domain 1 OS=Homo sapiens OX=9606 GN=RNPS1 PE=1 SV=1
55	P32969	196.29	21863	Large ribosomal subunit protein uL6 OS=Homo sapiens

				OX=9606 GN=RPL9 PE=1 SV=1
56	Q5IFJ7	196.29	21877	Large ribosomal subunit protein uL6 OS=Macaca fascicularis OX=9541 GN=RPL9 PE=2 SV=1
57	P62241	141.9	24205	Small ribosomal subunit protein eS8 OS=Homo sapiens OX=9606 GN=RPS8 PE=1 SV=2
58	P11021	198.22	72333	Endoplasmic reticulum chaperone BiP OS=Homo sapiens OX=9606 GN=HSPA5 PE=1 SV=2
59	P02545	148.92	74140	Prelamin-A/C OS=Homo sapiens OX=9606 GN=LMNA PE=1 SV=1
60	P47756	122.08	30629	F-actin-capping protein subunit beta OS=Homo sapiens OX=9606 GN=CAPZB PE=1 SV=5
61	P35232	133.42	29804	Prohibitin 1 OS=Homo sapiens OX=9606 GN=PHB1 PE=1 SV=1
62	Q0ZGT2	104.92	80658	Nexilin OS=Homo sapiens OX=9606 GN=NEXN PE=1 SV=1
63	P49458	126.83	10112	Signal recognition particle 9 kDa protein OS=Homo sapiens OX=9606 GN=SRP9 PE=1 SV=2
64	P06396	130.17	85697	Gelsolin OS=Homo sapiens OX=9606 GN=GSN PE=1 SV=1
65	P52907	102.35	32923	F-actin-capping protein subunit alpha-1 OS=Homo sapiens OX=9606 GN=CAPZA1 PE=1 SV=3
66	Q86YZ3	77.49	282389	Hornerin OS=Homo sapiens OX=9606 GN=HRNR PE=1 SV=2
67	P62701	95.67	29598	Small ribosomal subunit protein eS4 X isoform OS=Homo sapiens OX=9606 GN=RPS4X PE=1 SV=2
68	Q15393	115.7	135577	Splicing factor 3B subunit 3 OS=Homo sapiens OX=9606

				GN=SF3B3 PE=1 SV=4
69	Q9BQE3	135.01	49895	Tubulin alpha-1C chain OS=Homo sapiens OX=9606 GN=TUBA1C PE=1 SV=1
70	Q71U36	135.01	50136	Tubulin alpha-1A chain OS=Homo sapiens OX=9606 GN=TUBA1A PE=1 SV=1
71	P68363	135.01	50152	Tubulin alpha-1B chain OS=Homo sapiens OX=9606 GN=TUBA1B PE=1 SV=1
72	P62308	82.6	8496	Small nuclear ribonucleoprotein G OS=Homo sapiens OX=9606 GN=SNRPG PE=1 SV=1
73	P39023	86.39	46109	Large ribosomal subunit protein uL3 OS=Homo sapiens OX=9606 GN=RPL3 PE=1 SV=2
74	P61247	85.09	29945	Small ribosomal subunit protein eS1 OS=Homo sapiens OX=9606 GN=RPS3A PE=1 SV=2
75	P47755	123.01	32949	F-actin-capping protein subunit alpha-2 OS=Homo sapiens OX=9606 GN=CAPZA2 PE=1 SV=3
76	O00159	121.61	121682	Unconventional myosin-Ic OS=Homo sapiens OX=9606 GN=MYO1C PE=1 SV=4
77	P62316	74.55	13527	Small nuclear ribonucleoprotein Sm D2 OS=Homo sapiens OX=9606 GN=SNRPD2 PE=1 SV=1
78	P23528	72.62	18502	Cofilin-1 OS=Homo sapiens OX=9606 GN=CFL1 PE=1 SV=3
79	P11142	114.56	70898	Heat shock cognate 71 kDa protein OS=Homo sapiens OX=9606 GN=HSPA8 PE=1 SV=1
80	P15144	62.81	109539	Aminopeptidase N OS=Homo sapiens OX=9606 GN=ANPEP PE=1 SV=4
81	Q14315	46.46	291020	Filamin-C OS=Homo sapiens OX=9606 GN=FLNC PE=1 SV=3
82	P36956	28.69	121675	Sterol regulatory element-binding protein 1 OS=Homo

	sapiens OX=9606 GN=SREBF1 PE=1 SV=2
